# Glycaemic control and variability with different commercially available hybrid closed loop systems in people with type 1 diabetes: A systematic review and meta‐analysis of randomized controlled trials

**DOI:** 10.1111/dom.70150

**Published:** 2025-09-29

**Authors:** Sergio Di Molfetta, Ludovico Di Gioia, Irene Caruso, Mariangela Caporusso, Paolo Trerotoli, Annalisa Natalicchio, Sebastio Perrini, Luigi Laviola, Francesco Giorgino

**Affiliations:** ^1^ Endocrinology Unit University Hospital “Consorziale Policlinico” of Bari Bari Italy; ^2^ Department of Precision and Regenerative Medicine and Ionian Area, Section of Internal Medicine, Endocrinology, Andrology and Metabolic Diseases University of Bari Aldo Moro Bari Italy; ^3^ Endocrinology Unit Regional General Hospital “Francesco Miulli” Bari Italy; ^4^ Interdisciplinary Department of Medicine, Hygiene Section University of Bari Aldo Moro Bari Italy; ^5^ Section of Endocrinology, Department of Medicine and Surgery Libera Università Mediterranea (LUM) University Bari Italy

**Keywords:** adults, hybrid closed loop, insulin therapy, meta‐analysis, time in range, type 1 diabetes, youth

## Abstract

**Aims:**

To provide an updated analysis of the performance of different hybrid closed loop (HCL) systems in randomised controlled trials (RCTs) on subjects with type 1 diabetes.

**Materials and methods:**

We conducted a systematic review with meta‐analysis. We searched four online databases and performed hand‐searching of conference proceedings to find studies from inception to 18 April 2025. We included RCTs enrolling subjects with type 1 diabetes, evaluating commercial HCL systems against other insulin therapy regimens, with a duration of intervention ≥2 weeks, and reporting time in range (TIR) as an outcome. Studies involving pregnant women were excluded.

**Results:**

A total of 37 studies evaluating seven different HCL systems (CamAPS Fx, Control IQ, DBLG1, iLet BP, MiniMed 670G, MiniMed 780G, and Omnipod 5) were included. In studies with a mean age < 18 years, mean TIR was 64.1% (95% CI: 61‐67.2), ranging from 59.3% (95% CI: 49.6‐69.1) with MiniMed 780G to 68% (95% CI: 65.8‐70.3) with Control IQ, and end‐of‐study HbA1c was 7.4% (95% CI: 7‐7.7), ranging from 6.7% (95% CI: 6.6‐6.9) with CamAPS Fx to 7.9% (95% CI: 6.9‐9) with MiniMed 780G. In studies with a mean age ≥ 18 years, mean TIR was 70.8% (95% CI: 68.6‐73), ranging from 63.1% (95% CI: 59.4‐66.8) with Omnipod 5 to 74.4% (95% CI: 69.7‐79.1) with MiniMed 780G, and end‐of‐study HbA1c was 7.1% (95% CI: 7‐7.3), ranging from 7.0% (95% CI: 6.9‐7.1) with Control IQ to 7.2% (95% CI: 7‐7.5) with MiniMed 670G.

**Conclusions:**

In RCTs, commercial HCL systems show different achievements of CGM metrics and HbA1c in people with type 1 diabetes.

## 1 INTRODUCTION

In recent years, hybrid closed loop (HCL) systems, providing algorithm‐driven, glucose‐responsive insulin delivery, have revolutionised diabetes care with unprecedented improvements in glucose control and hypoglycaemia risk.^1^, ^2^, ^3^ A recent meta‐analysis of randomised controlled trials (RCTs) conducted in children, adolescents, and adults with type 1 diabetes found a statistically significant increase in time in range (TIR; mean difference [MD] 10.87%; 95% confidence interval [CI],: 8.31–13.44) and reductions in HbA1c (MD −0.35%; 95% CI: −0.43 to −0.27), time above range (TAR; MD −1.18%; 95% CI: −1.63 to −0.73), time below range (TBR; MD −1.18%; 95% CI: −1.63 to −0.73), and coefficient of variation of glucose (CV; MD −1.11%; 95% CI: −2.02 to −0.20) with HCL systems as compared with other insulin‐based treatments (i.e., multiple daily injections [MDI], continuous subcutaneous insulin infusion [CSII], sensor‐augmented pump therapy [SAPT], and SAPT with low‐glucose [LGS] or predictive low‐glucose [PLGS] suspend features).^4^ In light of such findings, international guidelines recommend HCL systems as the preferred insulin delivery method in youth and adults with type 1 diabetes.^5^

Seven commercial HCL systems are most commonly used worldwide, namely CamAPS Fx (CamDiab Ltd., Cambridge, UK), DBLG1 (Diabeloop, Grenoble, France), iLet Bionic Pancreas (iLet BP) (Beta Bionics, Irvine, CA, USA), MiniMed 670G (Medtronic, Northridge, CA, USA), MiniMed 780G (Medtronic, Northridge, CA, USA), T:slim X2 with Control‐IQ technology (Control IQ) (Tandem Diabetes Care, San Diego, CA, USA), and Omnipod 5 (Insulet Corporation, Acton, MA, USA), each of them showing peculiarities in insulin‐dosing strategies and modifiable settings, possibly resulting in different glycaemic outcomes.^6^, ^7^

The aim of this meta‐analysis is to assess the performance of different HCL systems in RCTs on youth and adults with type 1 diabetes.

## 2 METHODS

The study protocol was registered prior to conduct (PROSPERO 2025 CRD420251074233).

### 2.1 Data sources

We searched MEDLINE (via Ovid), PubMed, Web of Science, and CENTRAL from inception to 18 April 2025 (Appendix S1) and performed hand‐searching of conference proceedings to find all relevant studies outside the databases.

### 2.2 Ethics approval

Analyses were performed on data extracted from published papers. Patient consent for publication was not required.

### 2.3 Study selection

We included RCTs enrolling children, adolescents, and adults with type 1 diabetes, evaluating commercially available HCL systems against other types of subcutaneous intensive insulin therapy, with a duration of intervention equal to or longer than 2 weeks, and reporting 24‐h TIR as an outcome. Trials conducted in special populations of persons with diabetes including pregnant women, people with kidney or liver failure, or with highly unstable diabetes (defined as a form of type 1 diabetes in which blood glucose levels fluctuate unpredictably, swinging between dangerously high and low levels^8^), and hospitalized people, or evaluating HCL systems during experimentally induced stress challenges (physical exercise, gastronomic meals, etc.) were excluded as insulin therapy in these subgroups and/or situations may not reflect the usual practice.

The primary outcome of the meta‐analysis was 24‐h TIR achieved at trial end (mean, 95% CI). Secondary outcomes were 24‐h time spent in the 70–140 mg/dL glucose range (time in tight range, TITR), 24‐h TBR, 24‐h time below 54 mg/dL (TBR2), 24‐h TAR, 24‐h mean sensor glucose, CV, HbA1c, prevalence of severe hypoglycaemia (SH), and diabetic ketoacidosis (DKA). All the above‐mentioned outcomes were analysed only in subjects who had been assigned to HCL treatment arms, irrespective of control groups.

### 2.4 Data extraction

Four reviewers (IC, LDG, MC, SDM) independently evaluated article titles and abstracts based on inclusion and exclusion criteria. Disagreements were settled by discussion. The following information was collected from the included studies: study characteristics (design, duration, year of publication, and sample size), participants' characteristics (age, sex, HbA1c at baseline [%], and disease duration), HCL system under evaluation, comparator(s), TIR (%), TITR (%), TBR (%), TBR2 (%), mean sensor glucose (mg/dL), TAR (%), CV (%), HbA1c (%), and proportion of patients having SH or DKA. Study investigators were contacted in case of missing data. If the mean was missing, it was calculated by dividing the sum of the median, first quartile, and third quartile by 3.^9^ If the standard deviation (SD) was missing, it was calculated by multiplying the standard error (SE) by the square root of the sample size.^9^ When the SD was neither available nor calculable, it was imputed based on the higher value within each group.^10^ Disagreements in data extraction were settled by debate.

### 2.5 Risk of bias assessment

Risk of bias was assessed independently by two reviewers (IC, LDG) through the Cochrane Collaboration's tool (RoB 2, version 22 August 2019; RoB 2 crossover, version 18 March 2021) evaluating the following domains: randomization process; bias arising from period and carryover effects; deviations from intended intervention; missing outcome data; measurement of the outcome; selection of the reported result; overall bias. Each domain was deemed at low, with some concerns or high risk of bias. Any differences in assessment were resolved by consensus.

### 2.6 Statistical analysis

The systematic review and the meta‐analysis were performed in line with recommendations from the Cochrane Collaboration and the Preferred Reporting Items for Systematic Reviews and Meta‐Analysis (PRISMA) statement guidelines. In the descriptive analyses, continuous variables were summarised as mean ± SD or as median with interquartile range (IQR) in case of non‐normal distribution, and categorical variables as absolute counts and/or percentages. In the meta‐analysis, continuous outcomes were reported as means with 95% CIs, and binary outcomes as proportions with the corresponding 95% CIs. Ninety‐five percent prediction intervals (95% PI) were also computed to show the range within which the relative treatment effect of a future study is expected to fall, accounting for between‐study heterogeneity. In addition, heterogeneity was evaluated with Cochran's *Q* test and *I* ^2^: *p* values <0.05 and *I* ^2^ > 25% were considered significant for heterogeneity. Given the substantial heterogeneity observed across studies, all meta‐analyses were conducted using random‐effects models.^11^ The presence of publication bias was evaluated using funnel plots and Egger's tests. Prespecified subgroup analyses were conducted by mean age of participants (<18 years vs. ≥18 years) and mean HbA1c at baseline, given the potential influence of these factors on TIR. Meta‐regression analyses were also performed to explore the relationship between TIR and baseline HbA1c levels. Adjusted forest plots were subsequently generated, presenting TIR estimates for specific baseline HbA1c values. The Instrument for assessing the Credibility of Effect Modification Analyses (ICEMAN) tool was used to assess credibility of subgroup effects for interaction *p*‐values <0.1. All analyses were performed using Rstudio and R package meta.^13^

**FIGURE 1:**
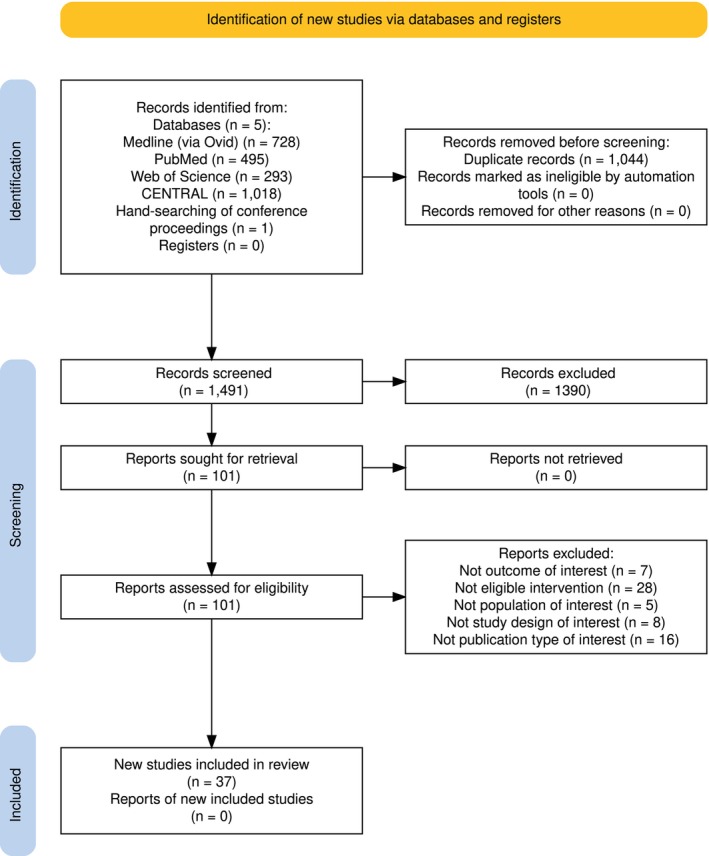
PRISMA flow diagram of study search strategy.^12^

## 3 RESULTS

A total of 37 studies^14^, ^15^, ^16^, ^17^, ^18^, ^19^, ^20^, ^21^, ^22^, ^23^, ^24^, ^25^, ^26^, ^27^, ^28^, ^29^, ^30^, ^31^, ^32^, ^33^, ^34^, ^35^, ^36^, ^37^, ^38^, ^39^, ^40^, ^41^, ^42^, ^43^, ^44^, ^45^, ^46^, ^47^, ^48^, ^49^, ^50^, ^51^, ^52^ were included in the meta‐analysis (Figure 1), of which one evaluated two different HCL systems (MiniMed 670G and MiniMed 780G) one against each other and 36 evaluated HCL systems against other types of subcutaneous intensive insulin therapy, for a total of 38 data sources and 2228 subjects treated with HCL insulin delivery. In detail, CamAPS Fx was evaluated in 5 studies (216 subjects), Control IQ in 7 studies (458 subjects), DBLG1 in 3 studies (153 subjects), iLet BP in 2 studies (219 subjects), MiniMed 670G in 9 studies (491 subjects), MiniMed 780G in 10 studies (434 subjects), and Omnipod 5 in 2 studies (257 subjects). The main characteristics of the included studies and participants are provided in Appendix S2. The duration of intervention across the included studies ranged from 4 to 104 weeks. All but two studies^42^, ^49^ included more than 10 individuals treated with HCL therapy. The overall risk of bias for the main outcome was deemed low for 29 trials, of some concern for 7 trials, and high for 1 trial (Appendix S3). Funnel plots and Egger's tests were used to explore publication bias and potential small‐study effects. No clear evidence of such bias was found for most outcomes. However, for specific outcomes, most notably TIR, signs of funnel plot asymmetry and significant Egger's test results were observed (full analyses and plots are provided in Appendix S4).

**FIGURE 2:**
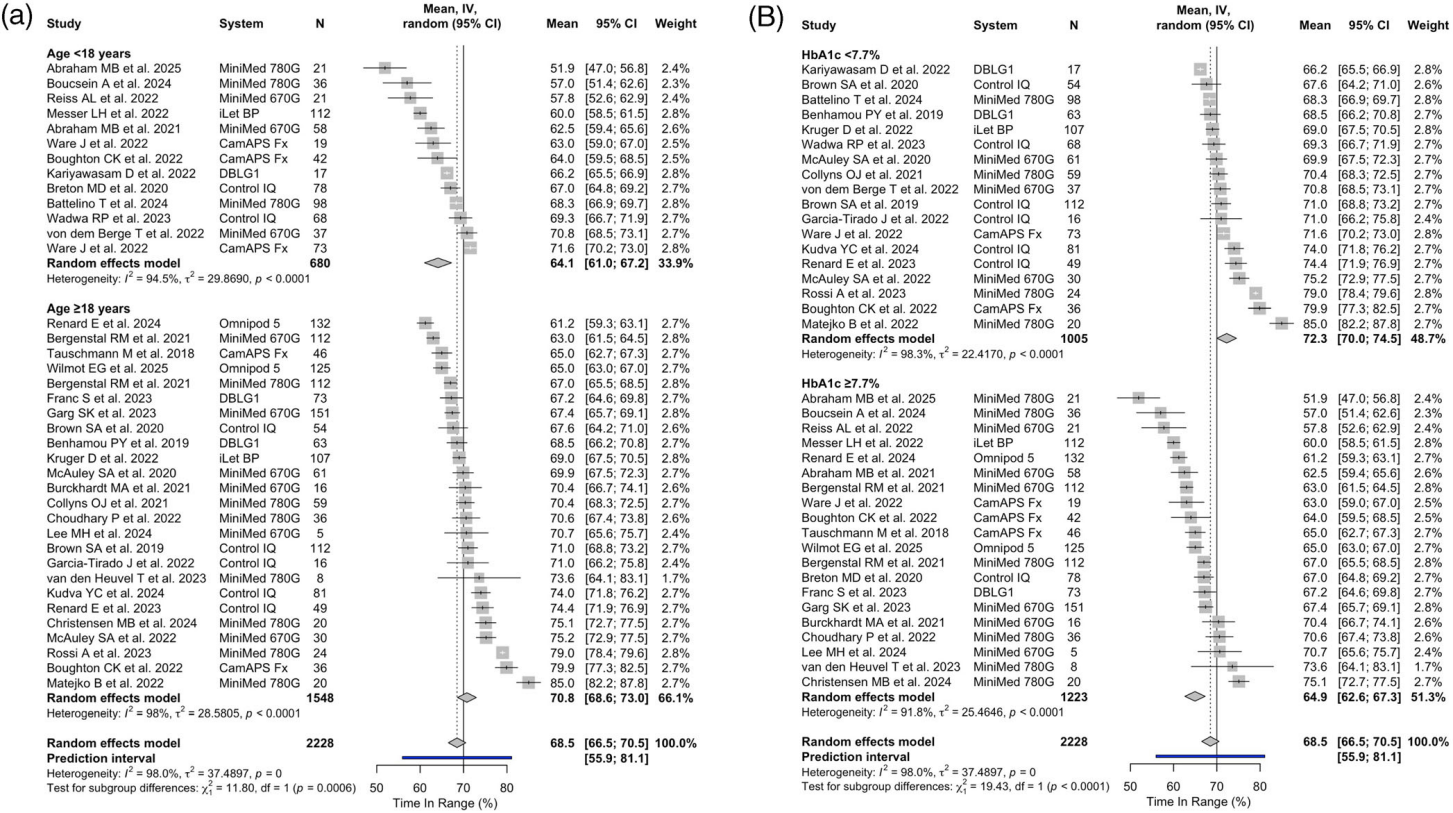
Time In Range across systems, stratified by age and baseline HbA1c, achieved at trial end. Forest plots summarising the effects of different hybrid closed loop systems on time in range (TIR, 70–180 mg/dL), with subgroup analyses by age group (<18 years vs. ≥18 years) (A) and baseline HbA1c (<7.7% vs. ≥7.7%) (B). Each plot reports the mean achieved TIR with 95% confidence intervals for each trial, as well as the overall random‐effects estimate and corresponding prediction interval. Measures of heterogeneity and subgroup differences are reported at the bottom of each panel. The dashed vertical line represents the overall effect estimate, while the solid vertical line indicates the reference TIR threshold of 70%, as suggested by international consensus and clinical guidelines. CI, confidence interval; IV, inverse variance.

Thirteen studies (680 subjects) were conducted in participants with mean age < 18 years and 24 (25 data sources, 1548 subjects) in participants with mean age ≥ 18 years. Eighteen studies had mean baseline HbA1c <7.7% and 19 (20 data sources) ≥7.7%. Mean TIR at trial end achieved with HCL systems ranged from 51.9% to 71.6% in studies with mean participant age < 18 years and from 61.2% to 85% in those with mean age ≥ 18 years. The random‐effects meta‐analytic mean of TIR was 64.1% (95% CI: 61.0–67.2; 95% PI: 51.7–76.5) and 70.8% (95% CI: 68.6–73.0; 95% PI: 59.5–82.0), respectively (Figure 2A). A mean TIR >70% was achieved in 2 out of 13 (15.4%) studies with mean age < 18 years and in 14 out of 25 (56%) studies with mean age ≥ 18 years, respectively. Mean TIR with HCL systems was higher in studies with lower baseline HbA1c (72.3%, 95% CI: 70.0–74.5) than in studies with higher baseline HbA1c (64.9%, 95% CI: 62.6–67.3) (Figure 2B). In a sensitivity analysis excluding the two studies with fewer than 10 participants (Lee et al.^49^ with MiniMed 670G and van den Heuvel et al.^42^ with MiniMed 780G, both conducted in populations with mean age ≥ 18 years), results remained consistent with the main analysis (Appendix S7).

TITR was reported in 20 out of 37 included studies (21 data sources). In studies with a mean participant age < 18 years, TITR achieved at trial end ranged from 36.3% to 55%, with a pooled estimate from random‐effects meta‐analysis of 41.1% (95% CI: 36.5–45.7); whereas, in those with mean age ≥ 18 years, TITR ranged from 30.9% to 48%, with a pooled estimate of 44.4% (95% CI: 41.7–47.2). None of the 8 studies with a mean age < 18 years achieved a mean TITR >50%; on the other hand, 2 of the 12 studies (13 data sources) with mean age ≥ 18 years achieved a mean TITR >50%.

TBR was reported in all included studies. In studies with a mean participant age < 18 years, TBR at trial end ranged from 1.3% to 12.4%, with a pooled estimate from random‐effects meta‐analysis of 3.8% (95% CI: 2.3–5.3); whereas, in those with mean age ≥ 18 years, TBR ranged from 1.2% to 3.5%, with a pooled estimate of 2.0% (95% CI: 1.8–2.2). A mean TBR < 4% was achieved in 10 out of 13 (76.9%) studies with mean age < 18 years and in all studies with mean age ≥ 18 years, respectively.

Tables 1 and 2 summarise the pooled estimates of CGM‐derived glucose metrics and HbA1c levels by type of HCL system achieved in studies with mean age of participants <18 years and ≥18 years, respectively (full analyses are reported in Appendix S4). In studies with mean age < 18 years (Table 1), mean TIR ranged from 59.3% (95% CI: 49.6‐69.1) with MiniMed 780G to 68% (95% CI: 65.8‐70.3) with Control IQ, TITR from 38.1% (95% CI: 27.9‐48.2) with MiniMed 780G to 45.9% (95% CI: 42‐49.9) with Control IQ, TBR from 1.9% (95% CI: 1.8‐2.0) with iLet BP (one single study) to 9.1% (95% CI: 4.4‐13.8) with CamAPS Fx, CV from 36.4% (95% CI: 34.8‐38) with DBLG1 (one single study) to 45.1% (95% CI: 40.4‐49.7) with CamAPS Fx, and HbA1c from 6.7% (95% CI: 6.6‐6.9) with CamAPS Fx to 7.9% (95% CI: 6.9‐9.0) with MiniMed 780G. Omnipod 5 was not included in Table 1 as none of the two included studies with this system had a mean age < 18 years. Moreover, TITR was not available for CamAPS Fx, and end‐of‐trial HbA1c was not available for DBLG1. In studies with mean age ≥ 18 years (Table 2), mean TIR ranged from 63.1% (95% CI: 59.4‐66.8) with Omnipod 5 to 74.4% (95% CI: 69.7‐79.1) with MiniMed 780G, TITR from 36.3% (95% CI: 34.5‐38.1) with Omnipod 5 (one single study) to 49.5% (95% CI: 38.7‐60.3) with MiniMed 780G, TBR from 1.6% (95% CI: 1.1‐2.2) with DBLG1 to 2.2% (95% CI: 1.4‐3.1) with CamAPS Fx, CV from 31% (95% CI: 30‐32) with DBLG1 (one single study) to 36.3% (95% CI: 35.6‐36) with Omnipod 5, and HbA1c from 7.0% (95% CI: 6.9‐7.1) with Control IQ to 7.2% (95% CI: 7‐7.5) with MiniMed 670G. Importantly, TITR was not available for CamAPS Fx.

**TABLE 1:** Sensor‐derived metrics and HbA1c in RCTs on people with type 1 diabetes with a mean age < 18 years treated with hybrid closed‐loop systems.

	CamAPS FX	Control IQ	DBLG1	iLet BP	MiniMed 670G	MiniMed 780G	Omnipod 5	Overall
TIR (70–180 mg/dL)	66.5 (60.9; 72.2)	68 (65.8; 70.3)	66.2 (65.5; 66.9)^a^	60.0 (58.5; 61.5)^a^	63.9 (56.5; 71.4)	59.3 (49.6; 69.1)	NA	64.1 (61; 67.2)
TITR (70–140 mg/dL)	NA	45.9 (42; 49.9)	40.5 (39.7; 41.2)^a^	35 (33.7; 36.3)^a^	40.4 (37.7; 43.1)	38.1 (27.9; 48.2)	NA	40.3 (36; 44.6)
TBR (<70 mg/dL)	9.1 (4.4; 13.8)	2.3 (0.9; 3.7)	2.4 (1.8; 2.9)^a^	1.9 (1.8; 2.0)^a^	3.2 (2.4; 4)	2.6 (0.5; 4.6)	NA	3.8 (2.3; 5.3)
TBR2 (<54 mg/dL)	2.4 (0.8; 4)	0.4 (0; 0.8)	0.5 (0.3; 0.6)^a^	0.4 (0.3; 0.4)^a^	0.6 (0.3; 0.9)	0.6 (0.2; 0.9)	NA	0.8 (0.4; 1.3)
TAR (>180 mg/dL)	23.1 (21.9; 24.3)	29.6 (26.6; 32.5)	31.6 (30.5; 32.7)^a^	38.0 (36.5; 39.5)^a^	32.6 (25.3; 39.9)	38.1 (26.5; 49.7)	NA	31.4 (27.4; 35.4)
Mean glucose (mg/dL)	144.5 (140.9; 148)	158.6 (151.8; 165.5)	158.9 (152.9; 164.9)^a^	172 (169.4; 174.6)^a^	163 (150.2; 175.7)	172.4 (149.2; 195.6)	NA	160 (152.3; 167.8)
CV (%)	45.1 (40.4; 49.7)	38.4 (37.5; 39.4)	36.4 (34.8; 38)^a^	38.0 (37.3; 38.7)^a^	36.4 (33.3; 39.6)	39.4 (37.1; 41.7)	NA	39.4 (37.4; 41.5)
HbA1c (%)	6.7 (6.6; 6.9)	7 (6.8; 7.2)^a^	NA	7.5 (7.4; 7.6)^a^	7.5 (6.8; 8.2)	7.9 (6.9; 9.0)	NA	7.4 (7; 7.7)

*Note*: Summary of pooled estimates of sensor‐derived metrics and HbA1c in individuals with type 1 diabetes treated with different hybrid closed‐loop systems, based on study populations with a mean age < 18 years.

Abbreviations: CV, coefficient of variation; NA, not available; TAR, time above range; TBR, time below range; TBR2, time below range level 2; TIR, time in range; TITR, time in target range.

^a^ One single study.

**TABLE 2:** Sensor‐derived metrics and HbA1c in RCTs on people with type 1 diabetes with a mean age ≥ 18 years treated with hybrid closed‐loop systems.

	CamAPS FX	Control IQ	DBLG1	iLet BP	MiniMed 670G	MiniMed 780G	Omnipod 5	Overall
TIR (70‐180 mg/dL)	72.4 (57.8; 87)	71.8 (69.4; 74.2)	67.9 (66.2; 69.6)	69 (67.5; 70.5)^a^	69.3 (65.8; 72.7)	74.4 (69.7; 79.1)	63.1 (59.4; 66.8)	70.8 (68.6; 73)
TITR (70–140 mg/dL)	NA	46 (42.4; 49.7)	39.3 (37.3; 41.3)^a^	42 (40.7; 43.3)^a^	44.4 (41.0; 47.8)	49.5 (38.7; 60.3)	36.3 (34.5; 38.1)^a^	44.4 (41.7; 47.2)
TBR (<70 mg/dL)	2.2 (1.4; 3.1)	2.0 (1.5; 2.5)	1.6 (1.1; 2.2)	1.9 (1.7; 2.2)^a^	2.1 (1.5; 2.6)	2.1 (1.9; 2.2)	1.9 (0.5; 3.2)	2 (1.8; 2.2)
TBR2 (<54 mg/dL)	0.2 (0.2; 0.2)^a^	0.4 (0.2; 0.5)	NA	0.3 (0.3; 0.4)^a^	0.4 (0.2; 0.5)	0.5 (0.4; 0.5)	0.2 (0.2; 0.3)^a^	0.4 (0.3; 0.4)
TAR (>180 mg/dL)	24.7 (10.3; 39.1)	26.1 (23.2; 28.9)	30.3 (28.4; 32.2)	28 (26.3; 29.7)^a^	28.5 (25.3; 31.6)	22.7 (17.8; 27.7)	34.8 (29.3; 40.3)	26.8 (24.5; 29.1)
Mean glucose (mg/dL)	150.3 (130.9; 169.7)	153.7 (149.6; 157.9)	156.6 (153; 160.2)^a^	157 (154.7; 159.3)^a^	157 (151.4; 162.6)	147.6 (140.9; 154.4)	174 (170.6; 177.4)^a^	153.5 (149.8; 157.3)
CV (%)	36.3 (28.9; 43.6)	34.1 (33.2; 35)	31 (30; 32)^a^	34 (33.2; 34.8)^a^	34.5 (32.6; 36.5)	34.6 (32.8; 36.5)	36.3 (35.6; 37)^a^	34.5 (33.6; 35.5)
HbA1c (%)	7.1 (6.4; 7.7)	7.0 (6.9; 7.1)	7.3 (7.1; 7.5)^a^	7.1 (7.0; 7.2)^a^	7.2 (7; 7.5)	7.1 (6.8; 7.4)	7.2 (7.1; 7.3)	7.1 (7; 7.3)

*Note*: Summary of pooled estimates of sensor‐derived metrics and HbA1c in individuals with type 1 diabetes treated with different hybrid closed‐loop systems, based on study populations with a mean age ≥ 18 years.

Abbreviations: CV, coefficient of variation; NA, not available; TAR, time above range; TBR, time below range; TBR2, time below range level 2; TIR, time in range; TITR, time in target range.

^a^ One single study.

**FIGURE 3:**
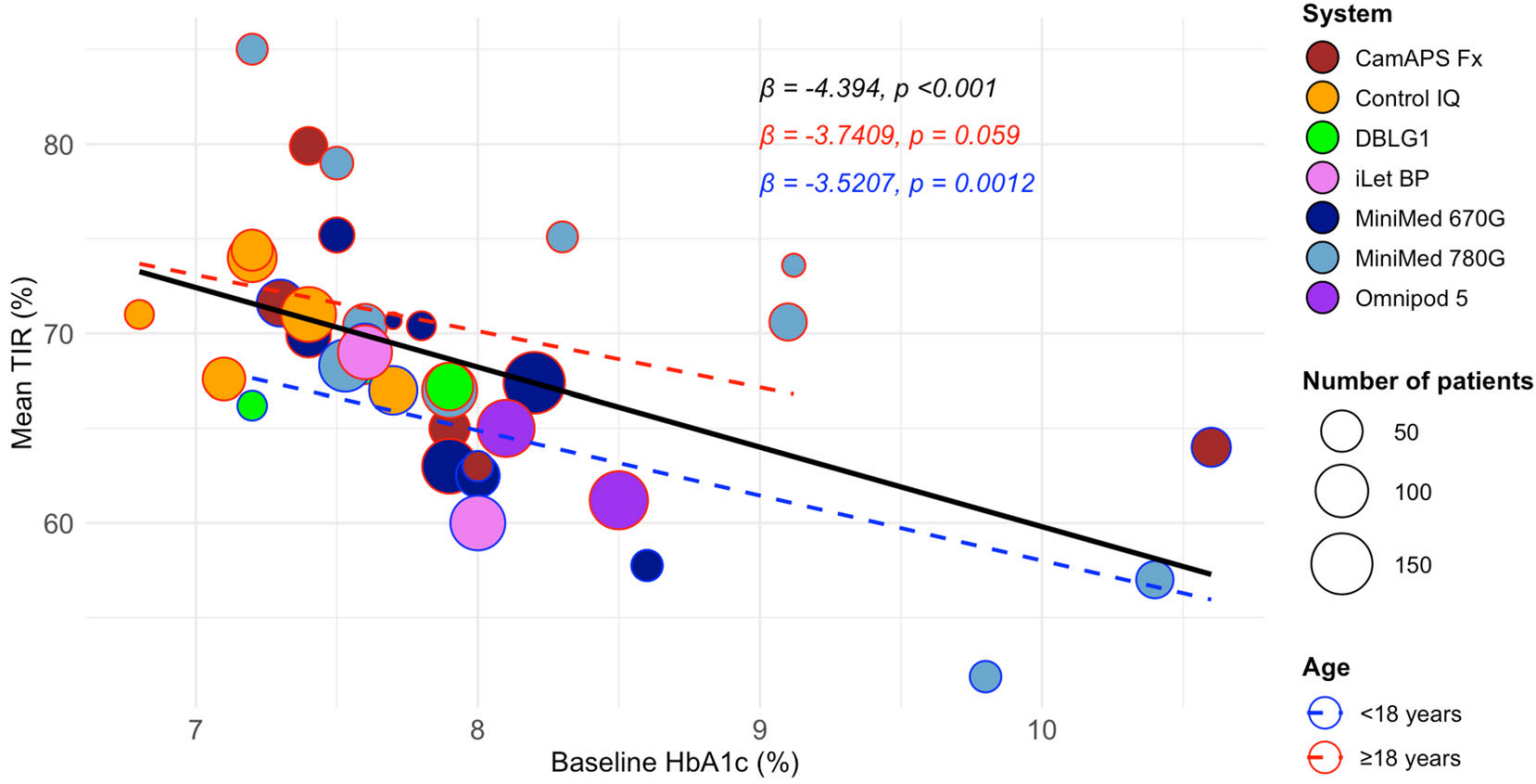
Meta‐regression analyses between baseline HbA1c and mean time in range (TIR) at trial end.

At meta‐regression analyses, an inverse relationship was observed between baseline HbA1c and end‐of‐trial TIR in the included studies considered in their entirety, and in the two age groups (Figure 3). In spite of the general trend, it should be noted that, in studies with mean age ≥ 18 years, MiniMed 780G, differently from other systems, achieved TIR values >70% even starting from a baseline HbA1c of >8%. By contrast, in studies with mean age < 18 years, no system reached TIR values >70% when baseline HbA1c exceeded 8%.

To account for between‐study differences in baseline glycaemic status, meta‐regression‐adjusted forest plots for TIR were generated for baseline HbA1c levels of 7.5%, 8.0%, and 8.5% (Appendix S6). As an example, in studies enrolling subjects with mean age < 18 years, the pooled estimate of TIR values, adjusted for a baseline HbA1c of 7.5%, was 66.8% (95% CI: 64.6%–69.0%; 95% PI: 58.4%–75.2%), with the system‐specific estimates being 70.2% (95% CI: 64.7%–75.6%) for CamAPS Fx, 68.5% (95% CI: 66.7%–70.4%) for Control IQ, 65.1% (95% CI: 64.4%–65.8%) for DBLG1, 61.8% (95% CI: 60.3%–63.2%) for iLet BP, 65.8% (95% CI: 60.6%–71.0%) for MiniMed 670G, and 65.5% (95% CI: 60.4%–70.6%) for MiniMed 780G (Appendix S6.1). By contrast, in studies enrolling subjects with mean age ≥ 18 years, the pooled estimate of TIR values, adjusted for a baseline of HbA1c of 7.5%, was 71.7% (95% CI: 69.7%–73.7%; 95% PI: 61.4%–82.0%), with the system‐specific estimates being 73.0% (95% CI: 60.2%–85.8%) for CamAPS Fx, 70.5% (95% CI: 67.9%–73.2%) for Control IQ, 68.8% (95% CI: 67.1%–70.5%) for DBLG1, 69.4% (95% CI: 67.9%–70.9%) for iLet BP, 70.2% (95% CI: 67.2%–73.3%) for MiniMed 670G, 76.4% (95% CI: 72.3%–80.5%) for MiniMed 780G, and 66.1% (95% CI: 63.8%–68.3%) for Omnipod 5 (Appendix S6.2).

The prevalence of SH and DKA was very low across the included studies. Notably, no DKA events were reported in studies with a mean age < 18 years (Appendix S6).

## 4 DISCUSSION

HCL systems have proven safe and effective in improving glycaemic outcomes in youth and adults with type 1 diabetes. Our meta‐analysis found a mean TIR of 64.1% and 70.8% in studies with a mean age of participants <18 years and ≥18 years, respectively, with 15.4% and 56% of studies in the two age groups reporting a mean TIR >70%. In line with these results, in a meta‐analysis published in 2021, Aiello et al. found a mean TIR of 67.5% in trials conducted in paediatric age and of 72.5% in trials on adults, albeit with a higher proportion of them (51.7% and 73.5%, respectively) reaching the TIR target of >70%.^53^ However, the analysis included any study with a duration equal to or longer than 1 day, comprising single‐arm early feasibility studies. By contrast, we decided to focus only on RCTs with a duration of intervention ≥2 weeks to obtain more reliable effect estimates and glucose metrics computed over at least 14 days of CGM data, as recommended by international consensus documents.^54^, ^55^ Additionally, as compared with Aiello et al., our meta‐analysis also included studies published in the years 2021–2025, comprising two RCTs evaluating iLet BP and two RCTs evaluating Omnipod 5.

It is well established that paediatric populations, and adolescents in particular, face the greatest challenges in achieving glycaemic targets, regardless of the treatment strategy.^56^ While we acknowledge that a cohort with a mean participant age < 18 years does not necessarily represent a purely paediatric population, our findings align with this observation. Nevertheless, HCL systems have consistently proven to be the most effective approach in this age group.^57^, ^58^ Mean TIR achieved at trial end was reported to be higher (72.3%) in studies with lower baseline HbA1c as compared with their counterparts (64.9%). This is not surprising as the initial glycaemic status is a well‐established predictor of TIR achievements with HCL systems in real‐world observations.^59^, ^60^ On the other hand, a higher baseline HbA1c is associated with greater improvement in hyperglycaemia measures following HCL initiation,^4^, ^61^ and suboptimal glycaemic status is a widely accepted indication for recommending treatment with HCL systems.^62^

Mean TBR was within the 4% threshold in the great majority (about 90%) of the included studies, thereby confirming that glucose lowering with HCL technology occurs without an increased risk of hypoglycaemia. Consistently with this finding, the prevalence of SH was also low across the included studies.

Our meta‐analysis is the first to compute pooled estimates by type of HCL system for several CGM‐derived glucose metrics and end‐of‐trial HbA1c. Importantly, we decided to evaluate only subjects assigned to HCL arms in order to highlight the ‘absolute’ effect of HCL therapy on glycaemic outcomes (i.e., an effect not influenced by the performance of control treatments) and the potential achievement of recommended targets. We showed that individuals treated with different HCL systems achieve varying TIR values, ranging from 59.3% to 68% in studies with a mean participant age < 18 years, and from 63.1% to 74.4% in those with a mean age ≥ 18 years, with Control‐IQ and MiniMed 780G being associated with the highest TIR values in the <18 years and ≥18 years cohorts, respectively. Despite achieving the highest TIR in the ≥18 years cohort, MiniMed 780G showed the lowest TIR in the <18 years cohort. However, two of the three studies with MiniMed 780G in the latter cohort included patients with very high baseline HbA1c levels (>9.5%; Figure 3), which may dampen the system's performance in this age group, as low HbA1c at HCL start is an established factor contributing to better TIR, as shown in the meta‐regression analysis.

Since end‐of‐study TIR was inversely associated with baseline HbA1c in meta‐regression analyses, adjusted TIR values for specific baseline HbA1c levels were calculated. After adjustment for baseline HbA1c, MiniMed 780G remained the leading system in RCTs with mean age ≥ 18 years; in contrast, this was not observed for Control IQ, as CamAPS Fx emerged as the system with the highest TIR in the cohorts with <18 years of age when the same baseline HbA1c was assumed across studies. Of note, in the latter cohorts, all the systems but iLet BP achieved a mean TIR ≥65% at a baseline HbA1c of 7.5% (Appendix S6.1.1). Mean TBR was within the 4% threshold with all HCL systems except for CamAPS Fx (9.1%) in RCTs with mean age < 18 years. However, the high TBR observed with CamAPS Fx can be reasonably attributed to the unusually elevated baseline level in two studies^25^, ^36^ and, at least in one of them, to the overestimation of hypoglycaemic exposure with the FreeStyle Libre Pro sensor.^36^ The CV averaged 39.4% (95% CI: 37.4‐41.5) in studies with mean age < 18 years and 34.5% (95% CI: 33.6‐35.5) in those with mean age ≥ 18 years, with DBLG1 and CamAPS Fx being associated with the lowest (<18 years: 36.4%; ≥18 years: 31%) and highest (<18 years: 45.1%; ≥18 years: 36.3%) glycaemic variability, respectively. As expected based on previous observations,^63^ CV results aligned directly with those of the TBR, that is, systems reaching a lower TBR also showed a lower CV and vice versa. Interestingly, recent evidence has questioned the value of CV as a metric for device performance and hypoglycaemia risk, as well as a therapeutic target in HCL users. Since CV reflects the ratio between SD and mean glucose levels, improvements in both (i.e., a reduction in SD and a decrease in mean glucose, which are commonly observed with HCL insulin delivery and considered positive outcomes) may offset each other, resulting in only subtle changes in CV.^64^

Our analysis has some limitations that should be acknowledged. First, since the included RCTs used different brands of sensors, any difference in CGM‐derived glucose metrics among the investigated systems should be interpreted with caution. In this scenario, while end‐of‐trial HbA1c would represent a more reliable outcome measure,^65^ it does not capture the full spectrum of hypoglycaemic and hyperglycaemic excursions and was not available for DBLG1 in studies with mean age < 18 years. Moreover, asymmetry in funnel plots and significant Egger's tests were observed for some outcomes. In the setting of a meta‐analysis, such patterns may indicate small‐study effects, selective reporting, or contextual differences in study populations. Although publication bias in the traditional sense may not fully apply here, these findings suggest that the overall effect estimates, particularly for TIR, could be influenced by the overrepresentation of smaller studies with favourable results. However, in a sensitivity analysis excluding the two trials with fewer than 10 participants,^42^, ^49^ the results for TIR and the relevant subgroup analyses (by age group and by HCL system) remained consistent with the main analysis, supporting the robustness of our findings. Another limitation is that most included studies enrolled participants with heterogeneous baseline insulin regimens (MDI, CSII, SAPT, or HCL), which prevented us from assessing whether prior insulin therapy may have influenced the observed treatment effects. Finally, the applicability of our results cannot be generalised to people with type 2 diabetes and “special” populations with diabetes, including pregnant women, individuals with end‐stage renal disease, and hospitalised patients, as they were excluded from the search criteria.

Factors to consider when selecting an HCL system include pump and sensor features, algorithm specifications, regulatory indications, and supporting evidence on clinical outcomes from both RCTs and real‐world studies.^66^ In line with this approach, we believe that the results of clinical trials, including CGM metric achievements, should inform the decision‐making process, guiding the selection of specific technologies based on individual glycaemic goals. Importantly, this perspective is also consistent with the recommendations of the American Diabetes Association, which emphasise that the choice of an HCL system should be based on the patient's clinical needs, circumstances, and preferences.^5^

## 5 CONCLUSION

In RCTs conducted in individuals with type 1 diabetes, different HCL systems achieve varying CGM metrics, including TIR values ranging from 59.3% to 68% in studies with a mean participant age < 18 years, and from 63.1% to 74.4% in those with a mean age ≥ 18 years, as well as different HbA1c levels. We hope that our results can be useful for clinicians to guide people with diabetes in the HCL system selection process, with the objective of obtaining optimal glycaemic outcomes.

## AUTHOR CONTRIBUTORS

SDM, LDG, and FG contributed to the study conception and design. SDM, MC, LDG, and IC evaluated the articles for eligibility and collected the data. LDG performed the statistical analysis. IC and LDG assessed the risk of bias. LDG created the figures. LDG and MC created the tables. SDM, LDG, and IC wrote the first draft of the manuscript. FG, LL, PT, AN, and SP revised the manuscript and contributed to the discussion. All authors read and approved the final manuscript. FG is the guarantor of the work and, as such, has full access to all the data in the study and takes responsibility for data integrity and accuracy of data analysis.

## CONFLICT OF INTEREST STATEMENT

FG: Eli Lilly, Roche Diabetes Care (grants); Eli Lilly, Novo Nordisk (consulting fees); AstraZeneca, Boehringer‐Ingelheim, Eli Lilly, Lifescan, Merck Sharp & Dohme, Medtronic, Novo Nordisk, Roche Diabetes Care, Sanofi Aventis (support for attending meetings or travels); AstraZeneca, Boehringer‐Ingelheim, Eli Lilly, Lifescan, Merck Sharp & Dohme, Medimmune, Medtronic, Novo Nordisk, Roche Diabetes Care, Sanofi Aventis (participation on Advisory Boards); EASD/EFSD, Società Italiana di Endocrinologia (SIE), Fo.Ri.SIE (unpaid leadership); AstraZeneca, Eli Lilly, Novo Nordisk, Sanofi Aventis (support for medical writing and statistical analysis). SDM: Ascensia Diabetes Care, MOVI SpA, Roche Diabetes Care (honoraria); Ascensia Diabetes Care, MOVI SpA, Menarini Diagnostics, Roche Diabetes Care, Sanofi Aventis (participation on Advisory Boards). LDG: Eli Lilly, Roche Diabetes Care, MOVI SpA, Sanofi, Theras (honoraria); Abbott, Eli Lilly, Lusofarmaco, Novo Nordisk, Sanofi (support for attending meetings or travels); Eli Lilly (participation on Advisory Boards). IC: Eli Lilly, Novo Nordisk, Guidotti SpA, AstraZeneca (honoraria); Eli Lilly, Novo Nordisk, Abbott (support for attending meetings or travels). MC: Eli Lilly, Lusofarmaco, Novo Nordisk, Sanofi (support for attending meetings or travels). PT: nothing to disclose. AN: AstraZeneca, Novo Nordisk, and Sanofi Aventis (honoraria). SP: AstraZeneca, Eli Lilly, Novo Nordisk, Sanofi Aventis (honoraria). LL: Abbott, AstraZeneca, Boehringer‐Ingelheim, Eli Lilly, Merck Sharp & Dohme, Medtronic, Menarini, MOVI SpA, Mundipharma, Novo Nordisk, Roche Diabetes Care, Sanofi Aventis, Terumo (honoraria); Abbott, AstraZeneca, Boehringer‐Ingelheim, Eli Lilly Italia, Medtronic, MOVI SpA, Novo Nordisk, Roche Diabetes Care, Sanofi Aventis, Terumo (participation on Advisory Boards).

## Supporting information


**Appendix S1.** Supporting information.

## Data Availability

All data relevant to the study are included in the article or uploaded as supplementary information. Statistical code and data set: available on reasonable request from Prof. Francesco Giorgino (francesco.giorgino@uniba.it).
