# Supplementary material for: Glycaemic control and variability with different commercially available hybrid closed loop systems in people with type 1 diabetes: A systematic review and meta‐analysis of randomized controlled trials
Source: Diabetes Obes Metab. 2025 Sep 29;28(Suppl 1):3–12. doi: 10.1111/dom.70150 (PMC12805927; doi:10.1111/dom.70150)
Supplement: Supplementary file 1 — Appendix S1. Supporting information. [file DOM-28-3-s001.docx]

**Supplementary Appendix**

Glycemic control and variability with different commercially-available hybrid closed loop systems in people with type 1 diabetes: a systematic review and meta-analysis

**Contents**

[**Supplement 1. Search strategy 3**](#_heading=h.ah3ezeb7e2kk)

[1.1 Excluded studies 5](#_heading=h.ku2ixm1aaq5e)

[**Supplement 2. Characteristics of included studies and patients’ baseline features 7**](#_heading=h.aqzxorvuo7br)

[**Supplement 3. Risk of bias assessment of included trials for each outcome 10**](#_heading=h.acmiab7qr6b1)

[3.1 Time In Range (%) 10](#_heading=h.40kar4iv2u7t)

[3.2 Time Below Range (%) 12](#_heading=h.8h9kr3669i9z)

[3.3 Time Below Range <54 mg/dL (%) 14](#_heading=h.r7e3n88g034x)

[3.4 Time Above Range (%) 15](#_heading=h.3qaxexxmarf6)

[3.5 Mean glucose (mg/dL) 16](#_heading=h.hti3lmiueinw)

[3.6 Coefficient of Variation (%) 17](#_heading=h.4g2pblbku350)

[3.7 Severe Hypoglycemia 18](#_heading=h.id8sx7gtzr3t)

[3.8 Diabetic Ketoacidosis (DKA) 19](#_heading=h.nzm8wk1lzw5z)

[**Supplement 4. Funnel plots 20**](#_heading=h.zdo5868jse74)

[4.1 Overall analysis 20](#_heading=h.sx22sy5tfq46)

[4.2 Studies with mean age <18 years 20](#_heading=h.z9oegscqp45i)

[4.2 Studies with mean age ≥18 years 23](#_heading=h.rmmvo3l7ptxs)

[**Supplement 5. Forest plots 27**](#_heading=h.wlkwbjho8ypc)

[5.1 Studies with mean age <18 years 27](#_heading=h.nmvuj2dp6pfu)

[5.2 Studies with mean age ≥18 years 36](#_heading=h.gmnggek16ok7)

[**Supplement 6. Meta-regression-adjusted forest plots for Time In Range 45**](#_heading=h.qnsxegwscvv2)

[6.1 Studies with mean age <18 years 45](#_heading=h.ro0r68vav0i4)

[*6.1.1 TIR adjusted at HbA1c 7.5% 45*](#_heading=h.hpy4o46b3vp9)

[*6.1.2 TIR adjusted at HbA1c 8% 46*](#_heading=h.5ir41xvveabd)

[*6.1.3 TIR adjusted at HbA1c 8.5% 47*](#_heading=h.y9izd9ruvjuk)

[6.2 Studies with mean age ≥18 years 48](#_heading=h.2kxei02ewzrw)

[*6.2.1 TIR adjusted at HbA1c 7.5% 48*](#_heading=h.h7dhwh9s8hfu)

[*6.2.2 TIR adjusted at HbA1c 8% 49*](#_heading=h.yl16fsgig7y7)

[*6.2.3 TIR adjusted at HbA1c 8.5% 50*](#_heading=h.1wxscod0roob)

[**Supplement 7. Sensitivity analysis of Time in Range excluding trials with fewer than 10 participants 51**](#_heading=h.341ek9evlp2x)

[7.1 Funnel plots 51](#_heading=h.mb5s143adi2z)

[*7.1.1 Overall analysis 51*](#_heading=h.fuh6j2ieay6e)

[*7.1.2 Studies with mean age ≥18 years 51*](#_heading=h.8e5v4eex3ml1)

[7.2 Forest plots 52](#_heading=h.9jezzzrcrw8t)

[*7.2.1 TIR stratified by age 52*](#_heading=h.pgnnybgmmqsn)

[*7.2.2 TIR stratified by baseline HbA1c 53*](#_heading=h.9crgg5823sj4)

[*7.2.3 TIR studies with mean age ≥18 years 54*](#_heading=h.w6864wjrjjjw)

[**References 55**](#_heading=h.qjync5ut2u5y)

# Supplement 1. Search strategy

**MEDLINE (via Ovid):**

1. "diabet*".mp.
2. Diabetes Mellitus/
3. exp Diabetes Mellitus Type 1/
4. ((diabetes or "diabetes mellitus" or diabetic*) and ("type 1" or "type I" or "type i" or "insulin dependent" or latent or autoimmune)).mp. [mp=ti, ab, tx, ct, ot, hw, cw, bt, nm, fx, kf, ox, px, rx, ui, sy, ux, mx]
5. "IDDM".mp.
6. "LADA".mp.
7. "Minimed 780g".mp.
8. "780G".mp.
9. "Minimed 670g".mp.
10. "670G".mp.
11. "controlIQ".mp.
12. "control-IQ".mp.
13. "control IQ".mp.
14. "CamAPS Fx".mp.
15. "DBLG1".mp.
16. "Diabeloop".mp.
17. "omnipod 5".mp.
18. "omnipod5".mp.
19. "omnipod".mp.
20. "iLet Bionic".mp.
21. "Bionic pancreas".mp.
22. "automated insulin delivery".mp.
23. "hybrid closed loop".mp.
24. "closed loop".mp.
25. "hybrid closed-loop".mp.
26. "closed-loop".mp.
27. randomized controlled trial.pt.
28. controlled clinical trial.pt.
29. pragmatic clinical trial.pt.
30. randomized.ab.
31. randomised.ab.
32. randomly.ab.
33. 1 or 2 or 3 or 4 or 5 or 6
34. 7 or 8 or 9 or 10 or 11 or 12 or 13 or 14 or 15 or 16 or 17 or 18 or 19 or 20 or 21 or 22 or 23 or 24 or 25 or 26
35. 27 or 28 or 29 or 30 or 31 or 32
36. 33 and 34 and 35
37. remove duplicates from 36

URL to search strategy:

https://ovidsp.ovid.com/ovidweb.cgi?T=JS&NEWS=N&PAGE=main&SHAREDSEARCHID=2JhyuusjLoOSgzumQ4QlbT91S7MzQjRiv7NH02lQhdkKjc4Peg3JRRoN6ei1Db9gj

**CENTRAL:**

(diabet* OR "diabetes mellitus" OR "type 1 diabetes" OR "type 1 diabetes mellitus" OR "type I diabetes" OR "type I diabetes mellitus" OR "insulin dependent diabetes" OR "latent autoimmune diabetes")

AND

("Minimed 780g" OR "780G" OR "Minimed 670g" OR "670G" OR "control IQ" OR "control-IQ" OR "control IQ" OR "CamAPS Fx" OR "DBLG1" OR "Diabeloop" OR "omnipod 5" OR "omnipod5" OR "omnipod" OR "iLet Bionic" OR "bionic pancreas" OR "automated insulin delivery" OR "hybrid closed loop" OR "closed loop" OR "hybrid closed-loop" OR "closed-loop")

AND

(randomized OR randomised OR randomly OR trial OR "controlled trial" OR "clinical trial")

in all text

**Web of Science:**

(diabet* OR "diabetes mellitus" OR "type 1 diabetes" OR "type 1 diabetes mellitus" OR "type I diabetes" OR "type I diabetes mellitus" OR "insulin dependent diabetes" OR "latent autoimmune diabetes")

AND

("Minimed 780g" OR "780G" OR "Minimed 670g" OR "670G" OR "control IQ" OR "control-IQ" OR "control IQ" OR "CamAPS Fx" OR "DBLG1" OR "Diabeloop" OR "omnipod 5" OR "omnipod5" OR "omnipod" OR "iLet Bionic" OR "bionic pancreas" OR "automated insulin delivery" OR "hybrid closed loop" OR "closed loop" OR "hybrid closed-loop" OR "closed-loop")

AND

("randomized controlled trial" OR "randomised controlled trial" OR RCT OR "randomised trial" OR "randomized trial")

**Pubmed:**

(diabet* OR "diabetes mellitus" OR "type 1 diabetes" OR "type 1 diabetes mellitus" OR "type I diabetes" OR "type I diabetes mellitus" OR "insulin dependent diabetes" OR "latent autoimmune diabetes") AND ("Minimed 780g" OR "780G" OR "Minimed 670g" OR "670G" OR "controlIQ" OR "control-IQ" OR "control IQ" OR "CamAPS Fx" OR "DBLG1" OR "Diabeloop" OR "omnipod 5" OR "omnipod5" OR "omnipod" OR "iLet Bionic" OR "Bionic pancreas" OR "automated insulin delivery" OR "hybrid closed loop" OR "closed loop" OR "hybrid closed-loop" OR "closed-loop") AND (randomized controlled trials OR RCT OR randomised controlled trial OR randomised trial OR randomized trial)

## 1.1 Excluded studies

**Not outcome of interest**

| 1 | Tauschmann M, 2017, J Diabetes Sci Technol |
| --- | --- |
| 2 | Weinzimer SA, 2022, Diabetes Technol Ther |
| 3 | Trawley S, 2025, Diabetes Technol Ther |
| 4 | Better postprandial glucose control… doi:10.1002/central/CN-01267773 |
| 5 | Evaluation of overnight insulinemia… doi:10.1002/central/CN-01471893 |
| 6 | Ly TT, 2014, Diabetes Care |
| 7 | Validation of a closed loop system… doi:10.1002/central/CN-01078544 |

**Not eligible intervention**

| 1 | Garcia-Tirado J, 2021, Diabetes Care |
| --- | --- |
| 2 | Dovc K, 2023, Diabetes Technol Ther |
| 3 | Ly TT, 2016, Diabetes Technol Ther |
| 4 | Nwokolo M, 2023, Diabetes Technol Ther |
| 5 | Biester T, 2019, Diabetes Obes Metab |
| 6 | Tsoukas MA, 2021, Lancet Digit Health |
| 7 | Nabhan ZM, 2009, Pediatr Diabetes |
| 8 | RCT CSII vs MDI… doi:10.1002/central/CN-01669241 |
| 9 | Burnside M, 2020, J Diabetes Metab Disord |
| 10 | EUCTR2019-001631-31-DK, doi:10.1002/central/CN-02068677 |
| 11 | Dauber A, 2013, Diabetes Care |
| 12 | Tubiana-Rufi N, 2019, Pediatr Diabetes (abstract) |
| 13 | Kim JY, 2024, Diabetologia |
| 14 | Leelarathna L, 2014, Diabetes Care |
| 15 | Day-and-night CL adolescents… doi:10.1002/central/CN-01020200 |
| 16 | Boughton CK, 2023, Diabetologia (EASD abstract) |
| 17 | Ware J, 2023, Diabetes Technol Ther |
| 18 | Michaels VR, 2025, Diabet Med |
| 19 | Nimri R, 2014, Diabetes Care |
| 20 | Nimri R, 2017, Diabetes Obes Metab |
| 21 | Schmidt S, 2013, J Diabetes Sci Technol |
| 22 | Nimri R, 2014, Pediatr Diabetes |
| 23 | Bionic pancreas preadolescents… doi:10.1002/central/CN-01135454 |
| 24 | Predictive hyper/hypoglycemia minimization… doi:10.1002/central/CN-01739813 |
| 25 | Schoelwer MJ, 2020, Diabetes Technol Ther |
| 26 | Elleri D, 2014, Diabetes Obes Metab |
| 27 | Nimri R, 2014, Diabetes Technol Ther |
| 28 | Paldus B, 2019, Diabetes Technol Ther |

**Not population of interest**

| 1 | Davis GM, 2023, Diabetes Technol Ther |
| --- | --- |
| 2 | Factors affecting closed-loop inpatient… doi:10.1002/central/CN-01709031 |
| 3 | Elbarbary NS, 2022, Diabetes Res Clin Pract |
| 4 | Ramadan trial… doi:10.1002/central/CN-02809716 |
| 5 | Castellanos LE, 2023, Diabetes Care |

**Not study design of interest**

| 1 | Zone-MPC fault detection… doi:10.1002/central/CN-01669238 |
| --- | --- |
| 2 | NCT04510506, doi:10.1002/central/CN-02137998 |
| 3 | Artificial pancreas multi-night study… doi:10.1002/central/CN-01103843 |
| 4 | At-home randomized crossover… doi:10.1002/central/CN-02203852 |
| 5 | Ly TT, 2017, Pediatr Diabetes |
| 6 | De Meulemeester J, 2025, Diabetologia |
| 7 | Amole M, 2021, Fed Pract |
| 8 | Kubilay E, 2024, Diabet Med |

**Not publication type of interest**

| 1 | Kong YW, 2023, Pilot Feasibility Stud |
| --- | --- |
| 2 | Cohen O, 2023, Diabetes (abstract) |
| 3 | NCT04025762, doi:10.1002/central/CN-01965615 |
| 4 | NL-OMON trial, doi:10.1002/central/CN-02718017 |
| 5 | van den Heuvel T, 2024, J Diabetes Sci Technol |
| 6 | Kudva YC, 2025, Diabetes Technol Ther |
| 7 | Ware J, 2024, Diabetes Care |
| 8 | Wheeler BJ, 2022, Acta Diabetol |
| 9 | Hood KK, 2021, Diabetes Technol Ther |
| 10 | Hood KK, 2022, Diabetes Obes Metab |
| 11 | Ekhlaspour L, 2023, J Diabetes Sci Technol |
| 12 | Schoelwer MJ, 2021, Diabetes Technol Ther |
| 13 | Omnipod recently diagnosed… doi:10.1002/central/CN-02497642 |
| 14 | Halliday JA, 2024, BMJ Open Diabetes Res Care |
| 15 | Thabit H, 2015, Lancet (supplement) |
| 16 | Breton MD, 2020, N Engl J Med (Reply) |

# Supplement 2. Characteristics of included studies and patients’ baseline features

| **Author** | **Year** | **Type of study** | **No. of patients** | **Gender**  **(Female, %)** | **Study duration**  **(weeks)** | **Age**  **(years)** | **HbA1c (%)** | **Duration of diabetes** | **HCL system** | **Comparator** |
| --- | --- | --- | --- | --- | --- | --- | --- | --- | --- | --- |
| Tauschmann M[^1^](https://paperpile.com/c/4MUUKd/IbVu) | 2018 | RCT | 86 | 51.2 | 12 | 23.2±18.4 | 7.9±0·6 | 12.65±9.6 | CamAPS Fx | SAP |
| Benhamou PY[^2^](https://paperpile.com/c/4MUUKd/1701) | 2019 | RCT † | 63 | 62 | 12 | 48.2±13.4 | 7.6±0.9 | 28.0±13.6 | DBLG1 | SAP |
| Brown SA[^3^](https://paperpile.com/c/4MUUKd/nHol) | 2019 | RCT | 168 | 50 | 26 | 33±17 | 7.4±0.9 | 16.4±13.6 | Control IQ | SAP |
| Breton MD[^4^](https://paperpile.com/c/4MUUKd/KCYJ) | 2020 | RCT | 101 | 49.5 | 16 | 11.1±2.2 | 7.8±1.0 | 5.5±2.8 | Control IQ | SAP/PLGS |
| Brown SA[^5^](https://paperpile.com/c/4MUUKd/01Bv) | 2020 | RCT | 109 | 48.6 | 13 | 33±15.5 | 7.1±0.8 | 18.2±17.2 | Control IQ | PLGS |
| McAuley SA[^6^](https://paperpile.com/c/4MUUKd/H50G) | 2020 | RCT | 120 | 53 | 26 | 44.2±11.8 | 7.5±0.9 | 24.1±12.3 | MiniMed 670G | MDI/CSII |
| Abraham MB[^7^](https://paperpile.com/c/4MUUKd/WJtC) | 2021 | RCT | 135 | 56.3 | 26 | 15.3±3.1 | 8.0±1.0 | 7.7±4.3 | MiniMed 670G | MDI/CSII±CGM |
| Bergenstal RM[^8^](https://paperpile.com/c/4MUUKd/16UO) | 2021 | RCT † | 113 | 62 | 12 | 19±4 | 7.9±0.7 | 11.3±6 | MiniMed 670G | Medtronic AHCL |
| Burckhardt MA[^9^](https://paperpile.com/c/4MUUKd/HaBa) | 2021 | RCT † | 16 | 71 | 8 | 35.8±11.2 | 7.8±1.2 | 24.2±11.3 | MiniMed 670G | CSII |
| Collyns OJ[^10^](https://paperpile.com/c/4MUUKd/9oEX) | 2021 | RCT † | 60 | 59.3 | 4 | 23.5 (7.0-65) | 7.6±0.9 | 13.2±10.2 | MiniMed 780G | PLGS |
| Boughton CK[^11^](https://paperpile.com/c/4MUUKd/6rAA) | 2022 | RCT † | 37 | 43 | 16 | 67±5.4 | 7·4±0·9 | 39±11.6 | CamAPS Fx | SAP |
| Boughton CK[^12^](https://paperpile.com/c/4MUUKd/esoy) | 2022 | RCT | 97 | 44 | 104 | 12±2 | 10.6±1.7 | 9.5±6.2 | CamAPS Fx | MDI/CSII±CGM |
| Choudhary P[^13^](https://paperpile.com/c/4MUUKd/ItQZ) | 2022 | RCT | 82 | 46.3 | 26 | 40.6±13.12 | 9.1±0.8 | 18.5±10.7 | MiniMed 780G | MDI+isCGM |
| Garcia-Tirado J[^14^](https://paperpile.com/c/4MUUKd/HTIo) | 2022 | RCT † | 35 | 64.7 | 8 | 41±14 | 6.8±0.9 | 21±13 | Control IQ | PLGS |
| Kariyawasam D[^15^](https://paperpile.com/c/4MUUKd/0Sz4) | 2022 | RCT † | 21 | 52.4 | 6 | 8.1±2.6 | 7.2±0.6 | 5.7±3.0 | DBLG1 | SAP |
| Kruger D[^16^](https://paperpile.com/c/4MUUKd/ti4U) | 2022 | RCT | 161 | 48.4 | 13 | 44±15 | 7.6±1.2 | 27±14 | iLet Bionic Pancreas | HCL/MDI/CSI/  + CGM |
| Matejko B[^17^](https://paperpile.com/c/4MUUKd/Vyli) | 2022 | RCT | 37 | 43.2 | 13 | 40.4±8.1 | 7.2 ±1.0 | 17.4±12.2 | MiniMed 780G | MDI |
| McAuley SA[^18^](https://paperpile.com/c/4MUUKd/O3w5) | 2022 | RCT † | 30 | 63.3 | 17.3 | 67±5 | 7.5±0.6 | 35±21 | MiniMed 670G | SAP±LGS |
| Messer LH[^19^](https://paperpile.com/c/4MUUKd/alSQ) | 2022 | RCT | 165 | 42 | 13 | 12±3 | 8±1.2 | 6.32±4 | iLet Bionic Pancreas | HCL/MDI/CSII+ CGM/LGS |
| Reiss AL[^20^](https://paperpile.com/c/4MUUKd/LVFB) | 2022 | RCT | 42 | 54.8 | 26 | 14 to 17 | 9.00 | since before  age 8 years | MiniMed 670G | MDI/CSII±CGM |
| von dem Berge T[^21^](https://paperpile.com/c/4MUUKd/RSc6) | 2022 | RCT † | 38 | 55.3 | 8 | 8.7±3.5 | 7.4±0.9 | 4.3±2.8 | MiniMed 670G | PLGS |
| Ware J[^22^](https://paperpile.com/c/4MUUKd/tDbn) | 2022 | RCT † | 74 | 42 | 16 | 5.6±1.6 | 7.3±0.7 | 2.6±1.8 | CamAPS Fx | SAP |
| Ware J[^23^](https://paperpile.com/c/4MUUKd/XpwF) | 2022 | RCT | 46 | 57.1 | 26 | 12.6 ± 2.6 | 8.0±0.8 | 6.5±2.6 | CamAPS Fx | CSII |
| Franc S[^24^](https://paperpile.com/c/4MUUKd/iodz) | 2023 | RCT | 84 | N.A. | 13 | 42.1±12.4 | 7.8±1.0 | 24.8±11.1 | DBLG1 | SAP |
| Garg SK[^25^](https://paperpile.com/c/4MUUKd/CdWg) | 2023 | RCT | 302 | 54.3 | 26 | 37.9±18.8 | 8.2±0.8 | 20.6±13.0 | MiniMed 670G | CSII |
| Renard E[^26^](https://paperpile.com/c/4MUUKd/pPvl) | 2023 | RCT | 72 | 62 | 12 | 47.2±12.7 | 7.0±0.6 | 27.9±12.8 | Control IQ | CSII + CGM |
| Rossi A[^27,28^](https://paperpile.com/c/4MUUKd/KRR6+RBNr) | 2023 | RCT | 48 | 26 | 26 | 41.5±3 | 7.4±2.3 | 22.5±2 | MiniMed 780G | PLGS |
| van den Heuvel T[^29^](https://paperpile.com/c/4MUUKd/I8nv) | 2023 | RCT | 13 | 46.2 | 26 | 40.8±14.6 | 9.2±0.6 | 15.4±9.5 | MiniMed 780G | MDI+RT-CGM |
| Wadwa RP[^30^](https://paperpile.com/c/4MUUKd/vIUa) | 2023 | RCT | 102 | 51 | 13 | 4.0±1.2 | 7.6±1.1 | 6 months  to 5 years | Control IQ | MDI/CSII+CGM |
| Battelino T[^31,32^](https://paperpile.com/c/4MUUKd/5kmu+mYBK) | 2024 | RCT † | 98 | 49 | 26 | 4.72±1.16 | 7.5±1 | N.A. | MiniMed 780G | PLGS |
| Boucsein A[^33^](https://paperpile.com/c/4MUUKd/b3xy) | 2024 | RCT | 80 | 57.5 | 13 | 16.8±14.1 | 10.5±1.7 | 6.9 (4.1; 9.6)^*^ | MiniMed 780G | MDI/CSII±CGM |
| Christensen MB[^34^](https://paperpile.com/c/4MUUKd/RipL) | 2024 | RCT | 40 | N.A. | 14 | 52±11 | 8.3±2.8 | 29±13 | MiniMed 780G | CSII+CGM  /isCGM |
| Kudva YC[^35^](https://paperpile.com/c/4MUUKd/saQx) | 2024 | RCT † | 82 | 45 | 12 | 71±4 | 7.2±0.9 | 42±17 | Control IQ | PLGS/SAP |
| Lee MH[^36^](https://paperpile.com/c/4MUUKd/6T8G) | 2024 | RCT | 9 | 54 | 26 | 47 (41; 59)^*^ | 7.7 (7.4;8.1)^*^ | 27.7  (24.1; 32.1)^*^ | MiniMed 670G | MDI/CSII |
| Renard E[^37^](https://paperpile.com/c/4MUUKd/JQd1) | 2024 | RCT | 194 | 59.8 | 13 | 36±14 | 8.5±0.8 | 19.5±11.1 | Omnipod 5 | CSII+CGM |
| Abraham MB[^38^](https://paperpile.com/c/4MUUKd/OTg0) | 2025 | RCT | 42 | 57,5 | 24 | 16.2±2.5 | 9.8±1.1 | 9.7±4.2 | MiniMed 780G | CSII+CGM |
| Wilmot EG[^39^](https://paperpile.com/c/4MUUKd/C0FB) | 2025 | RCT | 188 | 45.7 | 13 | 24 (6; 66.7)^*^ | 8.1 (7.6; 8.5)^*^ | 11 | Omnipod 5 | MDI+CGM |

HCL, hybrid closed-loop; AHCL, advanced hybrid closed-loop; MDI, multiple daily injections; CSII, continuous subcutaneous insulin infusion; SAP, sensor augmented pump; LGS, low glucose suspend; PLGS, predictive low glucose suspend; CGM, continuous glucose monitoring; RT-CGM, real time CGM; isCGM, intermittently scanned CGM; N.A., not available; RCT, randomized controlled trial. †crossover design; ^*^median (interquartile range)

# Supplement 3. Risk of bias assessment of included trials for each outcome

## 3.1 Time In Range (%)

| **Study design: parallel arms** | | | | | | | |
| --- | --- | --- | --- | --- | --- | --- | --- |
| **Study ID** | **Randomization process** | **Deviations from intended intervention** | **Missing outcome data** | **Measurement of outcome** | **Selection of reported result** | **Overall** | |
| Abraham MB 2021 | Low risk | Low risk | Low risk | Low risk | Low risk | Low risk | |
| Abraham MB 2025 | Low risk | Low risk | Low risk | Low risk | Low risk | Low risk | |
| Boucsein A 2024 | Low risk | Some concerns | Low risk | Low risk | Low risk | Low risk | |
| Boughton CK 2022 | Low risk | Low risk | Low risk | Low risk | Low risk | Low risk | |
| Breton MD 2020 | Low risk | Low risk | Low risk | Low risk | Low risk | Low risk | |
| Brown SA 2019 | Low risk | Low risk | Some concerns | Low risk | Low risk | Some concerns | |
| Brown SA 2020 | Low risk | Low risk | Low risk | Low risk | Low risk | Low risk | |
| Choudhary P 2022 | Low risk | Low risk | Low risk | Low risk | Low risk | Low risk | |
| Christensen MB 2024 | Low risk | Low risk | Low risk | Low risk | Low risk | Low risk | |
| Franc S 2023 | Some concerns | Some concerns | Some concerns | Low risk | Low risk | Some concerns | |
| Garg SK 2023 | Low risk | Low risk | Some concerns | Low risk | Low risk | Some concerns | |
| Kruger D 2022 | Low risk | Low risk | Low risk | Low risk | Low risk | Low risk | |
| Lee MH 2024 | Low risk | Low risk | Low risk | Low risk | Low risk | Low risk | |
| Matejko B 2022 | Low risk | Low risk | Low risk | Low risk | Low risk | Low risk | |
| McAuley SA 2020 | Low risk | Low risk | Low risk | Low risk | Low risk | Low risk | |
| Messer LH 2022 | Low risk | Low risk | Low risk | Low risk | Low risk | Low risk | |
| Reiss AL 2022 | Low risk | Low risk | Low risk | Low risk | Some concerns | Some concerns | |
| Renard E 2024 | Low risk | Low risk | Low risk | Low risk | Low risk | Low risk | |
| Rossi A 2023 | Low risk | Low risk | Low risk | Low risk | Low risk | Low risk | |
| Tauschmann M 2018 | Low risk | Low risk | Low risk | Low risk | Low risk | Low risk | |
| van den Heuvel T 2023 | Low risk | Low risk | Low risk | Low risk | Low risk | Low risk | |
| Wadwa RP 2023 | Low risk | Low risk | Low risk | Low risk | Low risk | Low risk | |
| Ware J 2022 | Low risk | Low risk | Low risk | Low risk | Low risk | Low risk | |
| Wilmot EG 2025 | Some concerns | Low risk | Some concerns | Low risk | Low risk | Some concerns | |
| **Study design: cross-over** | | | | | | | |
| **Study ID** | **Randomization process** | **Period and carryover effects** | **Deviations from intended intervention** | **Missing outcome data** | **Measurement of outcome** | **Selection of the reported result** | **Overall** |
| Battelino T 2024 | Low risk | Low risk | Low risk | Low risk | Low risk | Low risk | Low risk |
| Benhamou PY 2019 | Low risk | Low risk | Low risk | Low risk | Low risk | Low risk | Low risk |
| Bergenstal RM 2021 | Low risk | Low risk | Low risk | Low risk | Low risk | Low risk | Low risk |
| Boughton CK 2022 | Low risk | Low risk | Low risk | Low risk | Low risk | Low risk | Low risk |
| Burckhardt MA 2021 | Low risk | Low risk | Low risk | Low risk | Low risk | Low risk | Low risk |
| Collyns OJ 2021 | Some concerns | Some concerns | Low risk | Low risk | Low risk | Low risk | Some concerns |
| Garcia-Tirado J 2022 | Low risk | Some concerns | Low risk | Low risk | Low risk | Some concerns | High risk |
| Kariyawasam D 2022 | Low risk | Low risk | Low risk | Low risk | Low risk | Low risk | Low risk |
| Kudva YC et al, 2024 | Low risk | Low risk | Low risk | Low risk | Low risk | Low risk | Low risk |
| McAuley SA 2022 | Low risk | Low risk | Low risk | Low risk | Low risk | Low risk | Low risk |
| von dem Berge T 2022 | Some concerns | Some concerns | Low risk | Some concerns | Low risk | Low risk | Some concerns |
| Ware J 2022 | Low risk | Low risk | Low risk | Low risk | Low risk | Low risk | Low risk |

##

## 3.2 Time Below Range (%)

| **Study design: parallel arms** | | | | | | | |
| --- | --- | --- | --- | --- | --- | --- | --- |
| **Study ID** | **Randomization process** | **Deviations from intended intervention** | **Missing outcome data** | **Measurement of outcome** | **Selection of reported result** | **Overall** | |
| Abraham MB 2021 | Low risk | Low risk | Low risk | Low risk | Low risk | Low risk | |
| Abraham MB 2025 | Low risk | Low risk | Low risk | Low risk | Low risk | Low risk | |
| Boucsein A 2024 | Low risk | Some concerns | Low risk | Low risk | Low risk | Low risk | |
| Boughton CK 2022 | Low risk | Low risk | Low risk | Low risk | Low risk | Low risk | |
| Breton MD 2020 | Low risk | Low risk | Low risk | Low risk | Low risk | Low risk | |
| Brown SA 2019 | Low risk | Low risk | Some concerns | Low risk | Low risk | Some concerns | |
| Brown SA 2020 | Low risk | Low risk | Low risk | Low risk | Low risk | Low risk | |
| Choudhary P 2022 | Low risk | Low risk | Low risk | Low risk | Low risk | Low risk | |
| Christensen MB 2024 | Low risk | Low risk | Low risk | Low risk | Low risk | Low risk | |
| Franc S 2023 | Some concerns | Some concerns | Some concerns | Low risk | Low risk | Some concerns | |
| Garg SK 2023 | Low risk | Low risk | Some concerns | Low risk | Low risk | Some concerns | |
| Kruger D 2022 | Low risk | Low risk | Low risk | Low risk | Low risk | Low risk | |
| Lee MH 2024 | Low risk | Low risk | Low risk | Low risk | Low risk | Low risk | |
| Matejko B 2022 | Low risk | Low risk | Low risk | Low risk | Low risk | Low risk | |
| McAuley SA 2020 | Low risk | Low risk | Low risk | Low risk | Low risk | Low risk | |
| Messer LH 2022 | Low risk | Low risk | Low risk | Low risk | Low risk | Low risk | |
| Reiss AL 2022 | Low risk | Low risk | Low risk | Low risk | Some concerns | Some concerns | |
| Renard E 2024 | Low risk | Low risk | Low risk | Low risk | Low risk | Low risk | |
| Rossi A 2023 | Low risk | Low risk | Low risk | Low risk | Low risk | Low risk | |
| Tauschmann M 2018 | Low risk | Low risk | Low risk | Low risk | Low risk | Low risk | |
| van den Heuvel T 2023 | Low risk | Low risk | Low risk | Low risk | Low risk | Low risk | |
| Wadwa RP 2023 | Low risk | Low risk | Low risk | Low risk | Low risk | Low risk | |
| Ware J 2022 | Low risk | Low risk | Low risk | Low risk | Low risk | Low risk | |
| Wilmot EG 2025 | Some concerns | Low risk | Some concerns | Low risk | Low risk | Some concerns | |
| **Study design: cross-over** | | | | | | | |
| **Study ID** | **Randomization process** | **Period and carryover effects** | **Deviations from intended intervention** | **Missing outcome data** | **Measurement of outcome** | **Selection of the reported result** | **Overall** |
| Battelino T 2024 | Low risk | Low risk | Low risk | Low risk | Low risk | Low risk | Low risk |
| Benhamou PY 2019 | Low risk | Low risk | Low risk | Low risk | Low risk | Low risk | Low risk |
| Bergenstal RM 2021 | Low risk | Low risk | Low risk | Low risk | Low risk | Low risk | Low risk |
| Burckhardt MA 2021 | Low risk | Low risk | Low risk | Low risk | Low risk | Low risk | Low risk |
| Collyns OJ 2021 | Some concerns | Some concerns | Low risk | Low risk | Low risk | Low risk | Some concerns |
| Garcia-Tirado J 2022 | Low risk | Some concerns | Low risk | Low risk | Low risk | Some concerns | High risk |
| Kudva YC et al, 2024 | Low risk | Low risk | Low risk | Low risk | Low risk | Low risk | Low risk |
| McAuley SA 2022 | Low risk | Low risk | Low risk | Low risk | Low risk | Low risk | Low risk |
| von dem Berge T al. 2022 | Some concerns | Some concerns | Low risk | Some concerns | Low risk | Low risk | Some concerns |
| Ware J 2022 | Low risk | Low risk | Low risk | Low risk | Low risk | Low risk | Low risk |

##

## 3.3 Time Below Range <54 mg/dL (%)

| **Study design: parallel arms** | | | | | | | |
| --- | --- | --- | --- | --- | --- | --- | --- |
| **Study ID** | **Randomization process** | **Deviations from intended intervention** | **Missing outcome data** | **Measurement of outcome** | **Selection of reported result** | **Overall** | |
| Abraham MB 2021 | Low risk | Low risk | Low risk | Low risk | Low risk | Low risk | |
| Abraham MB 2025 | Low risk | Low risk | Low risk | Low risk | Low risk | Low risk | |
| Boucsein A 2024 | Low risk | Some concerns | Low risk | Low risk | Low risk | Low risk | |
| Boughton CK 2022 | Low risk | Low risk | Low risk | Low risk | Some concerns | Some concerns | |
| Breton MD 2020 | Low risk | Low risk | Low risk | Low risk | Low risk | Low risk | |
| Messer LH 2022 | Low risk | Low risk | Low risk | Low risk | Low risk | Low risk | |
| Reiss AL 2022 | Low risk | Low risk | Low risk | Low risk | Some concerns | Some concerns | |
| Wadwa RP 2023 | Low risk | Low risk | Low risk | Low risk | Low risk | Low risk | |
| Ware J 2022 | Low risk | Low risk | Low risk | Low risk | Low risk | Low risk | |
| **Study design: cross-over** | | | | | | | |
| **Study ID** | **Randomization process** | **Period and carryover effects** | **Deviations from intended intervention** | **Missing outcome data** | **Measurement of outcome** | **Selection of the reported result** | **Overall** |
| Battelino T 2024 | Low risk | Low risk | Low risk | Low risk | Low risk | Low risk | Low risk |
| Kariyawasam 2022 | Low risk | Low risk | Low risk | Low risk | Low risk | Low risk | Low risk |
| von dem Berge 2022 | Some concerns | Some concerns | Low risk | Some concerns | Low risk | Low risk | Some concerns |
| Ware 2022 | Low risk | Low risk | Low risk | Low risk | Low risk | Low risk | Low risk |

## 3.4 Time Above Range (%)

| **Study design: parallel arms** | | | | | | | |
| --- | --- | --- | --- | --- | --- | --- | --- |
| **Study ID** | **Randomization process** | **Deviations from intended intervention** | **Missing outcome data** | **Measurement of outcome** | **Selection of reported result** | **Overall** | |
| Abraham 2021 | Low risk | Low risk | Low risk | Low risk | Low risk | Low risk | |
| Abraham 2025 | Low risk | Low risk | Low risk | Low risk | Low risk | Low risk | |
| Boucsein 2024 | Low risk | Some concerns | Low risk | Low risk | Low risk | Low risk | |
| Boughton 2022 | Low risk | Low risk | Low risk | Low risk | Low risk | Low risk | |
| Breton 2020 | Low risk | Low risk | Low risk | Low risk | Low risk | Low risk | |
| Messer 2022 | Low risk | Low risk | Low risk | Low risk | Low risk | Low risk | |
| Reiss 2022 | Low risk | Low risk | Low risk | Low risk | Some concerns | Some concerns | |
| Ware 2022 | Low risk | Low risk | Low risk | Low risk | Low risk | Low risk | |
| **Study design: cross-over** | | | | | | | |
| **Study ID** | **Randomization process** | **Period and carryover effects** | **Deviations from intended intervention** | **Missing outcome data** | **Measurement of outcome** | **Selection of the reported result** | **Overall** |
| Battelino 2024 | Low risk | Low risk | Low risk | Low risk | Low risk | Low risk | Low risk |
| Kariyawasam 2022 | Low risk | Low risk | Low risk | Low risk | Low risk | Low risk | Low risk |
| von dem Berge 2022 | Some concerns | Some concerns | Low risk | Some concerns | Low risk | Low risk | Some concerns |
| Ware 2022 | Low risk | Low risk | Low risk | Low risk | Low risk | Low risk | Low risk |

## 3.5 Mean glucose (mg/dL)

| **Study design: parallel arms** | | | | | | | |
| --- | --- | --- | --- | --- | --- | --- | --- |
| **Study ID** | **Randomization process** | **Deviations from intended intervention** | **Missing outcome data** | **Measurement of outcome** | **Selection of reported result** | **Overall** | |
| Abraham 2021 | Low risk | Low risk | Low risk | Low risk | Low risk | Low risk | |
| Abraham 2025 | Low risk | Low risk | Low risk | Low risk | Low risk | Low risk | |
| Boucsein 2024 | Low risk | Some concerns | Low risk | Low risk | Low risk | Low risk | |
| Boughton 2022 | Low risk | Low risk | Low risk | Low risk | Low risk | Low risk | |
| Breton 2020 | Low risk | Low risk | Low risk | Low risk | Low risk | Low risk | |
| Messer 2022 | Low risk | Low risk | Low risk | Low risk | Low risk | Low risk | |
| Reiss 2022 | Low risk | Low risk | Low risk | Low risk | Some concerns | Some concerns | |
| Wadwa 2023 | Low risk | Low risk | Low risk | Low risk | Low risk | Low risk | |
| Ware 2022 | Low risk | Low risk | Low risk | Low risk | Low risk | Low risk | |
| **Study design: cross-over** | | | | | | | |
| **Study ID** | **Randomization process** | **Period and carryover effects** | **Deviations from intended intervention** | **Missing outcome data** | **Measurement of outcome** | **Selection of the reported result** | **Overall** |
| Battelino 2024 | Low risk | Low risk | Low risk | Low risk | Low risk | Low risk | Low risk |
| Kariyawasam 2022 | Low risk | Low risk | Low risk | Low risk | Low risk | Low risk | Low risk |
| von dem Berge 2022 | Some concerns | Some concerns | Low risk | Some concerns | Low risk | Low risk | Some concerns |
| Ware 2022 | Low risk | Low risk | Low risk | Low risk | Low risk | Low risk | Low risk |

## 3.6 Coefficient of Variation (%)

| **Study design: parallel arms** | | | | | | | |
| --- | --- | --- | --- | --- | --- | --- | --- |
| **Study ID** | **Randomization process** | **Deviations from intended intervention** | **Missing outcome data** | **Measurement of outcome** | **Selection of reported result** | **Overall** | |
| Abraham 2021 | Low risk | Low risk | Low risk | Low risk | Low risk | Low risk | |
| Abraham 2025 | Low risk | Low risk | Low risk | Low risk | Low risk | Low risk | |
| Boucsein 2024 | Low risk | Some concerns | Low risk | Low risk | Low risk | Low risk | |
| Boughton 2022 | Low risk | Low risk | Low risk | Low risk | Low risk | Low risk | |
| Breton 2020 | Low risk | Low risk | Low risk | Low risk | Low risk | Low risk | |
| Messer 2022 | Low risk | Low risk | Low risk | Low risk | Low risk | Low risk | |
| Reiss 2022 | Low risk | Low risk | Low risk | Low risk | Some concerns | Some concerns | |
| Wadwa 2023 | Low risk | Low risk | Low risk | Low risk | Low risk | Low risk | |
| Ware 2022 | Low risk | Low risk | Low risk | Low risk | Low risk | Low risk | |
| **Study design: cross-over** | | | | | | | |
| **Study ID** | **Randomization process** | **Period and carryover effects** | **Deviations from intended intervention** | **Missing outcome data** | **Measurement of outcome** | **Selection of the reported result** | **Overall** |
| Battelino 2024 | Low risk | Low risk | Low risk | Low risk | Low risk | Low risk | Low risk |
| Kariyawasam 2022 | Low risk | Low risk | Low risk | Low risk | Low risk | Low risk | Low risk |
| von dem Berge 2022 | Some concerns | Some concerns | Low risk | Some concerns | Low risk | Some concerns | High risk |
| Ware 2022 | Low risk | Low risk | Low risk | Low risk | Low risk | Low risk | Low risk |

## 3.7 Severe Hypoglycemia

| **Study design: parallel arms** | | | | | | | |
| --- | --- | --- | --- | --- | --- | --- | --- |
| **Study ID** | **Randomization process** | **Deviations from intended intervention** | **Missing outcome data** | **Measurement of outcome** | **Selection of reported result** | **Overall** | |
| Abraham 2021 | Low risk | Low risk | Low risk | Low risk | Low risk | Low risk | |
| Abraham 2025 | Low risk | Low risk | Low risk | Low risk | Low risk | Low risk | |
| Boucsein 2024 | Low risk | Some concerns | Low risk | Low risk | Low risk | Low risk | |
| Boughton 2022 | Low risk | Low risk | Low risk | Low risk | Low risk | Low risk | |
| Breton 2020 | Low risk | Low risk | Low risk | Low risk | Low risk | Low risk | |
| Messer 2022 | Low risk | Low risk | Low risk | Low risk | Low risk | Low risk | |
| Reiss 2022 | Low risk | Low risk | Low risk | Low risk | Some concerns | Some concerns | |
| Wadwa 2023 | Low risk | Low risk | Low risk | Low risk | Low risk | Low risk | |
| Ware 2022 | Low risk | Low risk | Low risk | Low risk | Low risk | Low risk | |
| **Study design: cross-over** | | | | | | | |
| **Study ID** | **Randomization process** | **Period and carryover effects** | **Deviations from intended intervention** | **Missing outcome data** | **Measurement of outcome** | **Selection of the reported result** | **Overall** |
| Battelino 2024 | Low risk | Low risk | Low risk | Low risk | Low risk | Low risk | Low risk |
| Kariyawasam 2022 | Low risk | Low risk | Low risk | Low risk | Low risk | Low risk | Low risk |
| von dem Berge 2022 | Some concerns | Some concerns | Low risk | Some concerns | Low risk | Some concerns | High risk |
| Ware 2022 | Low risk | Low risk | Low risk | Low risk | Low risk | Low risk | Low risk |

##

## 3.8 Diabetic Ketoacidosis (DKA)

| **Study design: parallel arms** | | | | | | | |
| --- | --- | --- | --- | --- | --- | --- | --- |
| **Study ID** | **Randomization process** | **Deviations from intended intervention** | **Missing outcome data** | **Measurement of outcome** | **Selection of reported result** | **Overall** | |
| Abraham 2021 | Low risk | Low risk | Low risk | Low risk | Low risk | Low risk | |
| Abraham 2025 | Low risk | Low risk | Low risk | Low risk | Low risk | Low risk | |
| Boucsein 2024 | Low risk | Some concerns | Low risk | Low risk | Low risk | Low risk | |
| Boughton 2022 | Low risk | Low risk | Low risk | Low risk | Low risk | Low risk | |
| Breton 2020 | Low risk | Low risk | Low risk | Low risk | Low risk | Low risk | |
| Messer 2022 | Low risk | Low risk | Low risk | Low risk | Low risk | Low risk | |
| Reiss 2022 | Low risk | Low risk | Low risk | Low risk | Some concerns | Some concerns | |
| Wadwa 2023 | Low risk | Low risk | Low risk | Low risk | Low risk | Low risk | |
| Ware 2022 | Low risk | Low risk | Low risk | Low risk | Low risk | Low risk | |
| **Study design: cross-over** | | | | | | | |
| **Study ID** | **Randomization process** | **Period and carryover effects** | **Deviations from intended intervention** | **Missing outcome data** | **Measurement of outcome** | **Selection of the reported result** | **Overall** |
| Battelino 2024 | Low risk | Low risk | Low risk | Low risk | Low risk | Low risk | Low risk |
| Kariyawasam 2022 | Low risk | Low risk | Low risk | Low risk | Low risk | Low risk | Low risk |
| von dem Berge 2022 | Some concerns | Some concerns | Low risk | Some concerns | Low risk | Some concerns | High risk |
| Ware 2022 | Low risk | Low risk | Low risk | Low risk | Low risk | Low risk | Low risk |

# Supplement 4. Funnel plots

## 4.1 Overall analysis

##
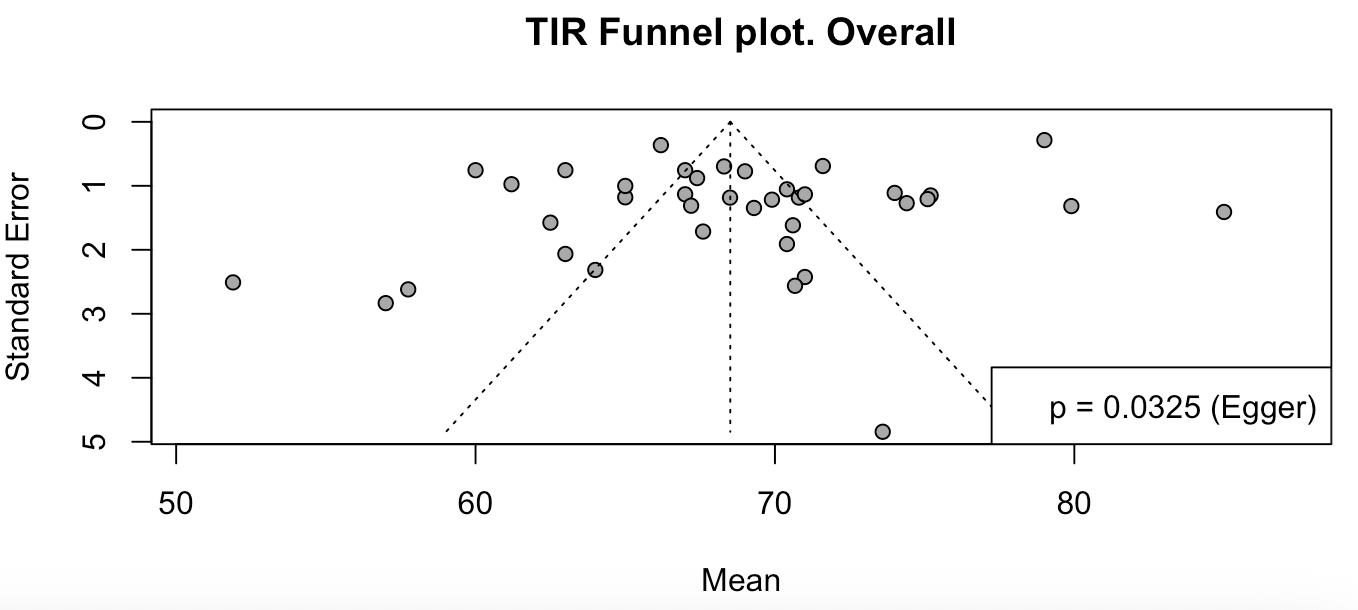
4.2 Studies with mean age <18 years

**
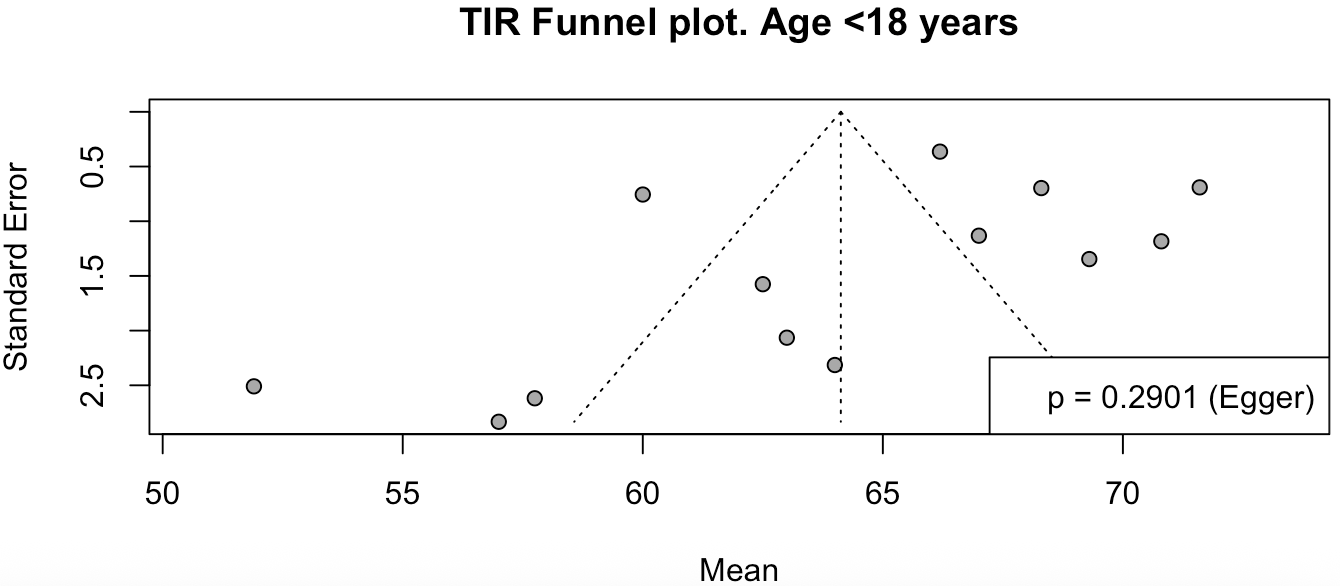

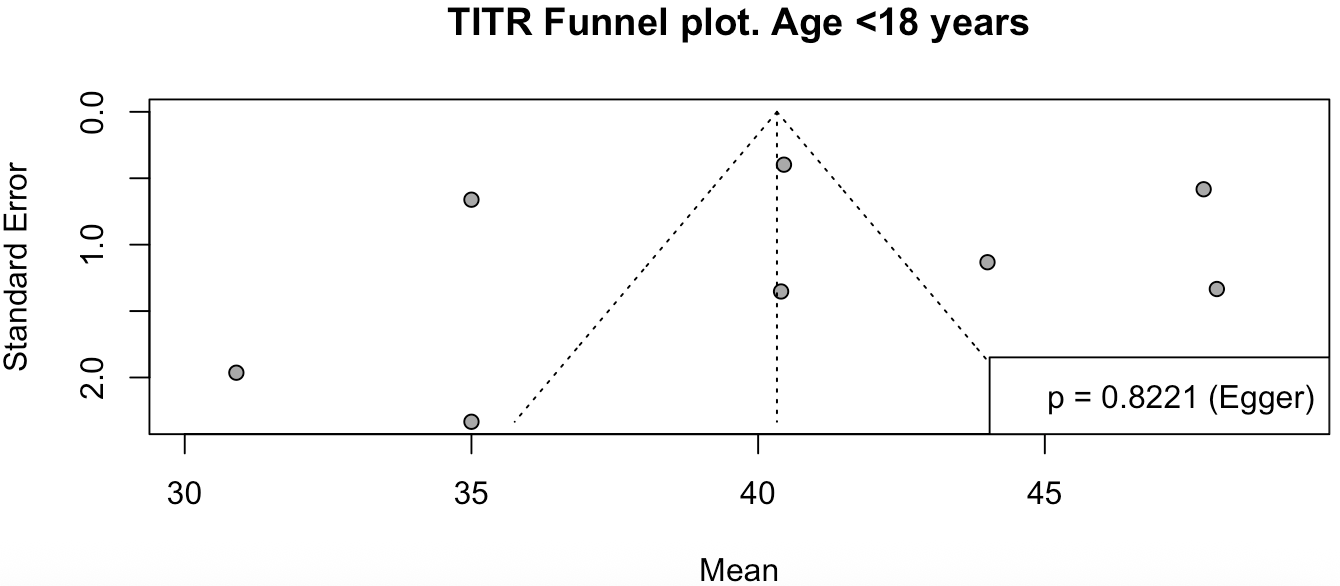

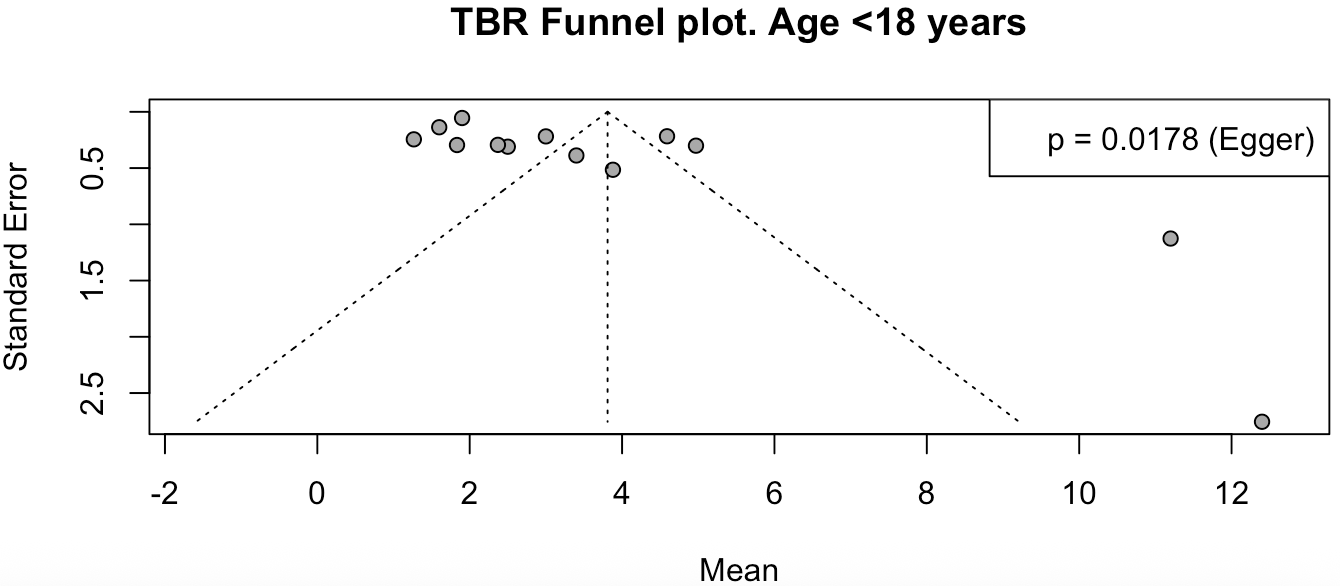

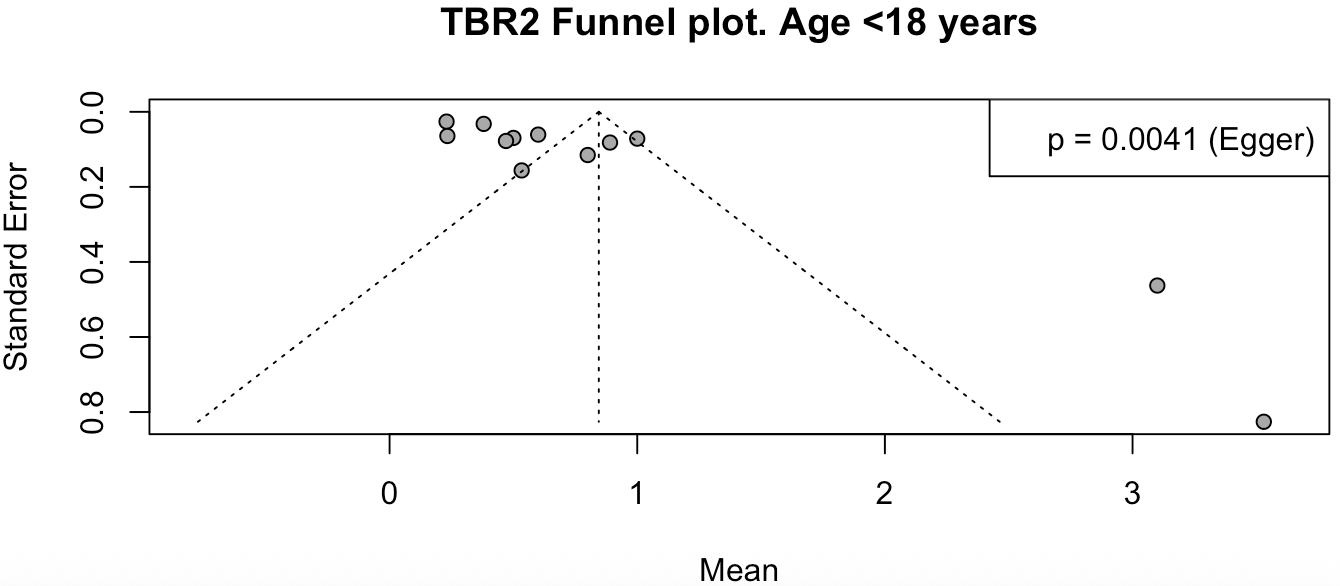

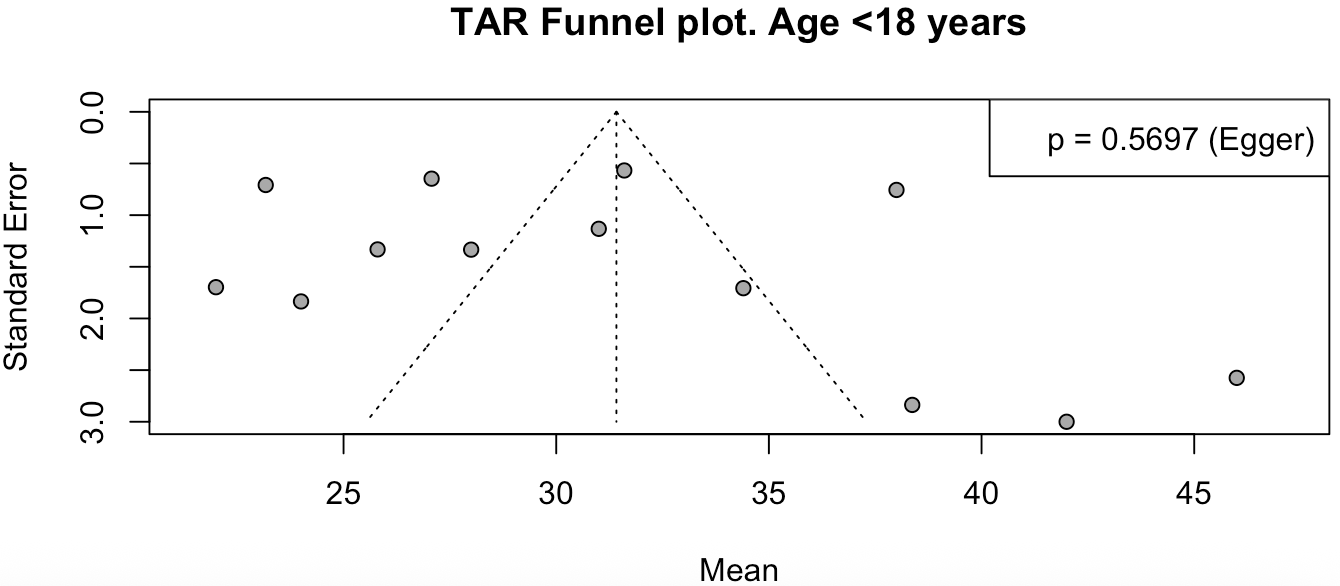

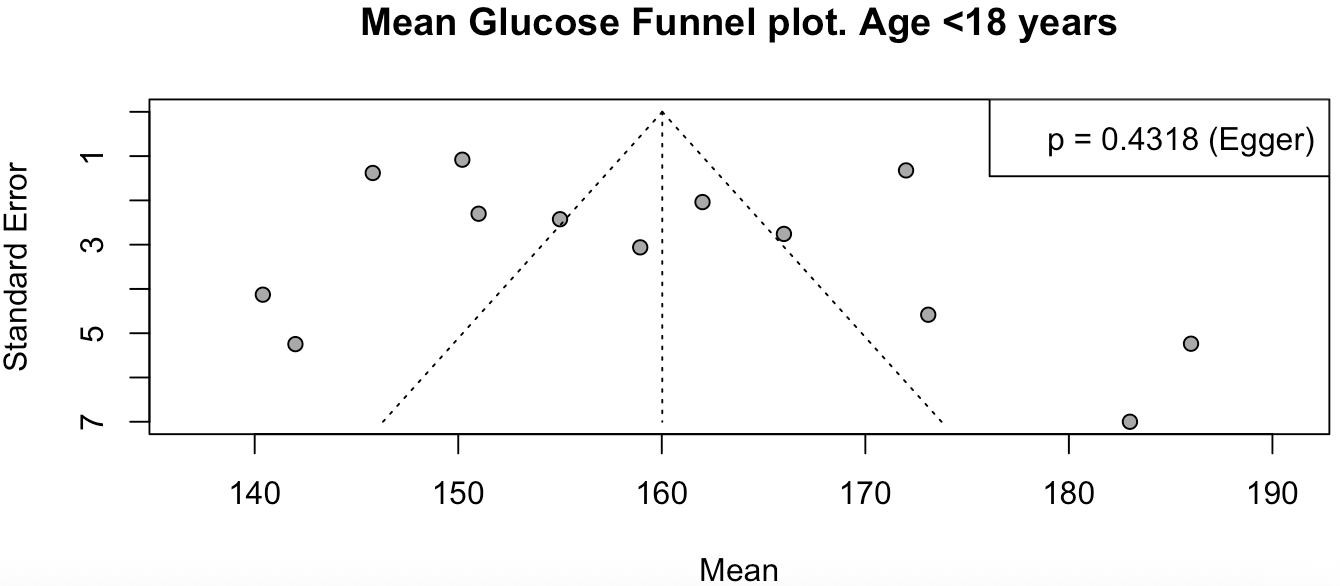

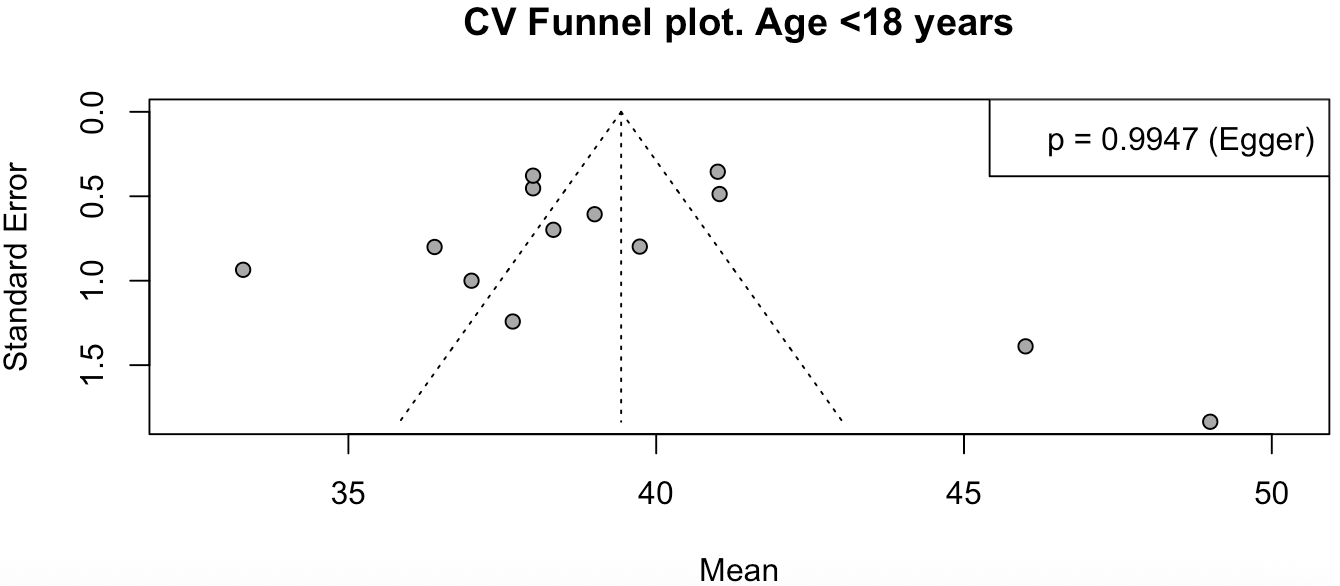

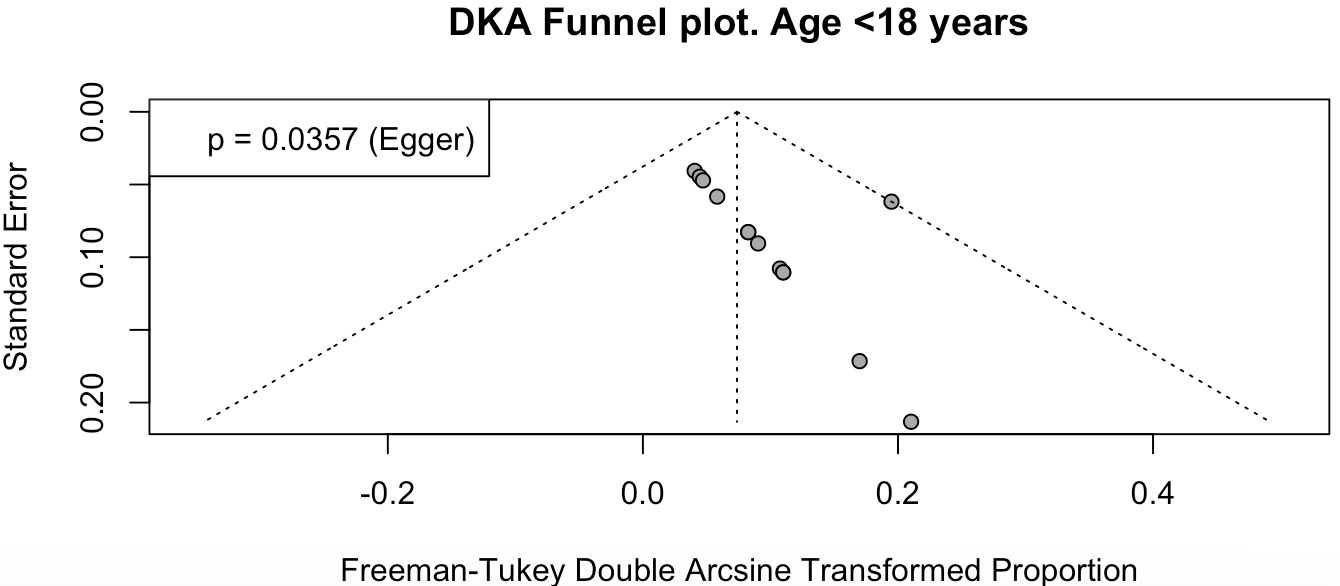
**

**
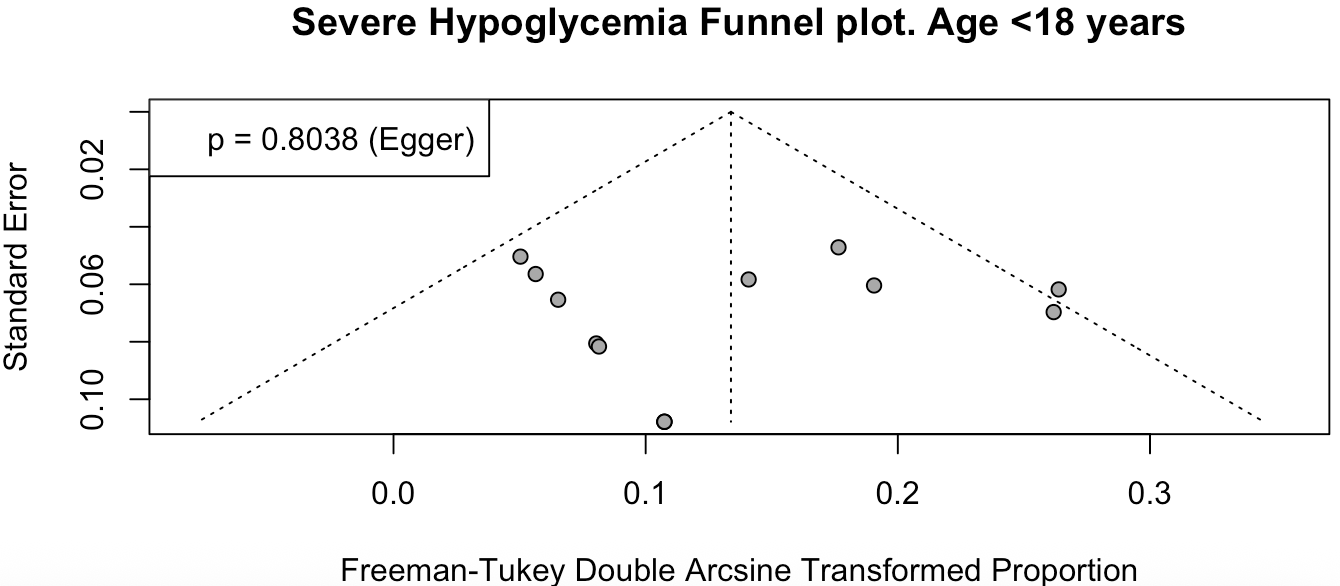

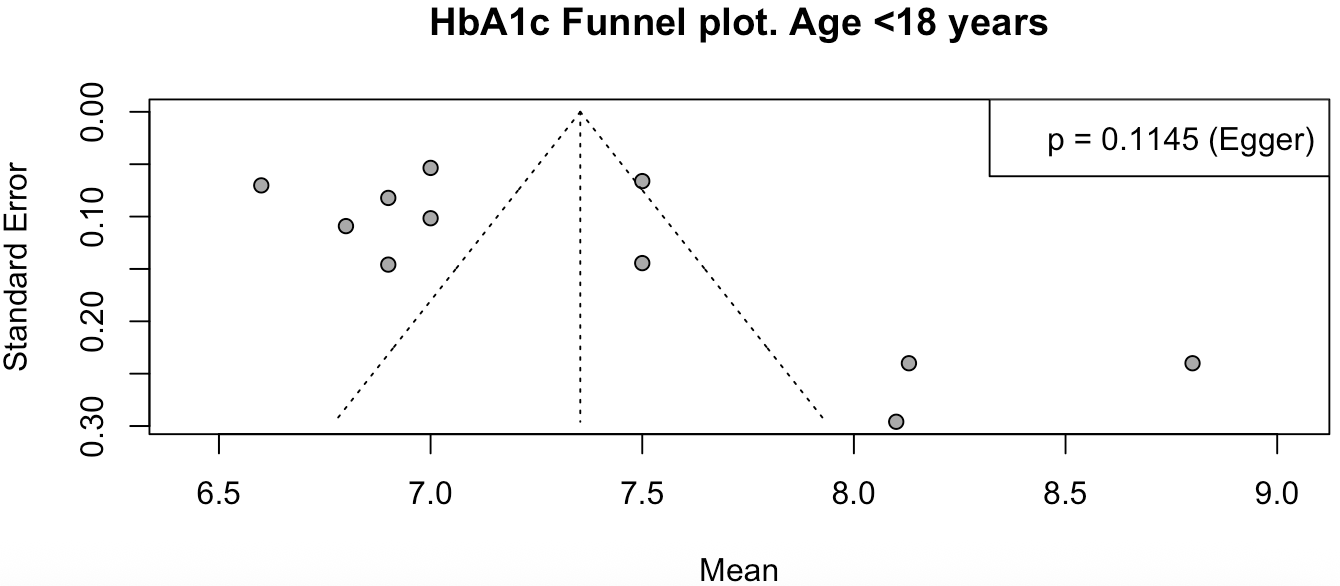
**

## 4.2 Studies with mean age ≥18 years

**
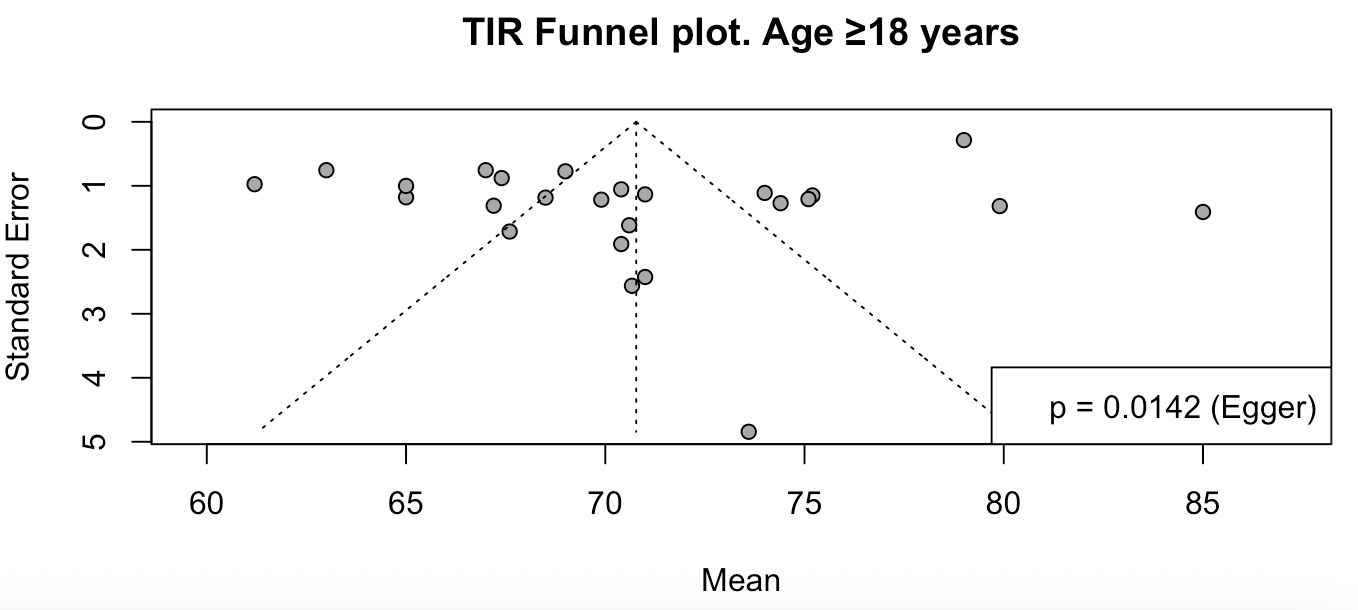
**

**
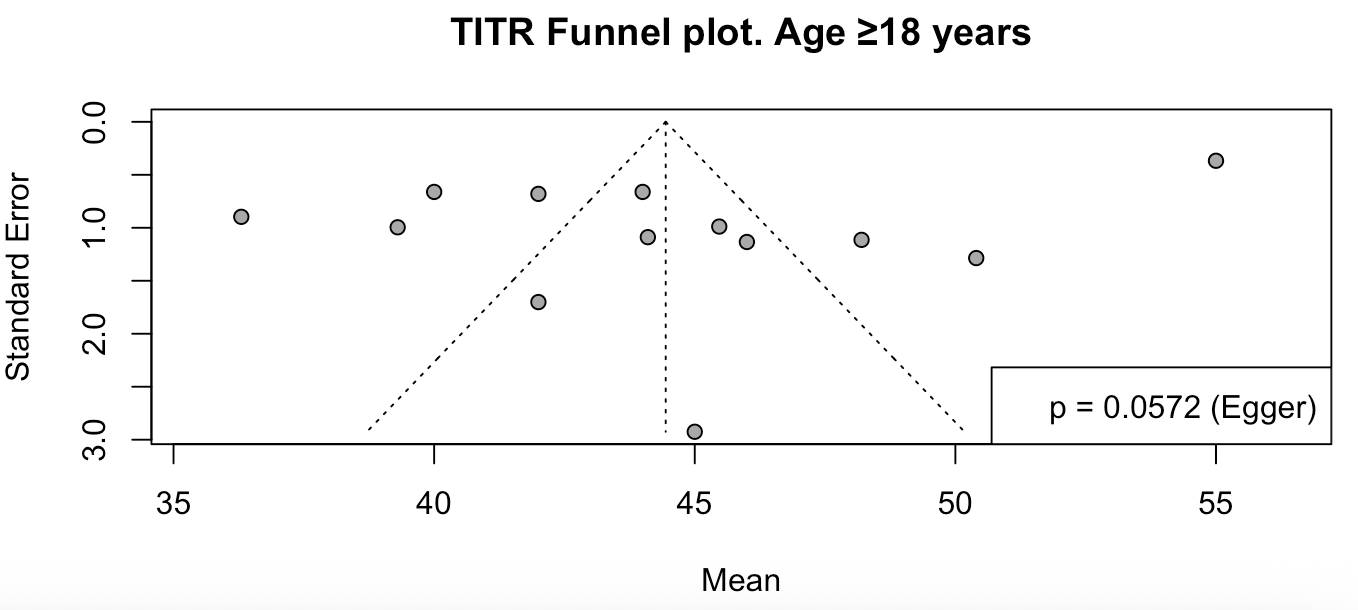

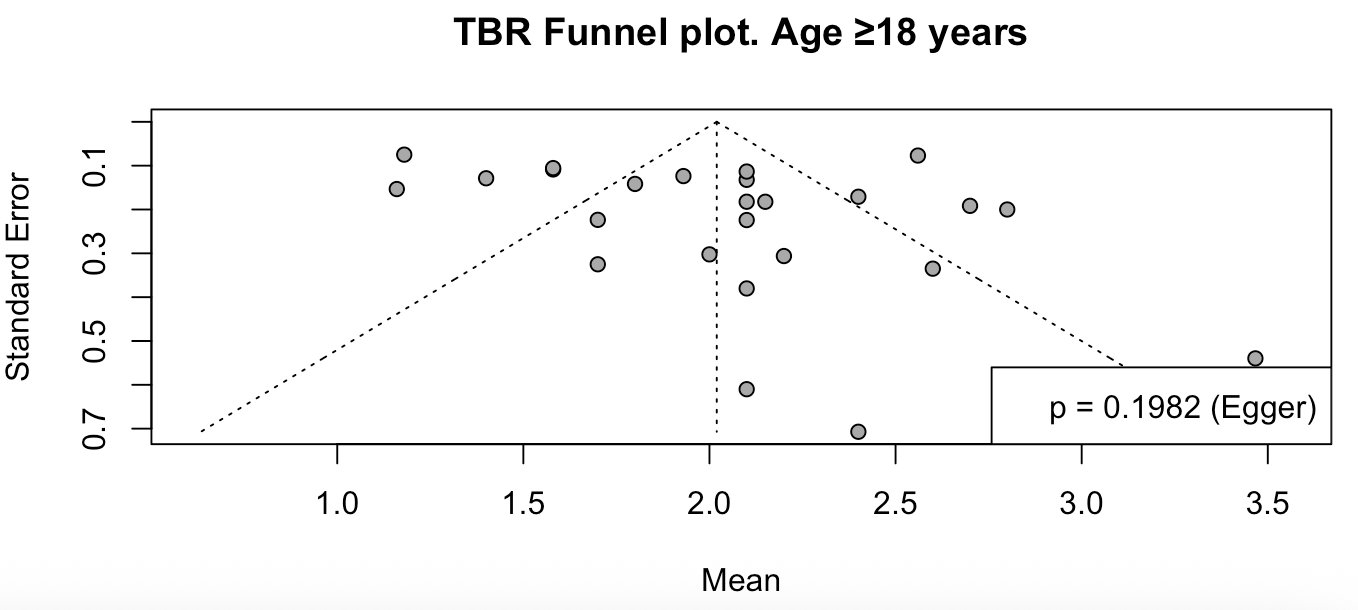

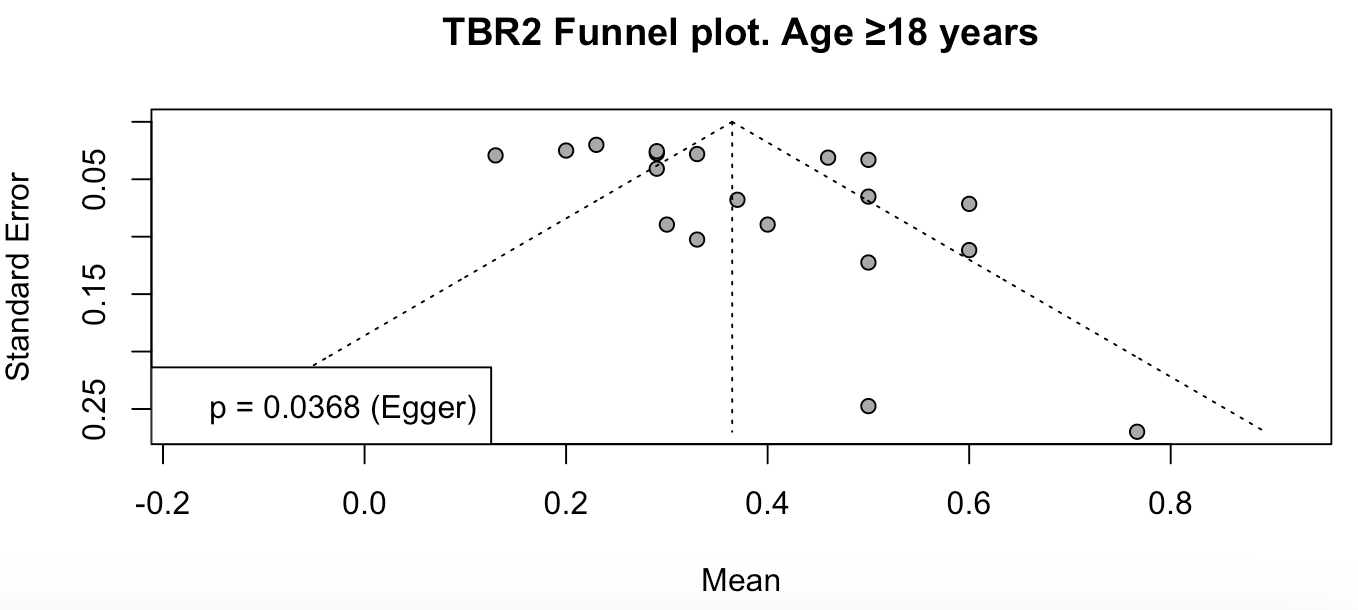

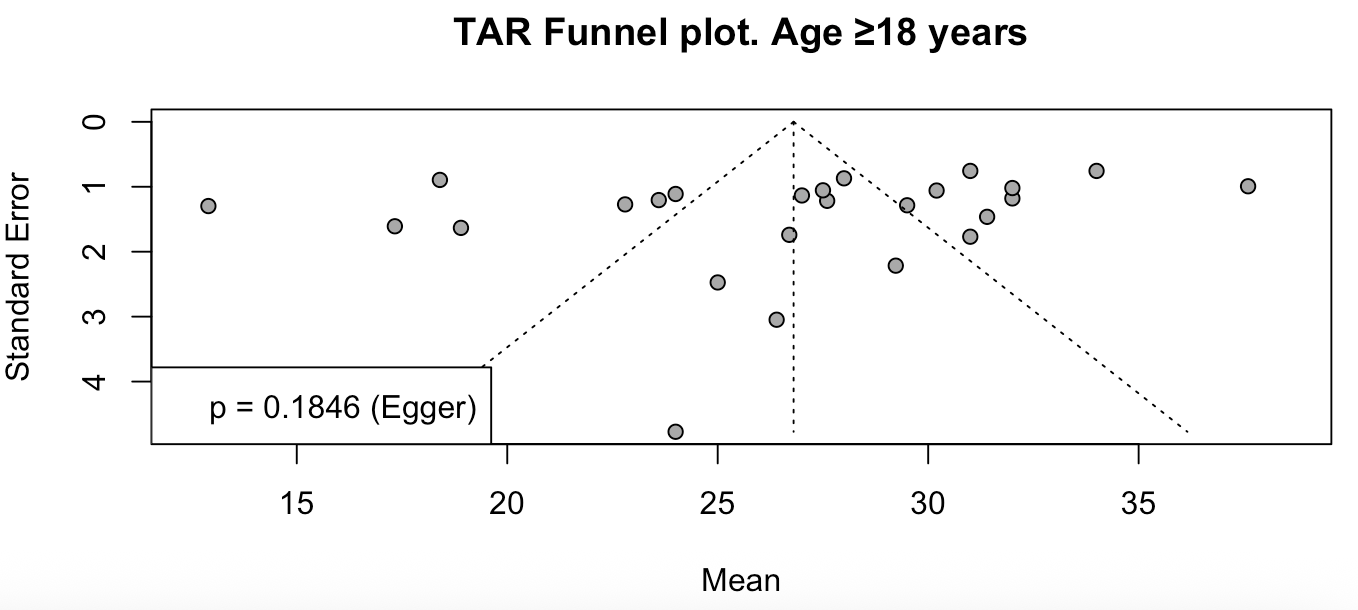

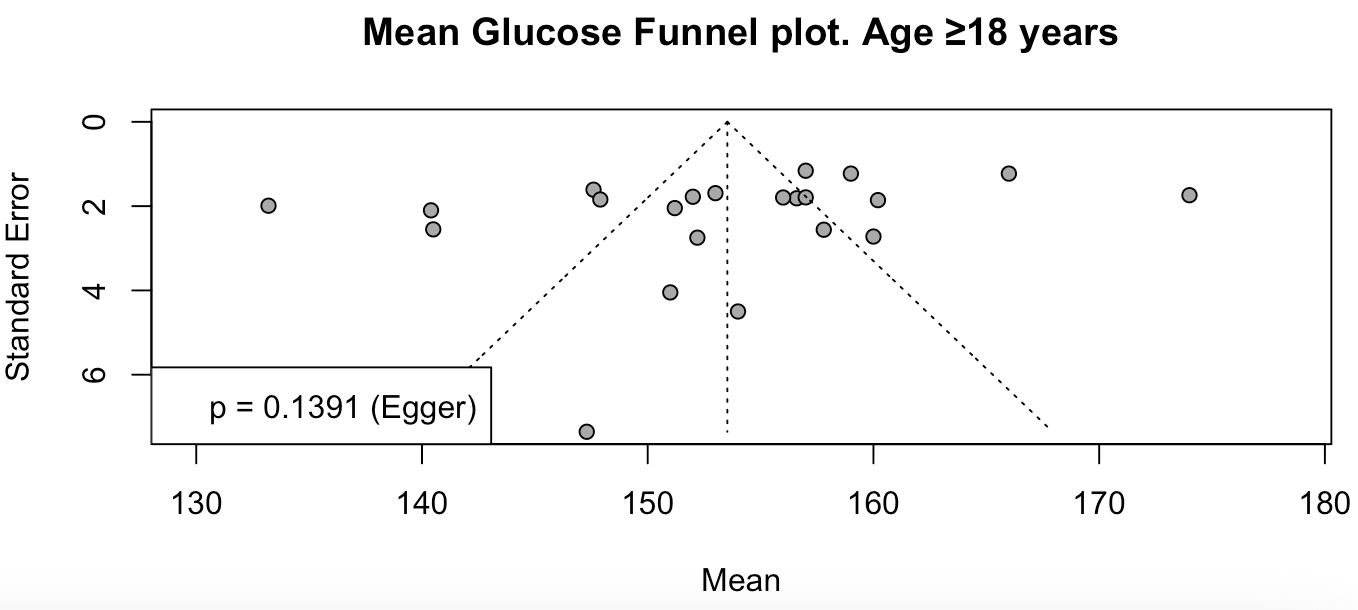
**

**
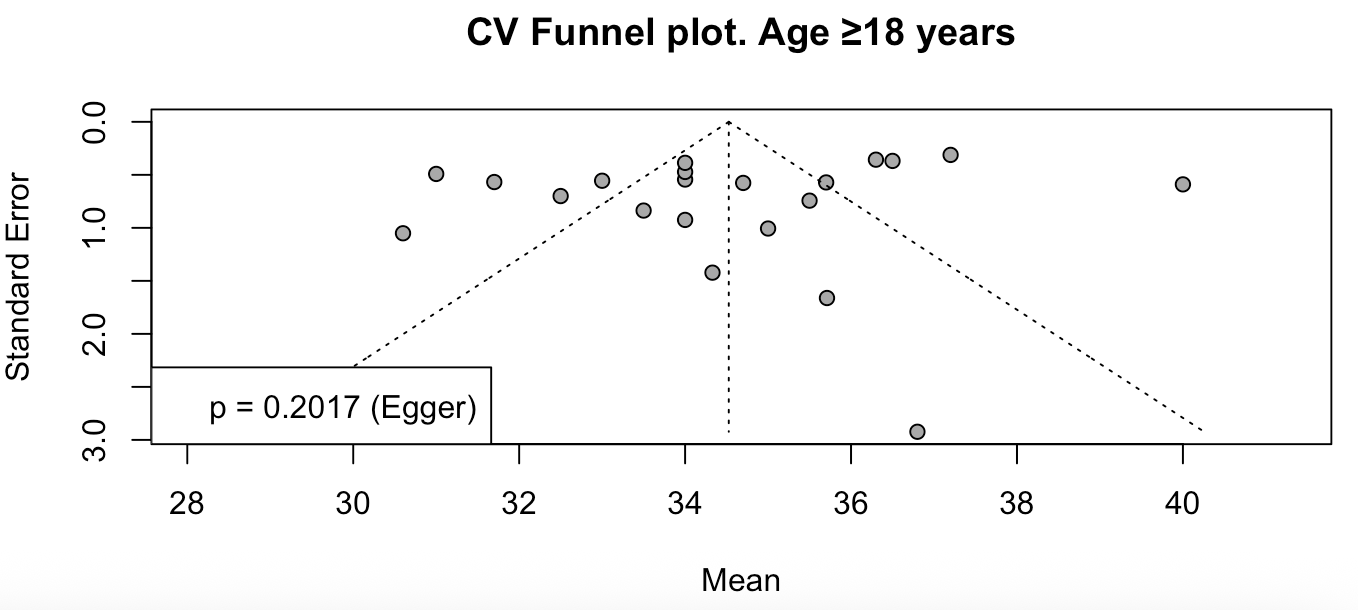
**

**
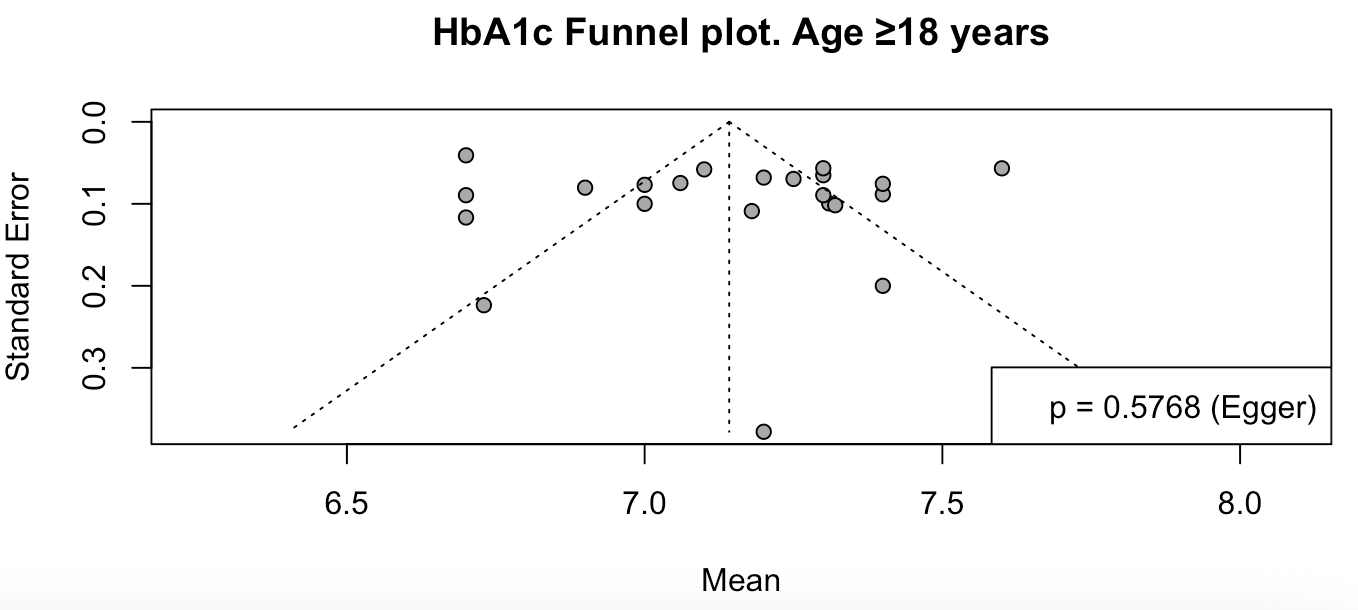
**

**
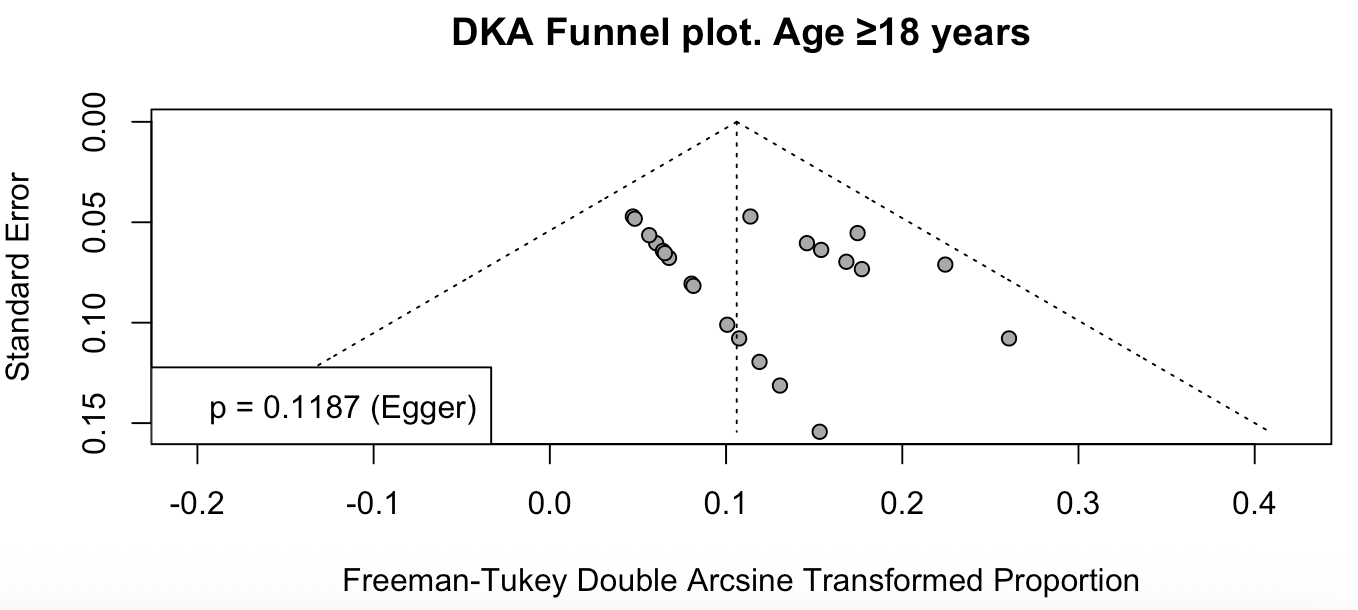
**

**
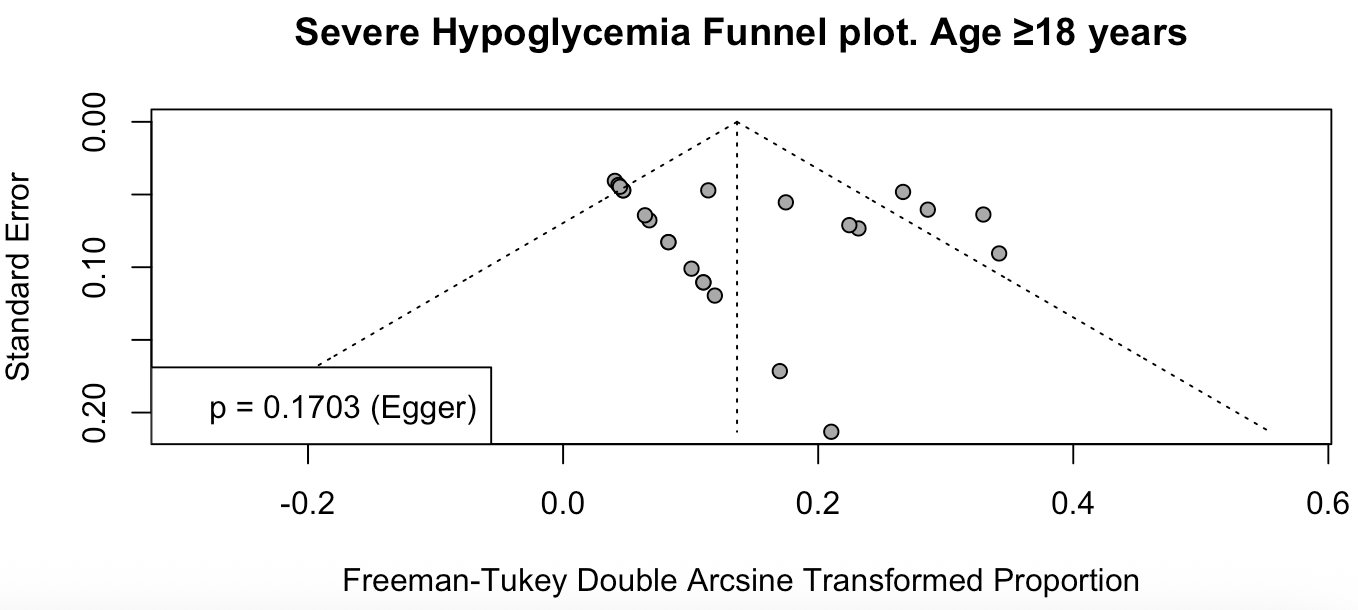
**

# Supplement 5. Forest plots

## 5.1 Studies with mean age <18 years


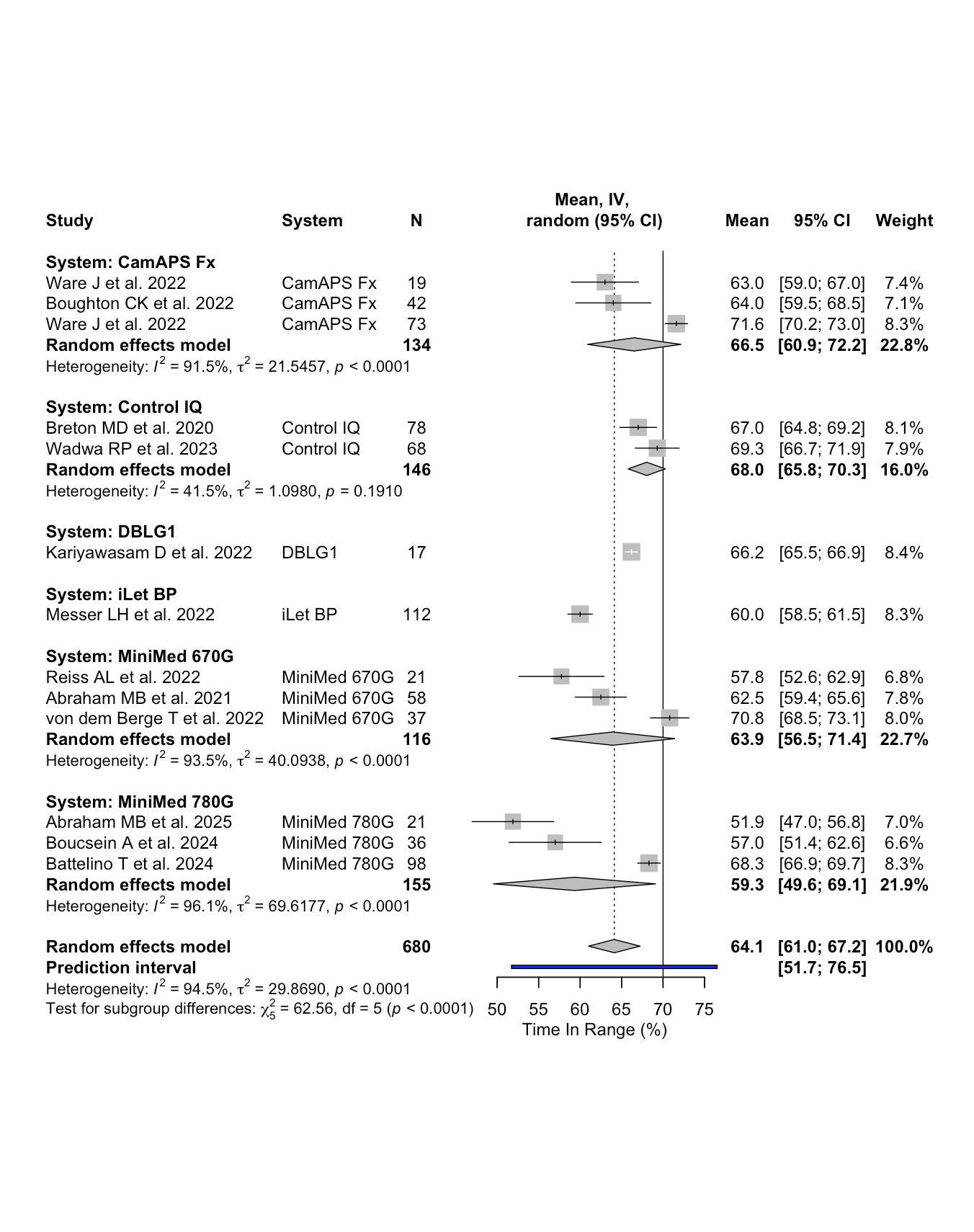


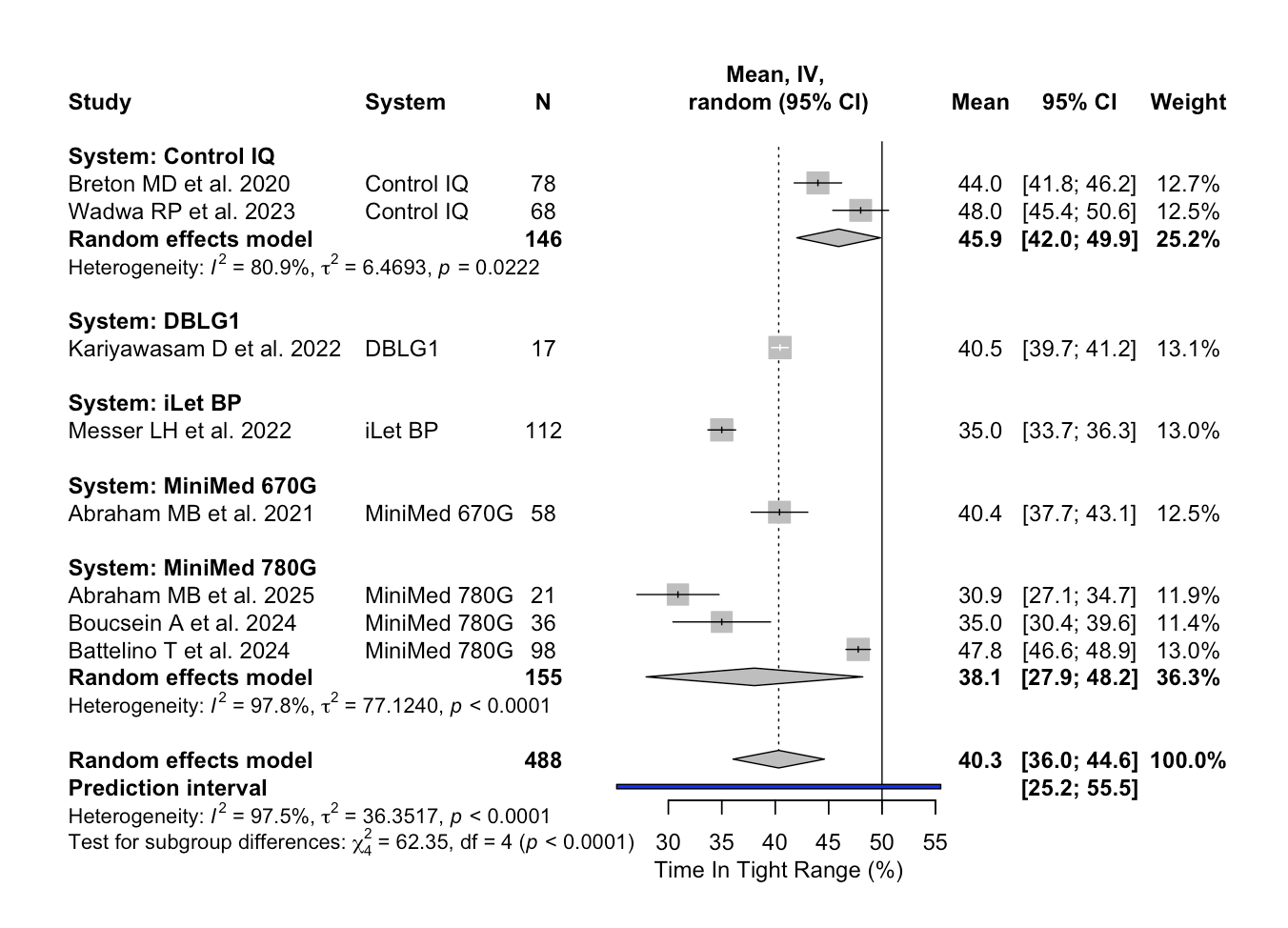


#
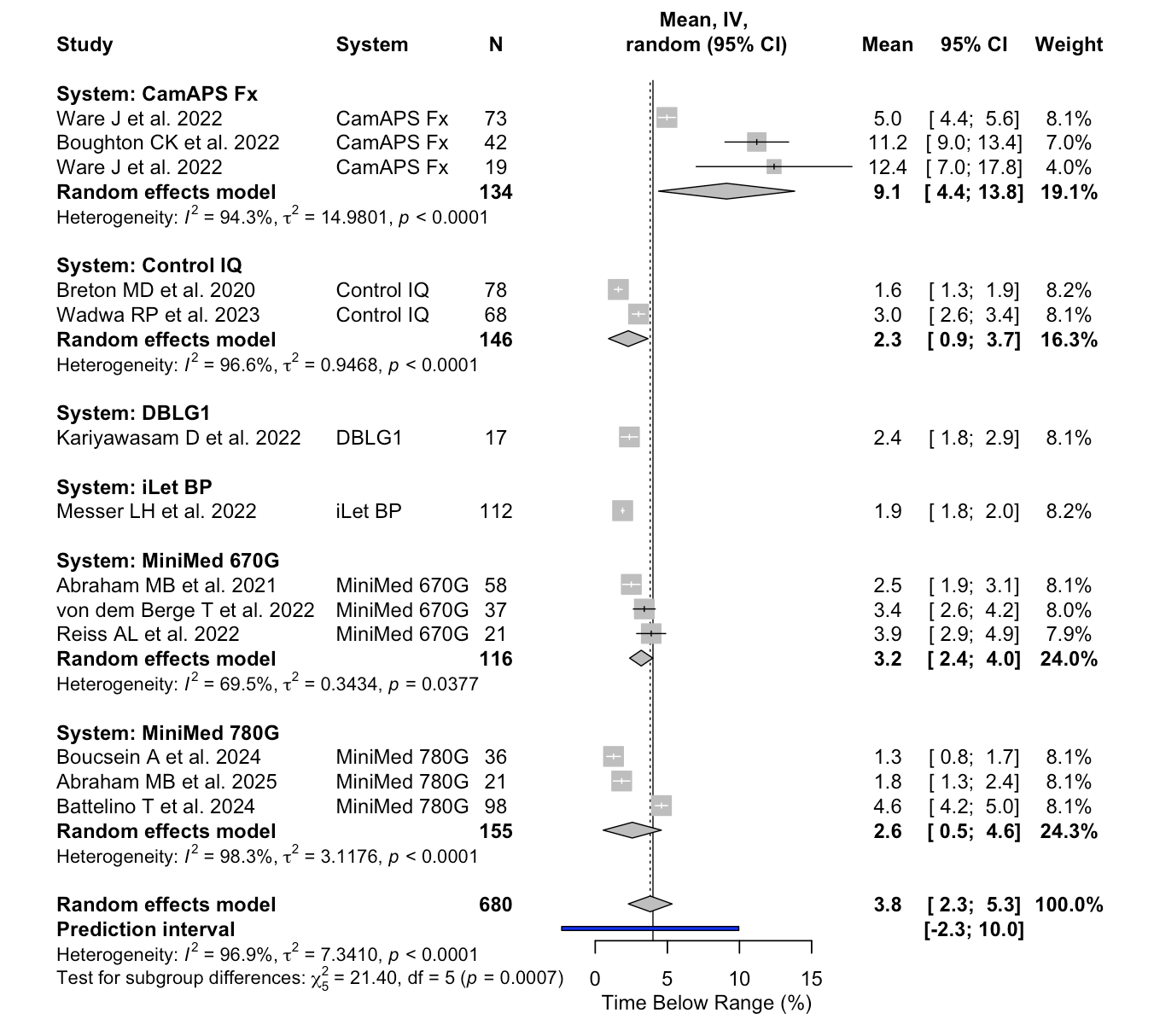

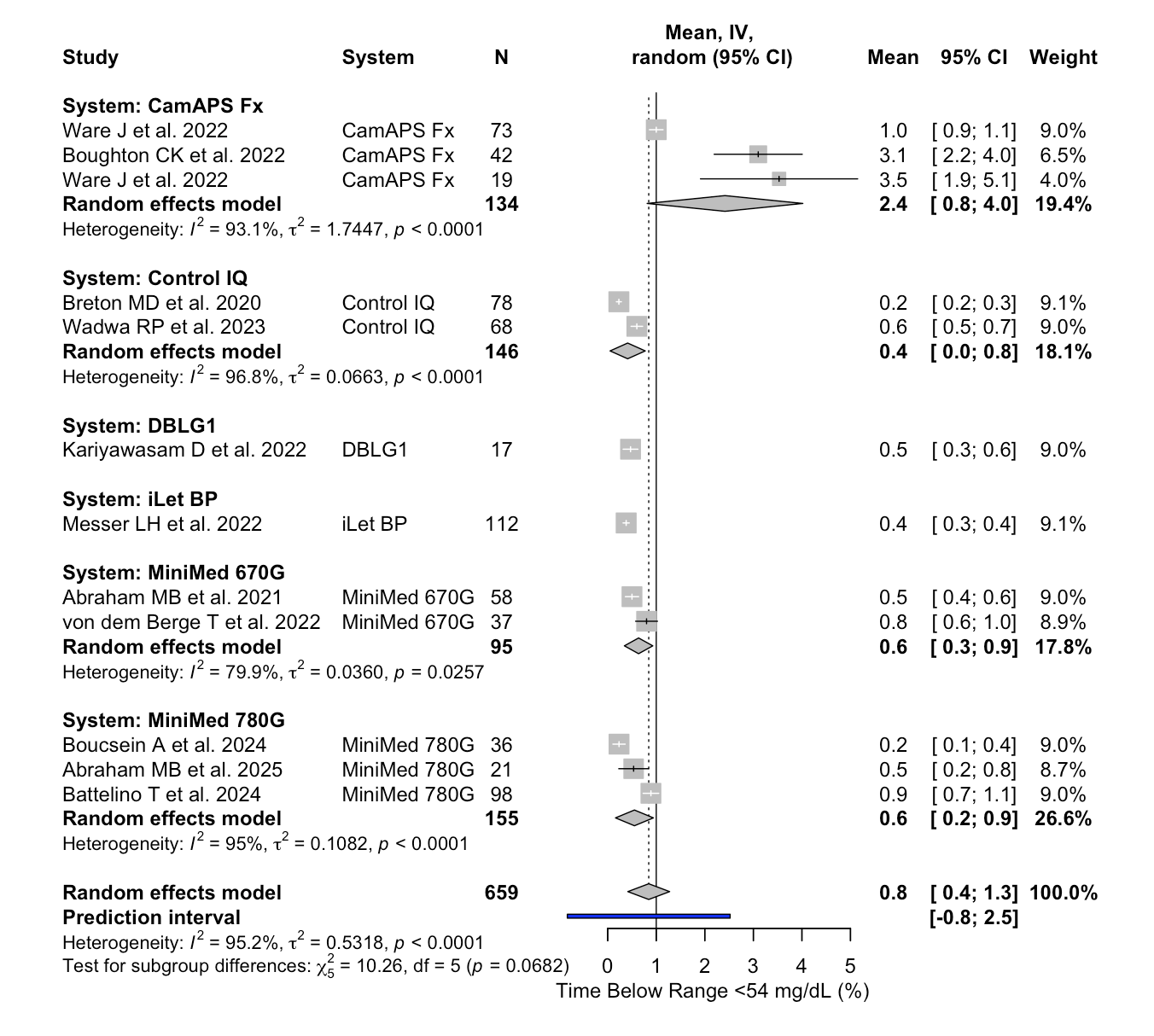

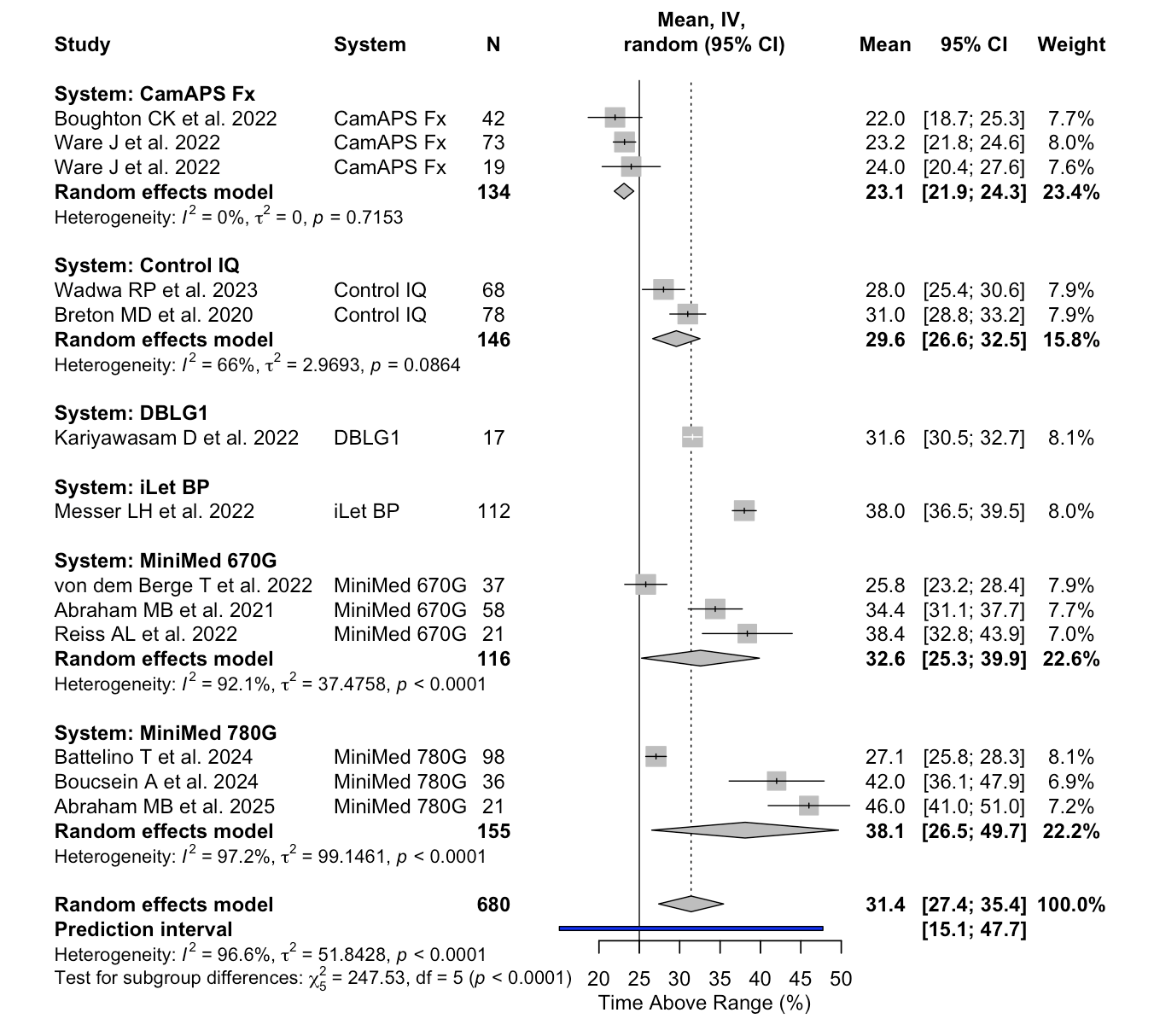

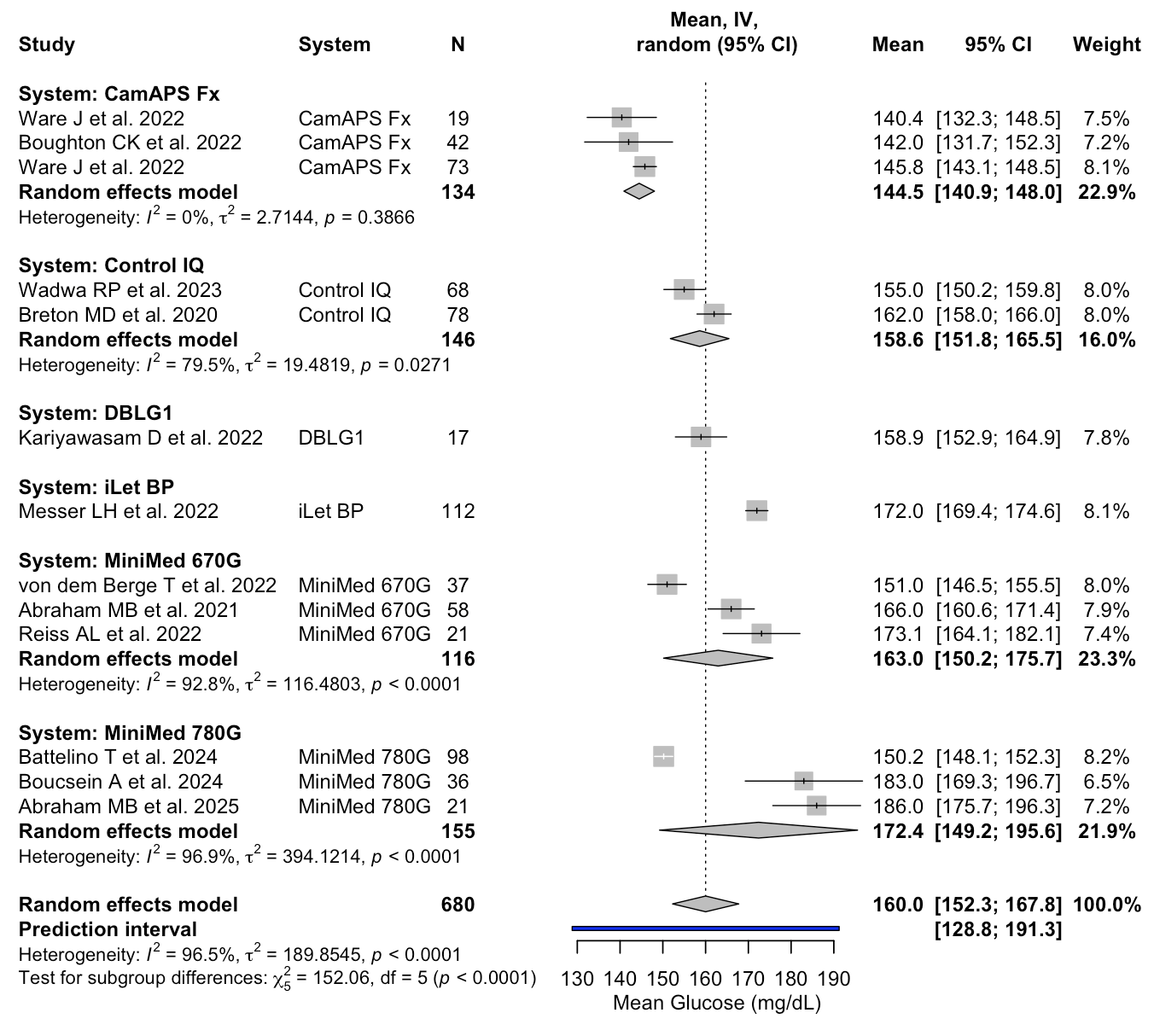

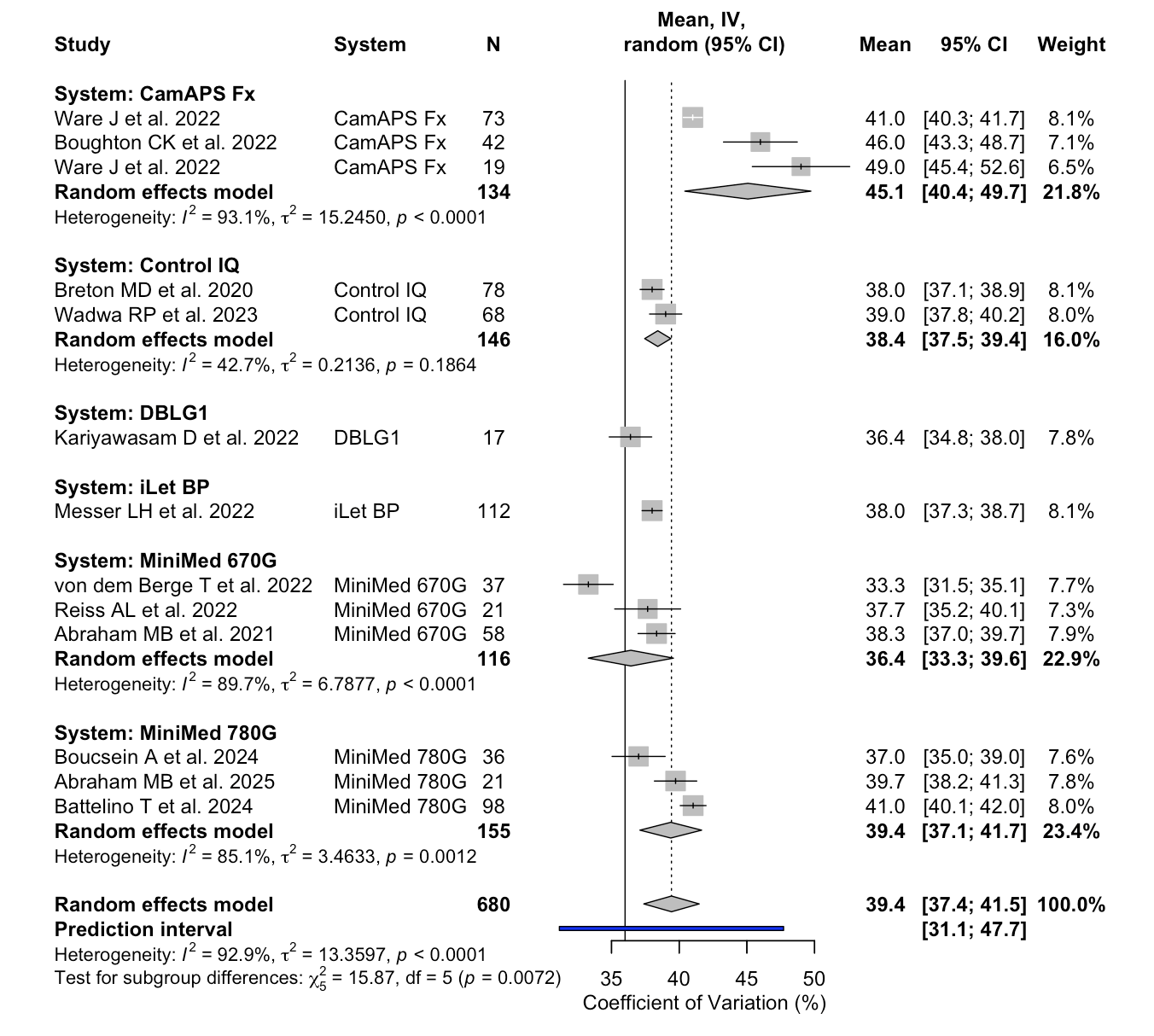

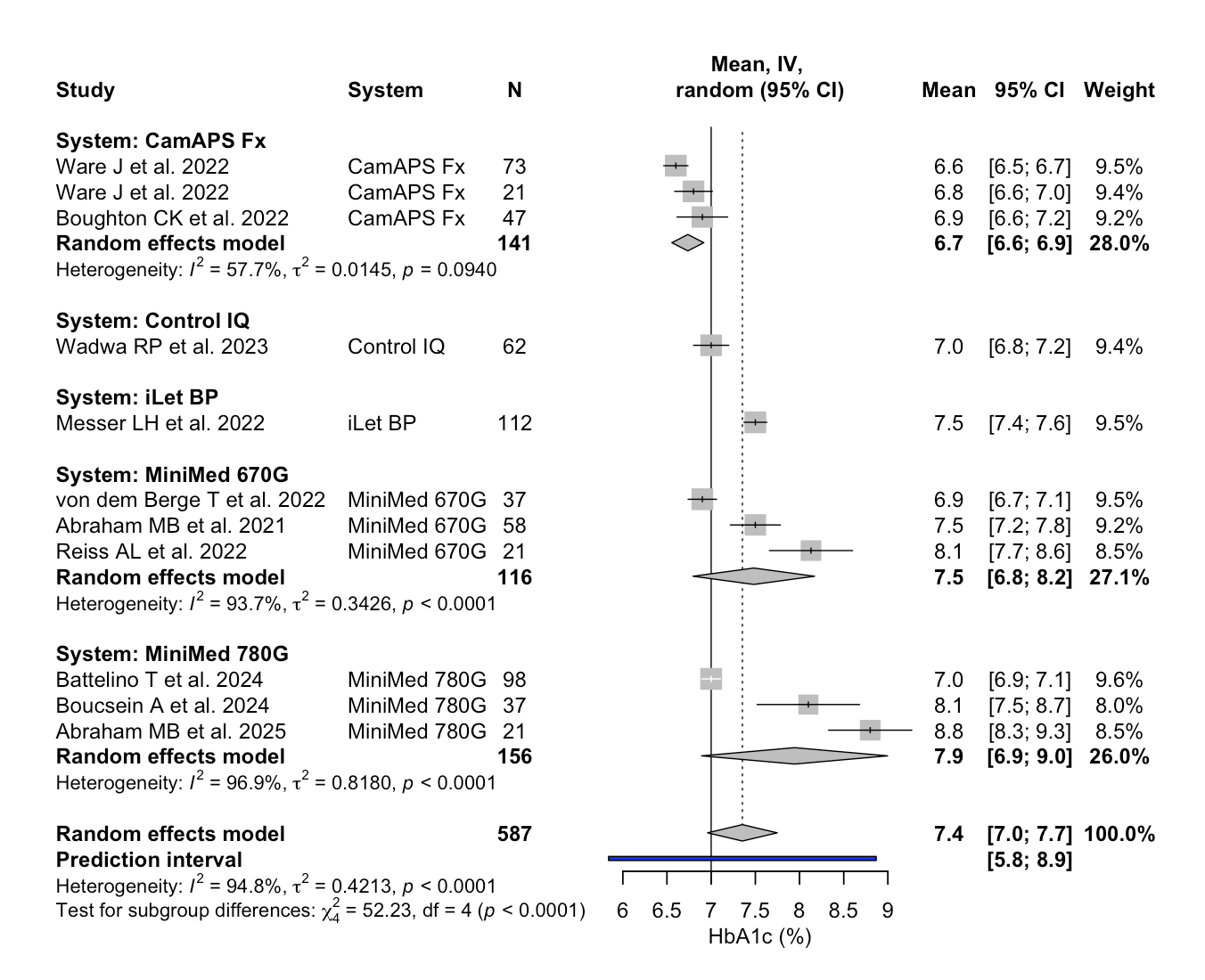


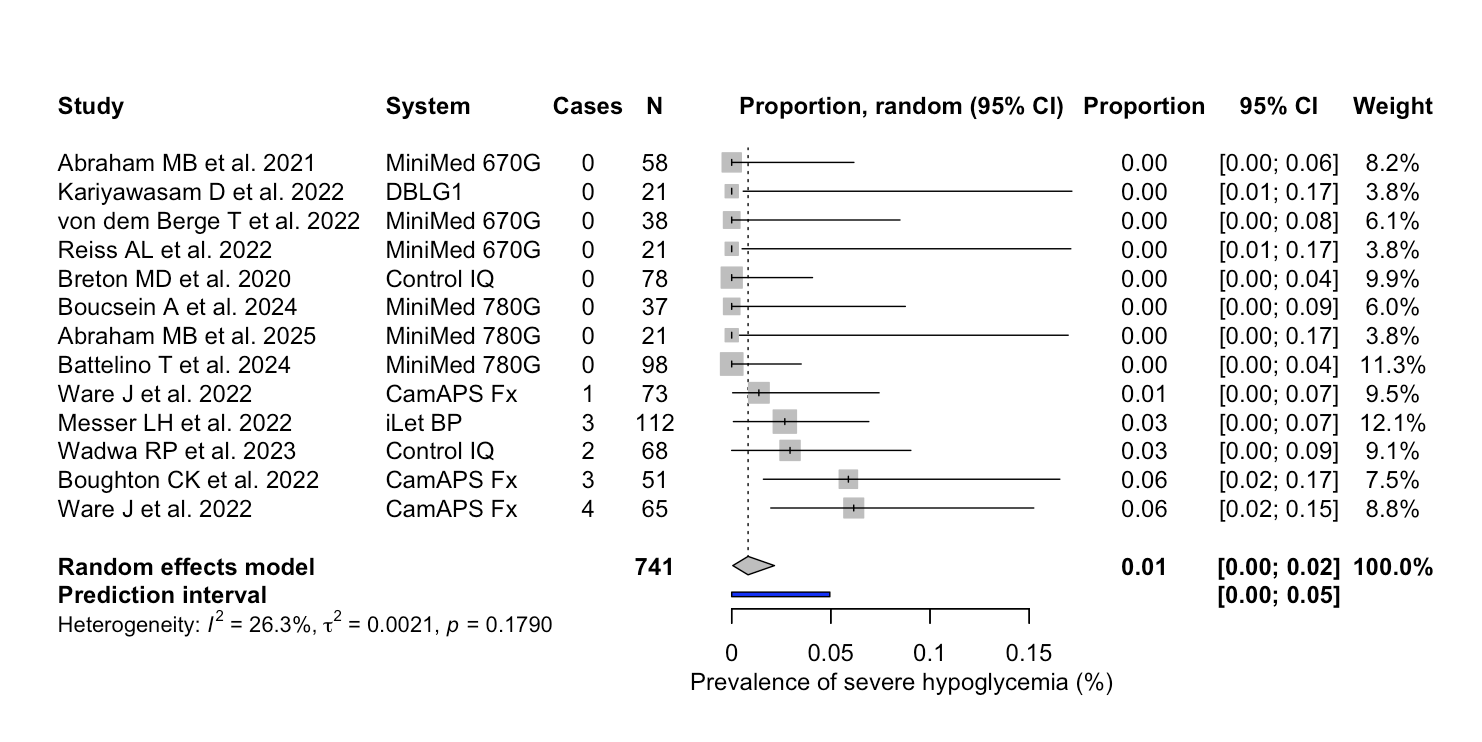

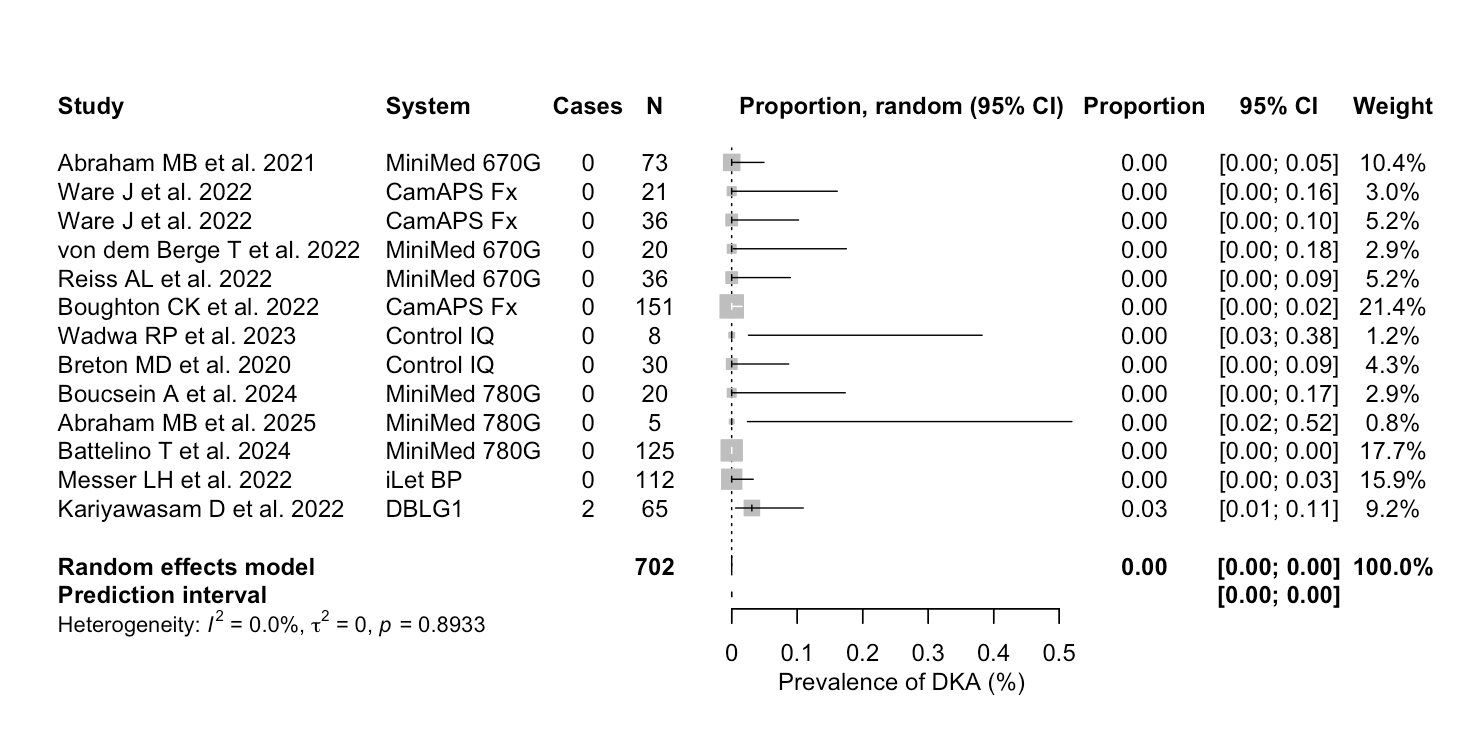
Each forest plot displays the pooled mean estimate (for continuous outcomes) or pooled mean proportion (for binary outcomes) with corresponding 95% confidence intervals for each study, based on random-effects meta-analyses. The overall pooled estimate and its prediction interval are reported at the bottom of each plot. Measures of heterogeneity and subgroup differences are also provided when applicable. The dashed vertical line represents the overall pooled effect estimate, while the solid vertical line (when shown) corresponds to reference thresholds derived from international consensus statements or clinical guidelines. These thresholds are available for Time in Range (TIR), Time Below Range (TBR and TBR <54 mg/dL), Time Above Range (TAR), coefficient of variation (CV), and HbA1c. No specific reference values were applied for mean glucose, severe hypoglycemia, or diabetic ketoacidosis (DKA).

## 5.2 Studies with mean age ≥18 years

#

**
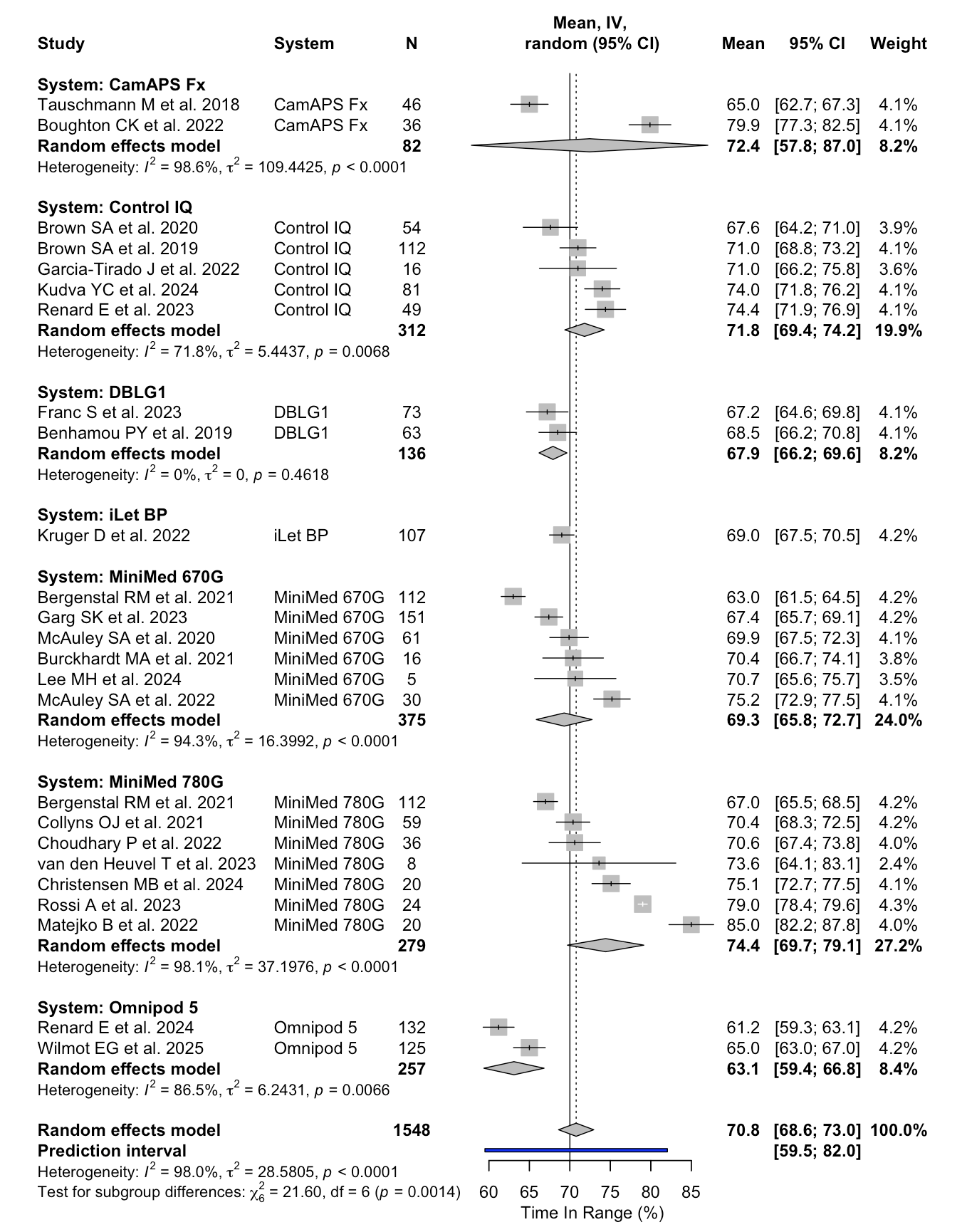
**

**
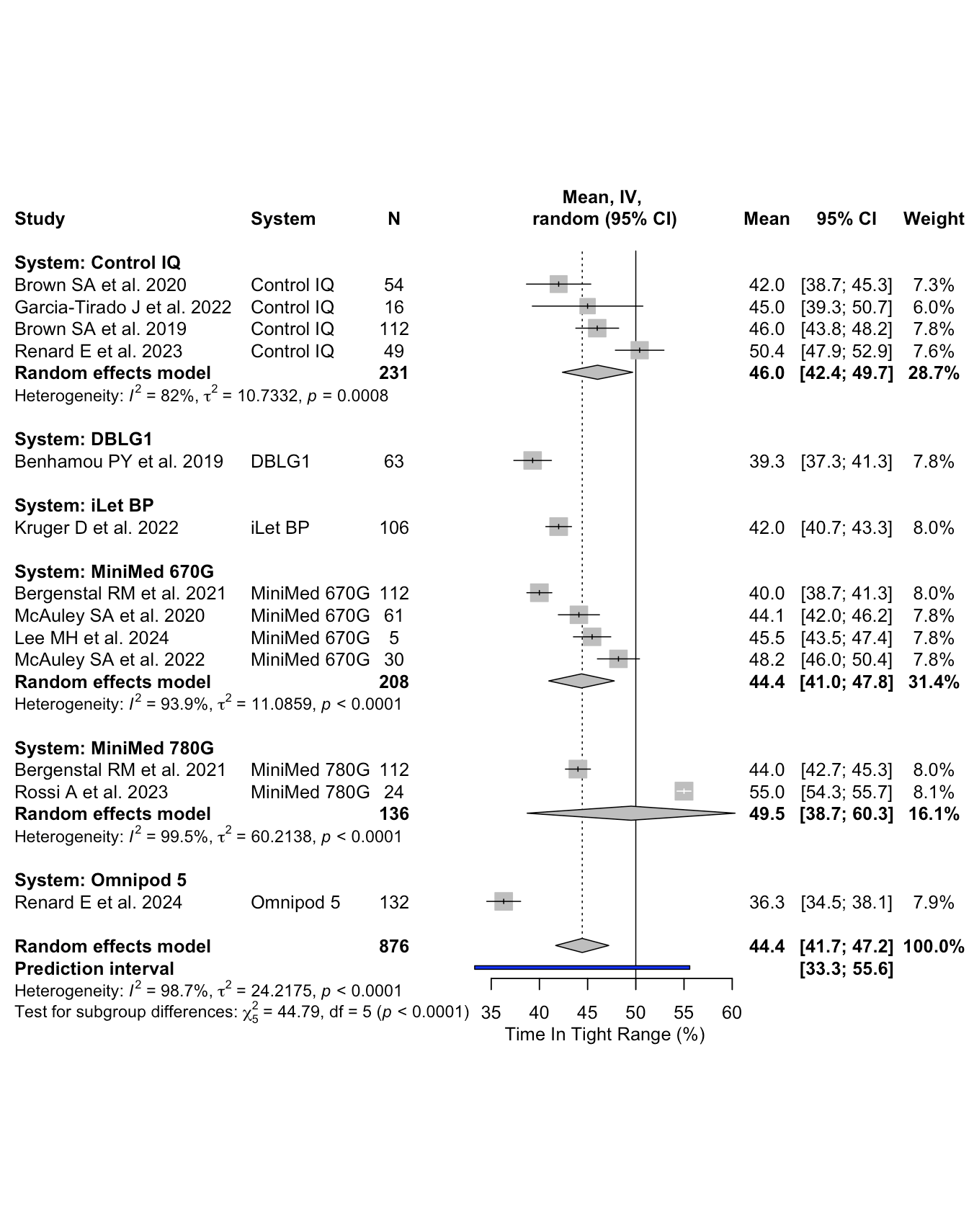

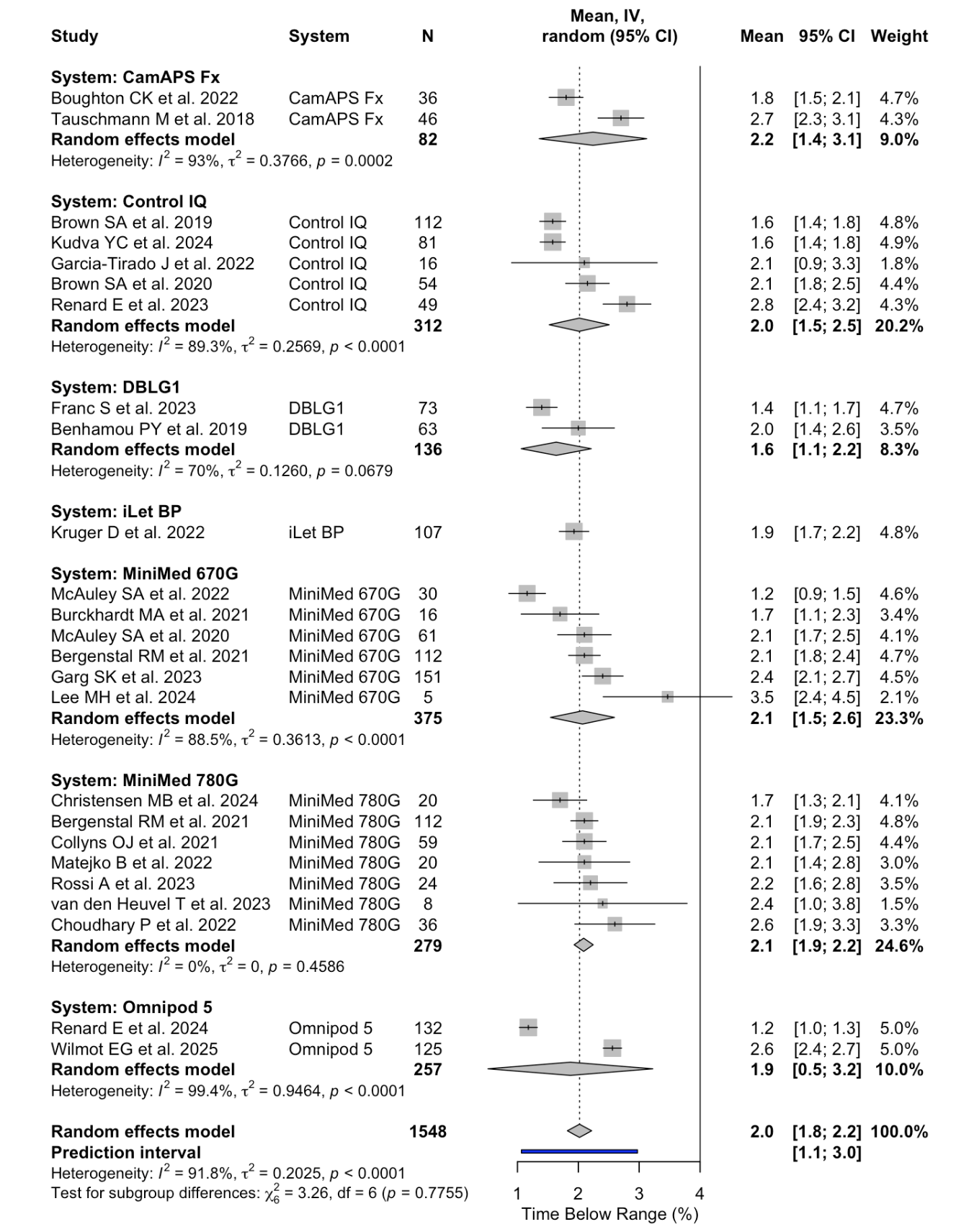

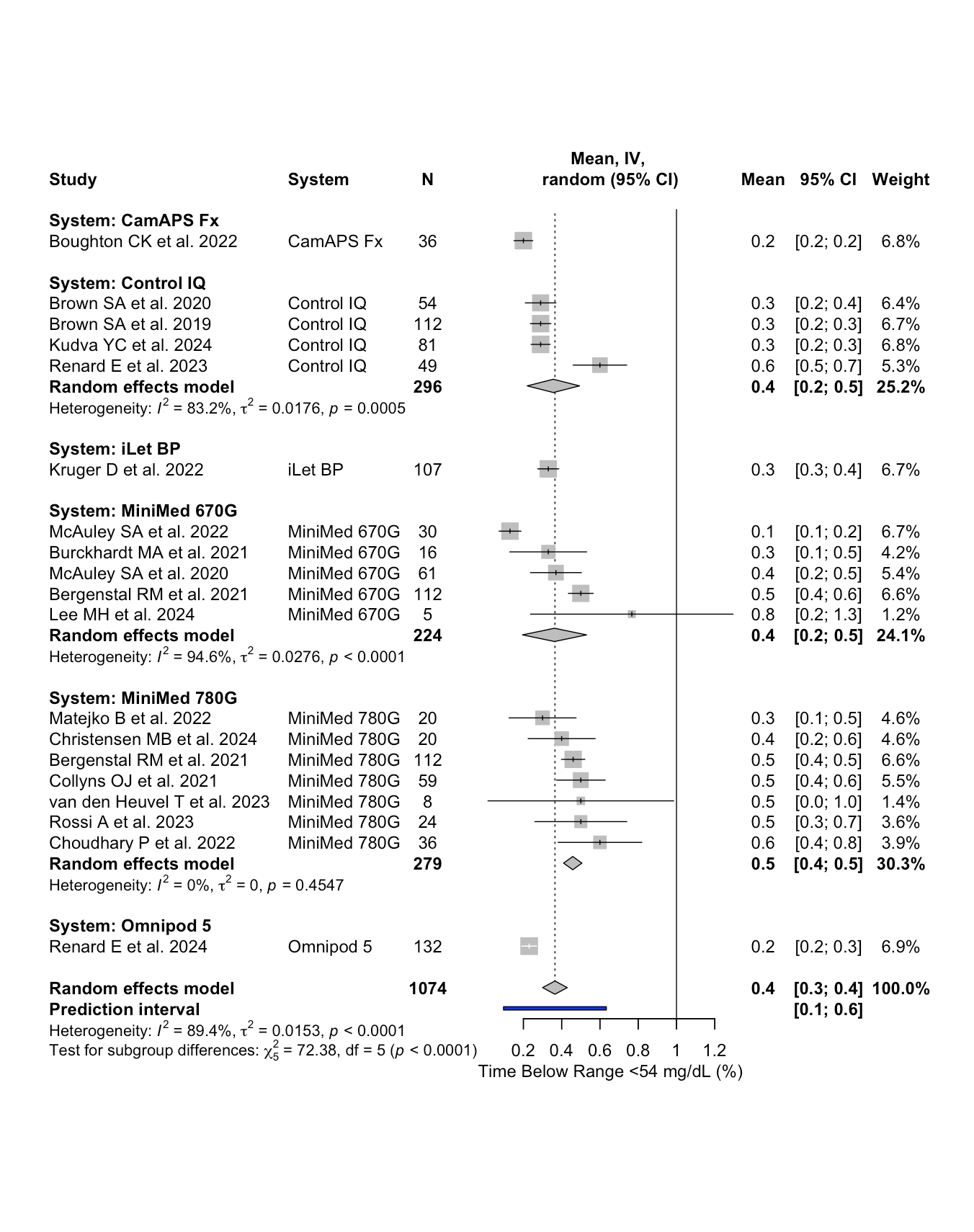

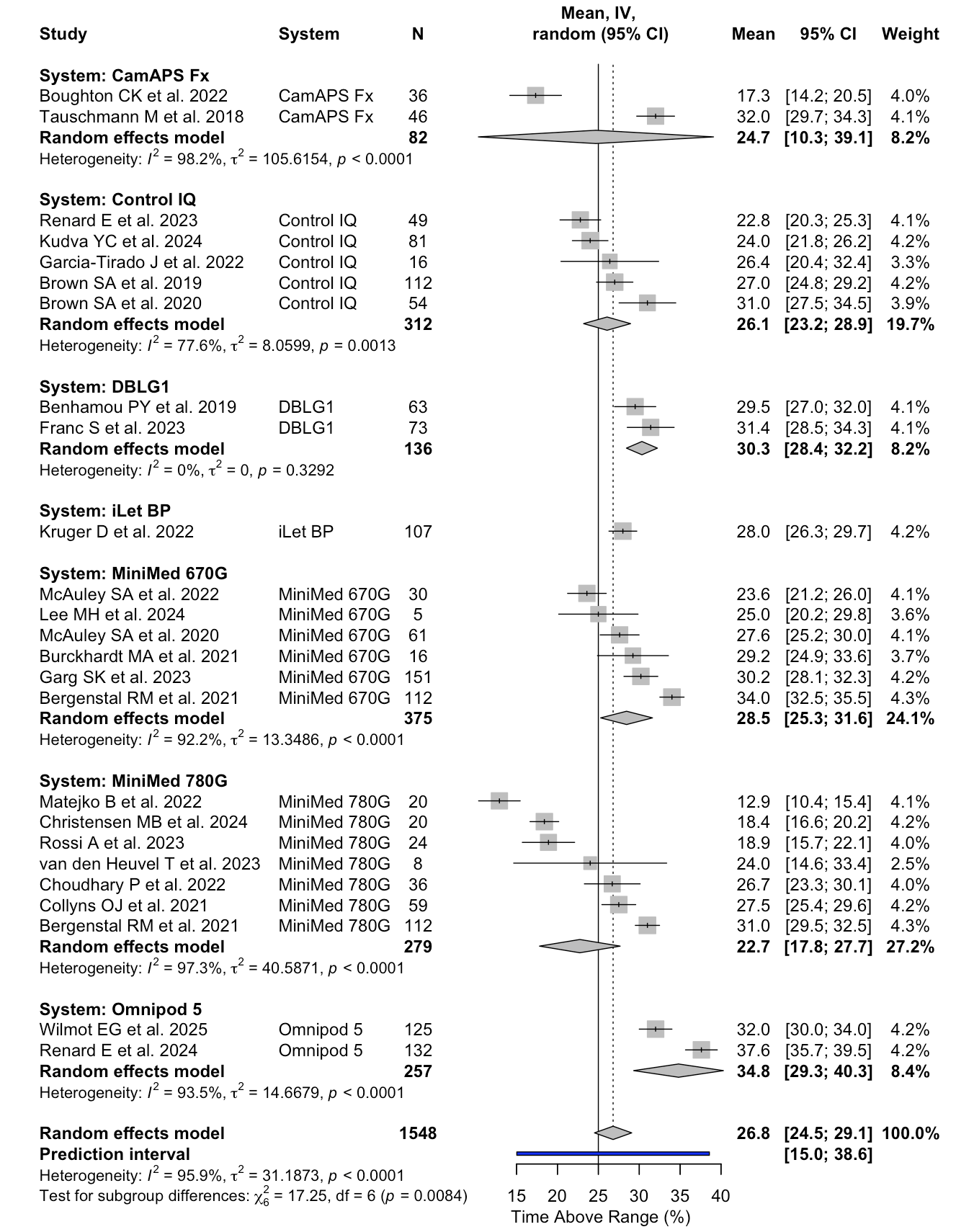

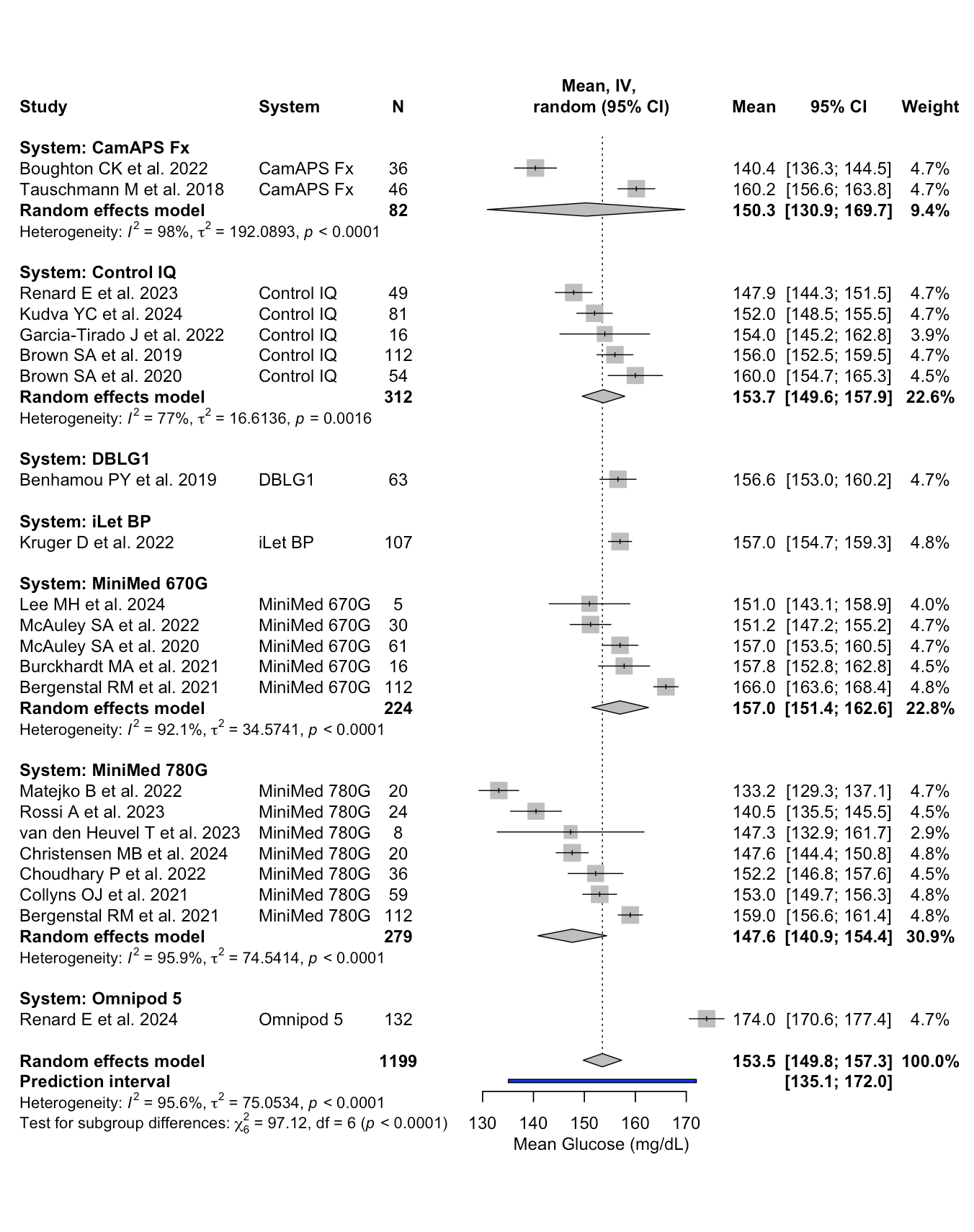

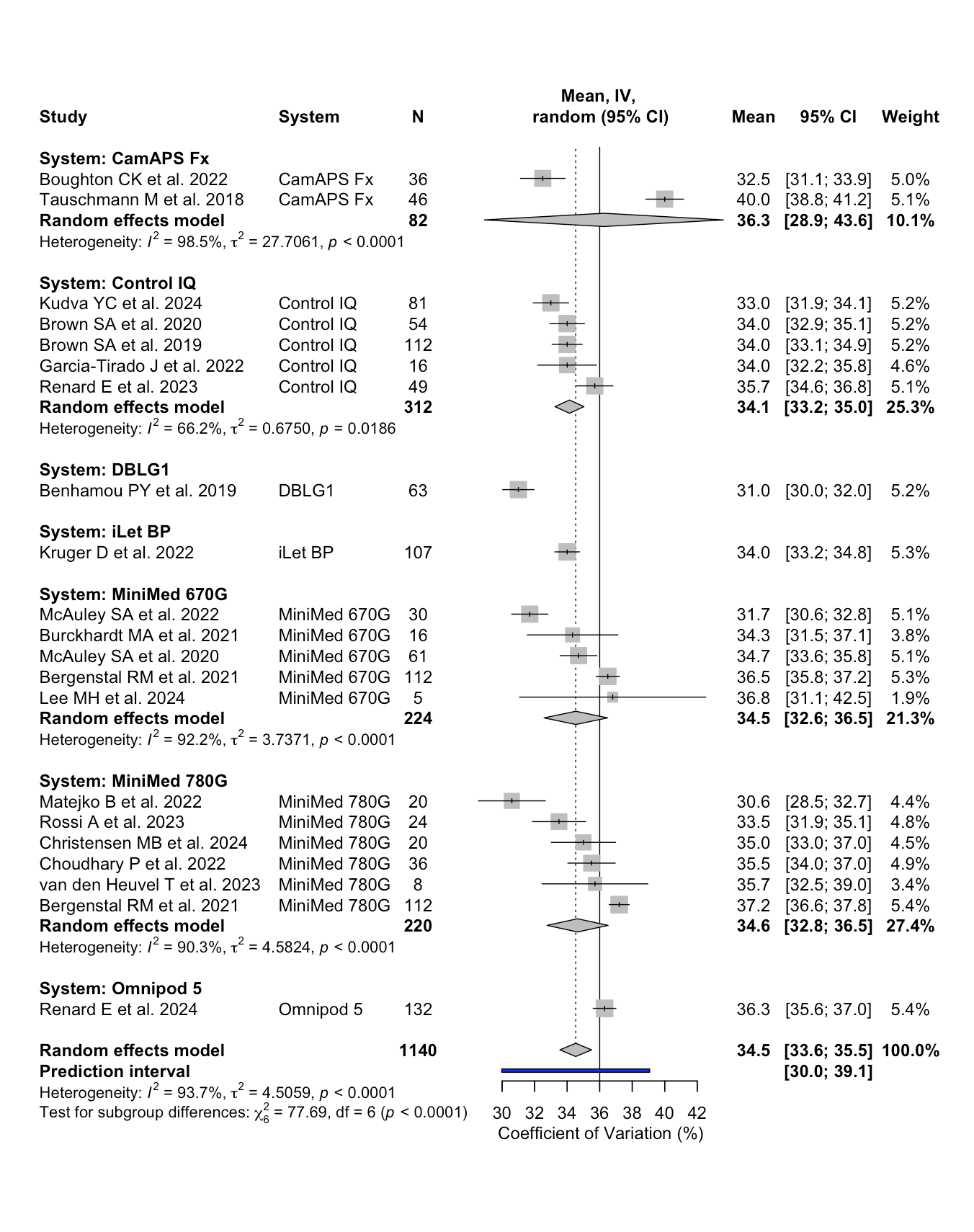

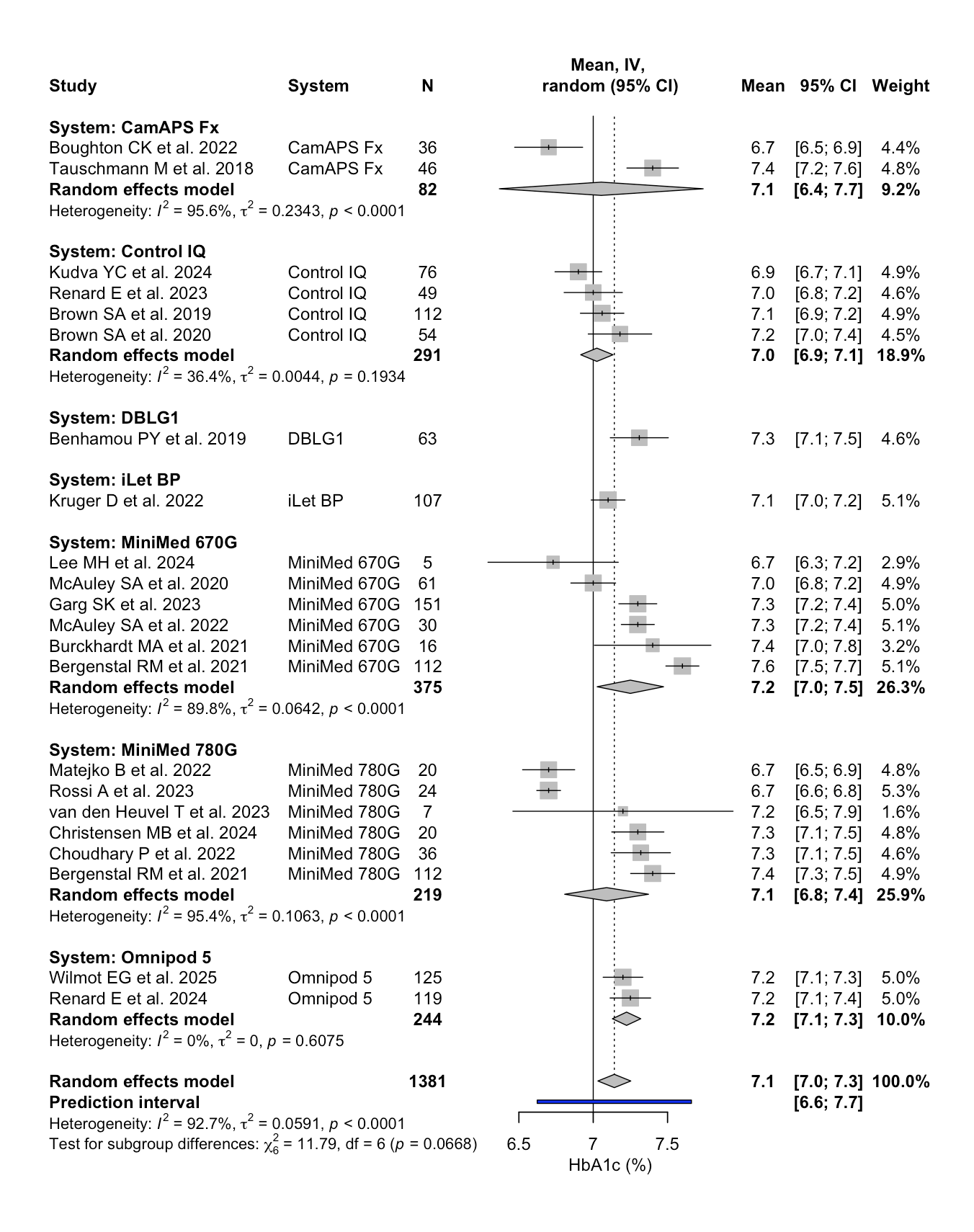

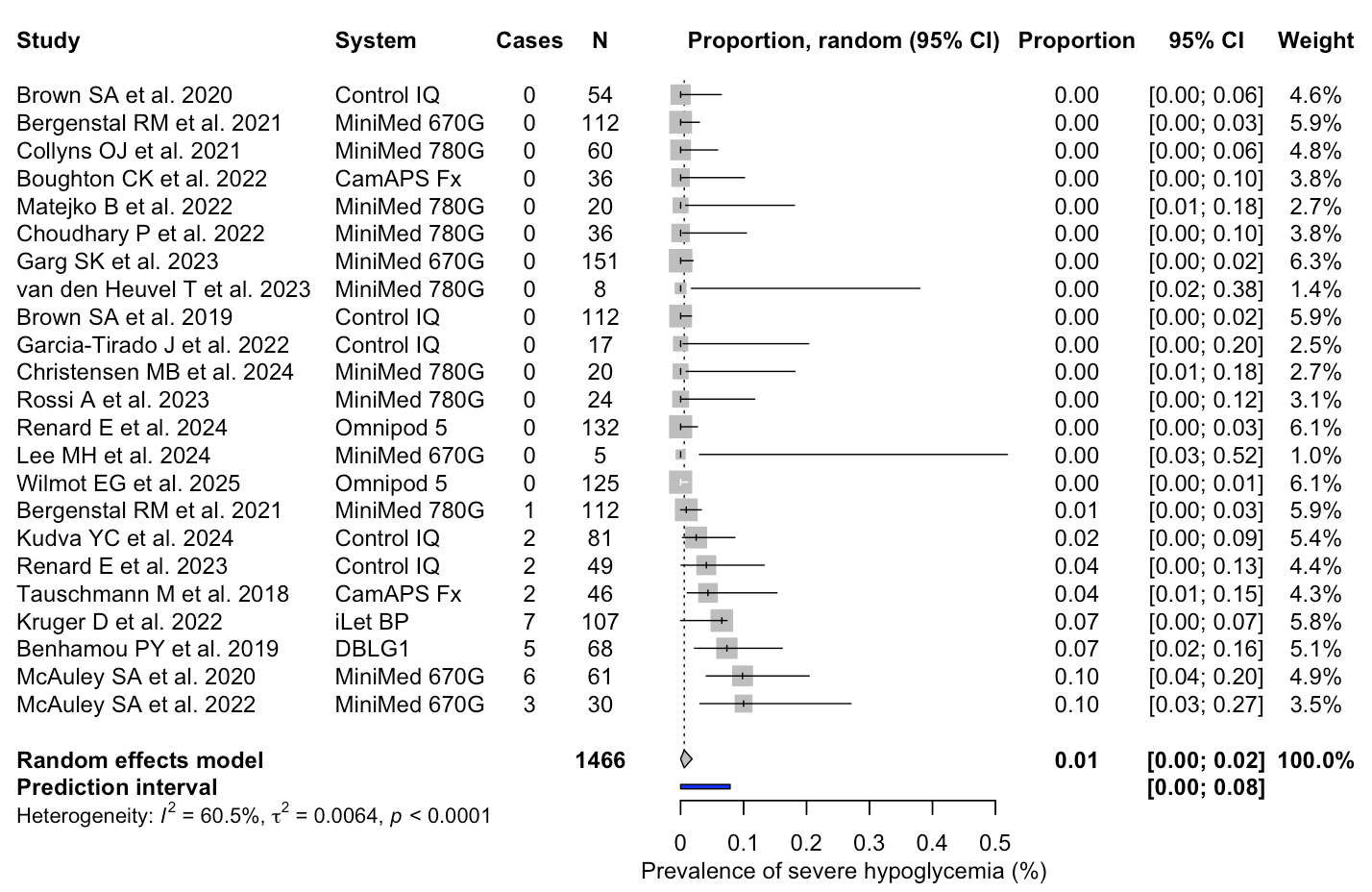

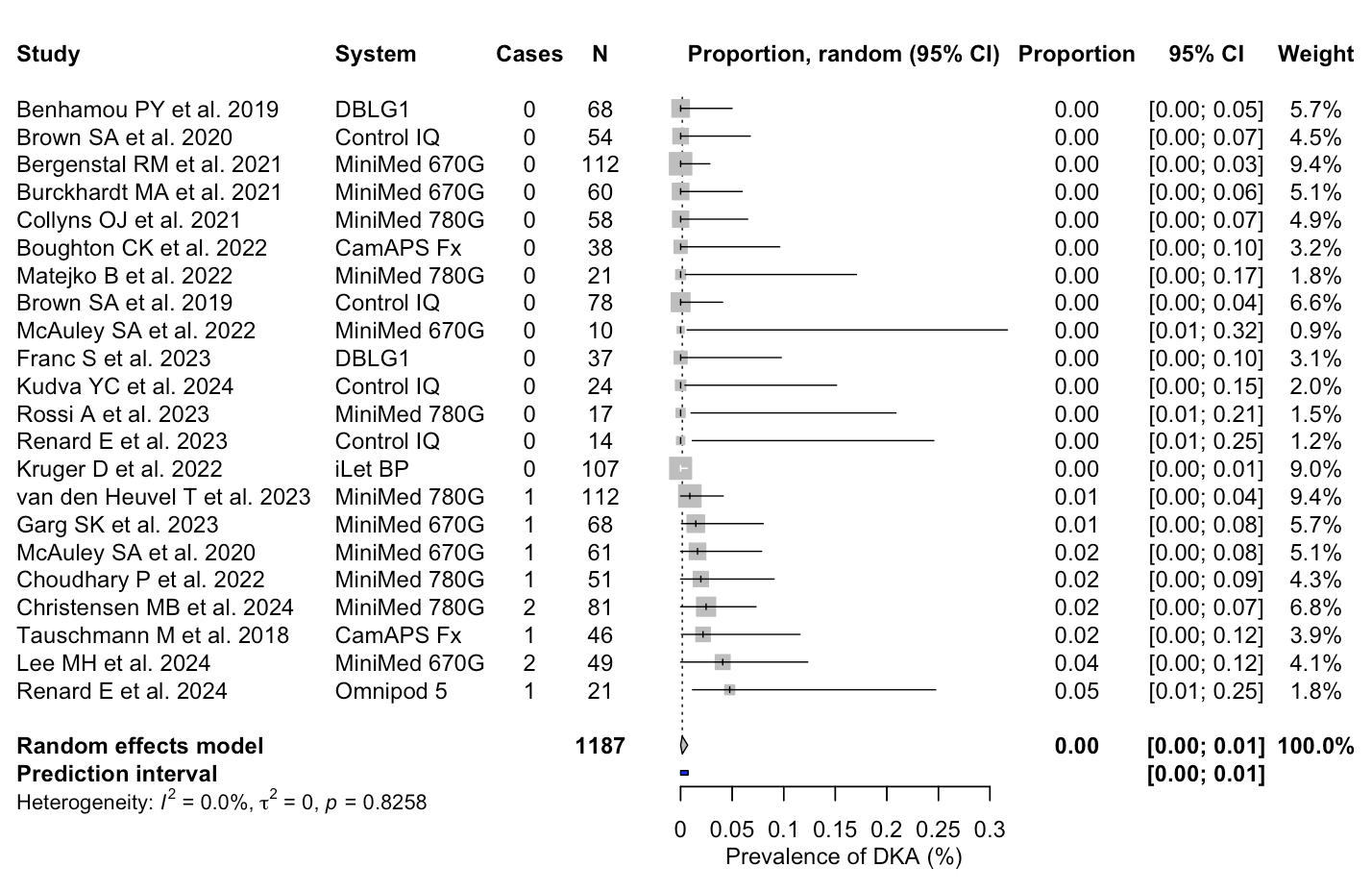
**Each forest plot displays the pooled mean estimate (for continuous outcomes) or pooled mean proportion (for binary outcomes) with corresponding 95% confidence intervals for each study, based on random-effects meta-analyses. The overall pooled estimate and its prediction interval are reported at the bottom of each plot. Measures of heterogeneity and subgroup differences are also provided when applicable. The dashed vertical line represents the overall pooled effect estimate, while the solid vertical line (when shown) corresponds to reference thresholds derived from international consensus statements or clinical guidelines. These thresholds are available for Time in Range (TIR), Time Below Range (TBR and TBR <54 mg/dL), Time Above Range (TAR), coefficient of variation (CV), and HbA1c. No specific reference values were applied for mean glucose, severe hypoglycemia, or diabetic ketoacidosis (DKA).

#

# Supplement 6. Meta-regression-adjusted forest plots for Time In Range

#

## 6.1 Studies with mean age <18 years

### 6.1.1 TIR adjusted at HbA1c 7.5%
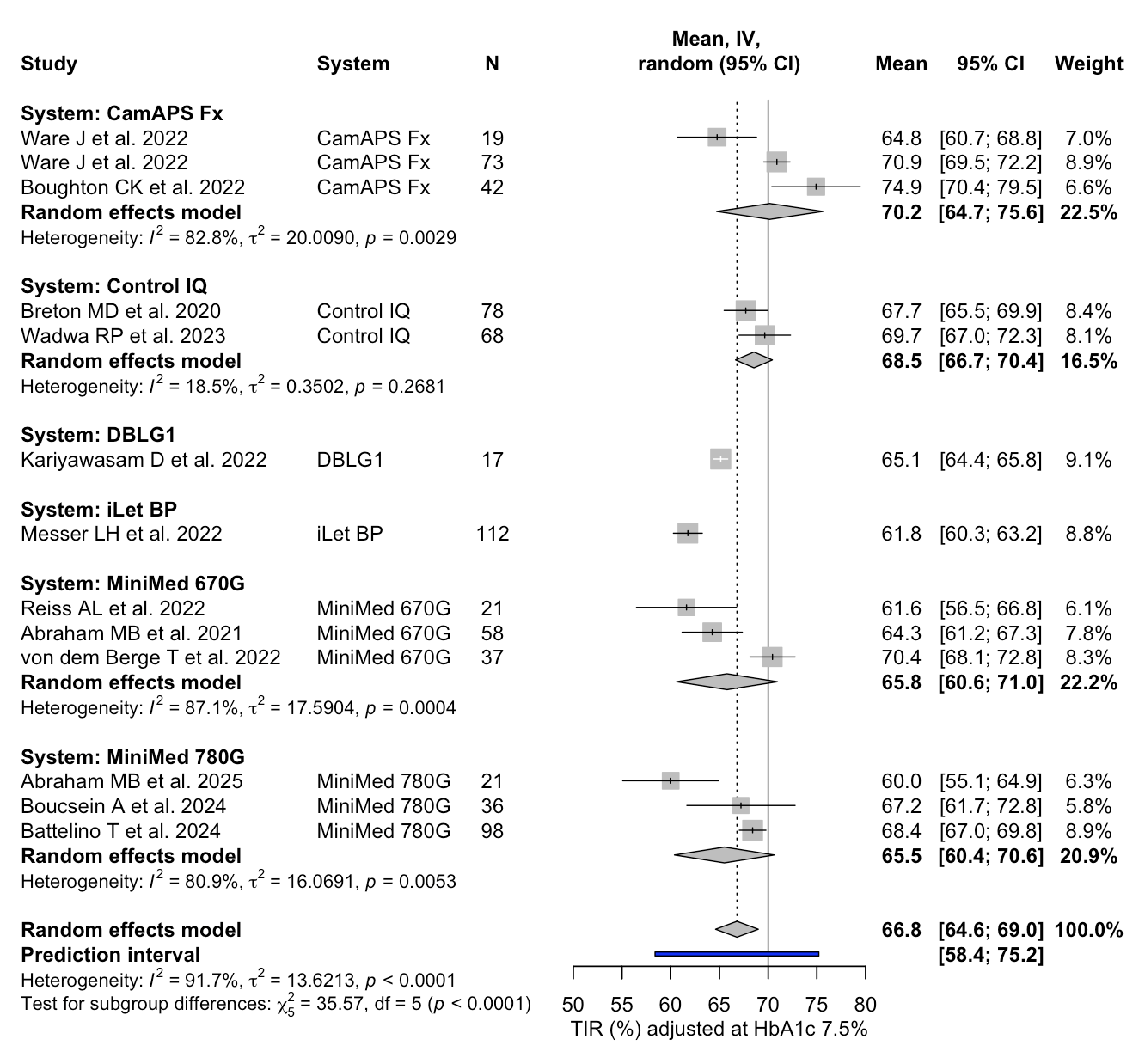


#

#

#

#

### 6.1.2 TIR adjusted at HbA1c 8%
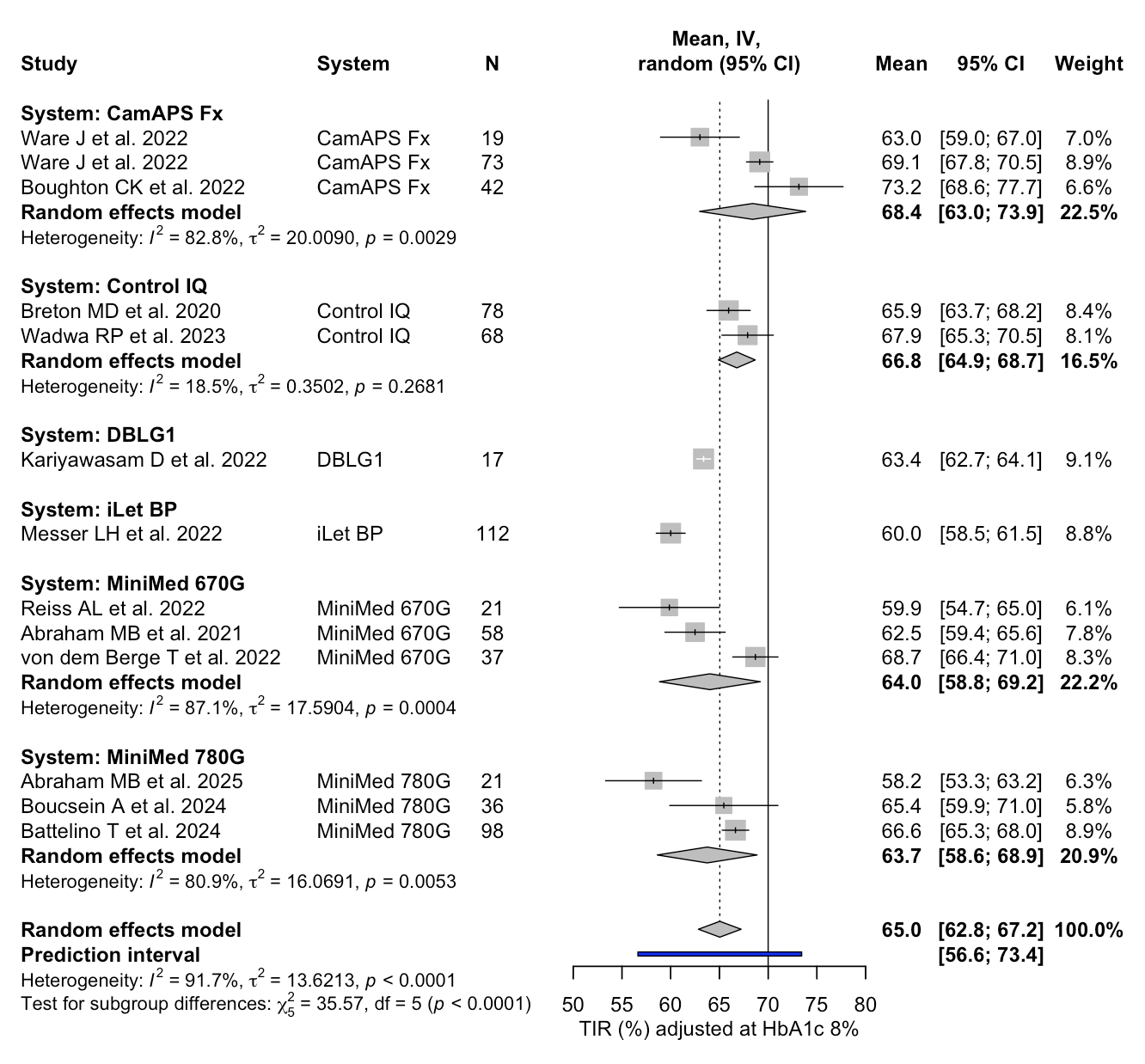


### 6.1.3 TIR adjusted at HbA1c 8.5%
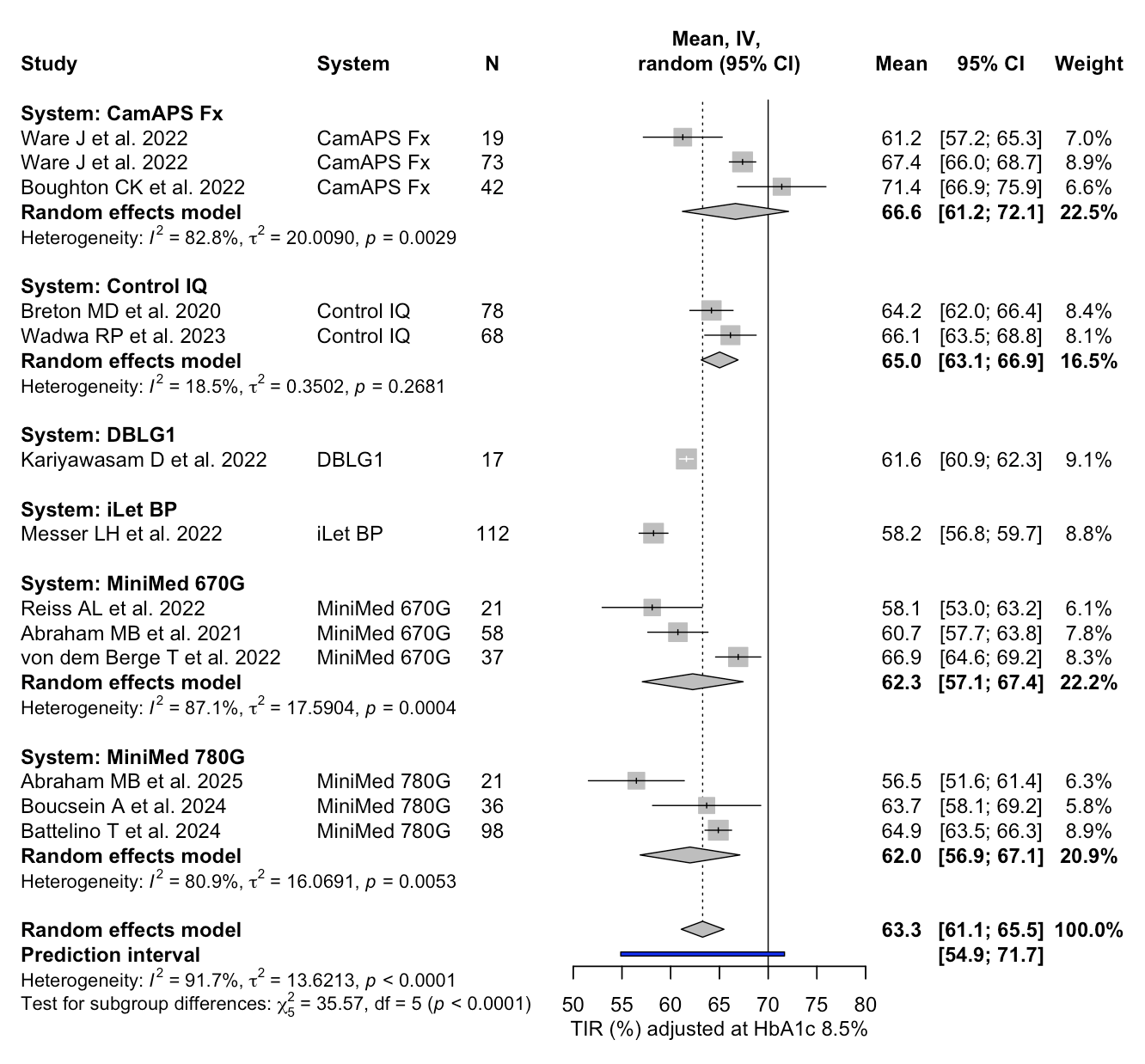


Meta-regression-adjusted forest plots for Time In Range (TIR) values standardized to baseline HbA1c levels of 7.5%, 8%, and 8.5%, with subgroup analyses by hybrid closed-loop system, based on study populations with a mean age <18 years. Each plot displays the pooled mean TIR with 95% confidence intervals for each study, along with the overall random-effects estimate and corresponding prediction interval. Measures of heterogeneity and subgroup differences are reported at the bottom of each panel. The dashed vertical line represents the overall effect estimate, while the solid vertical line indicates the reference TIR threshold of 70%, as suggested by international consensus and clinical guidelines.

#

## 6.2 Studies with mean age ≥18 years

### 6.2.1 TIR adjusted at HbA1c 7.5%

**
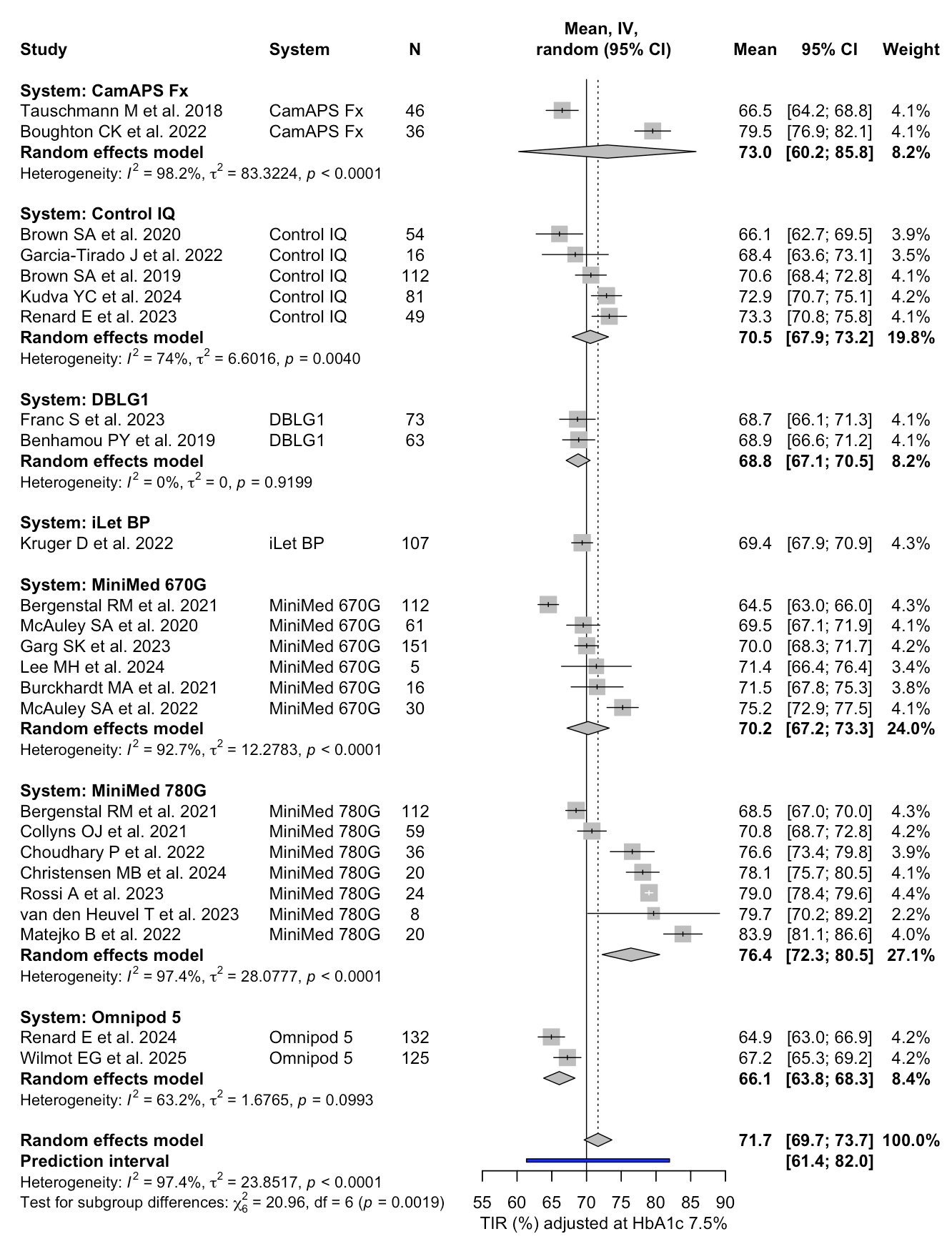
**

### 6.2.2 TIR adjusted at HbA1c 8%**
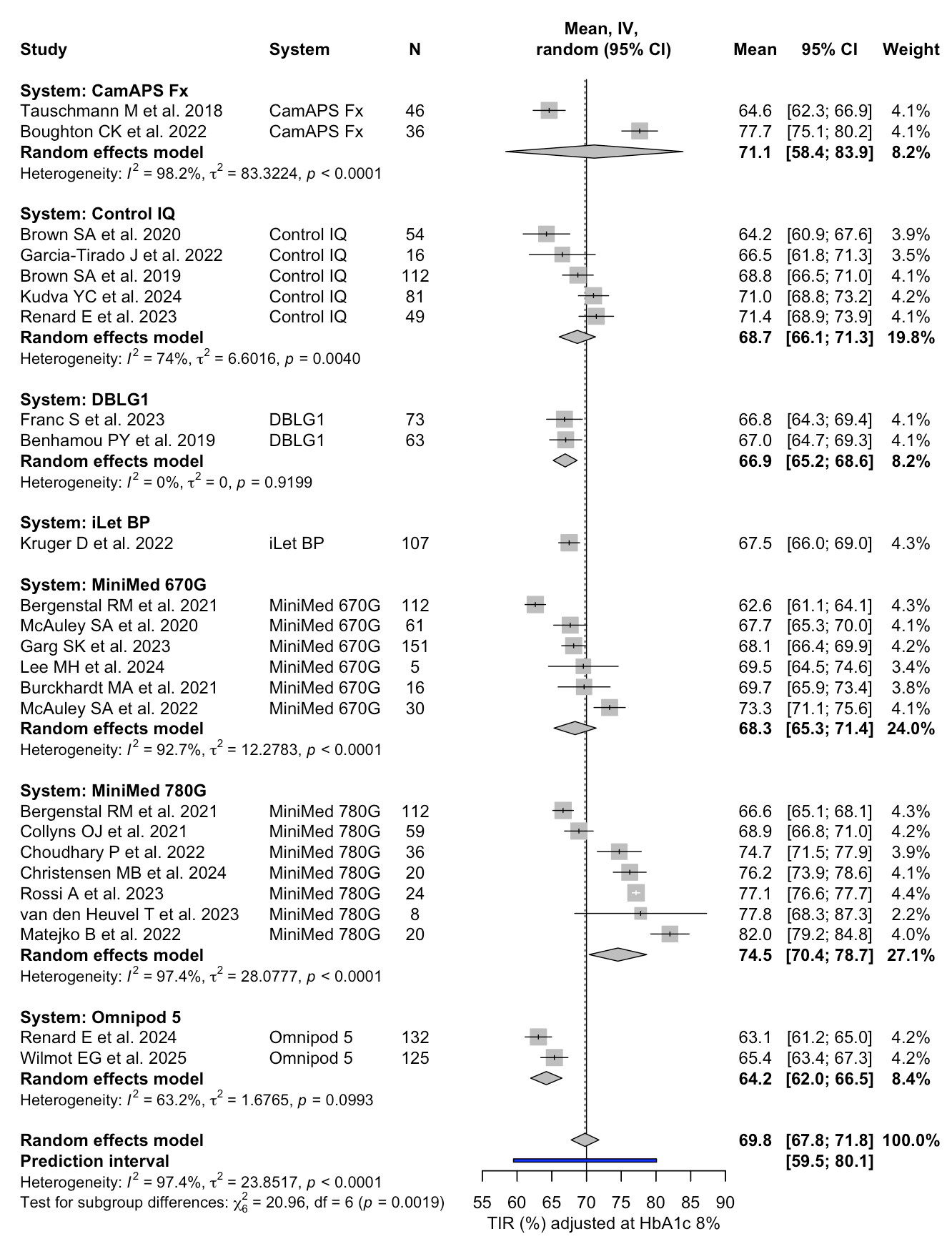
**

### 6.2.3 TIR adjusted at HbA1c 8.5%
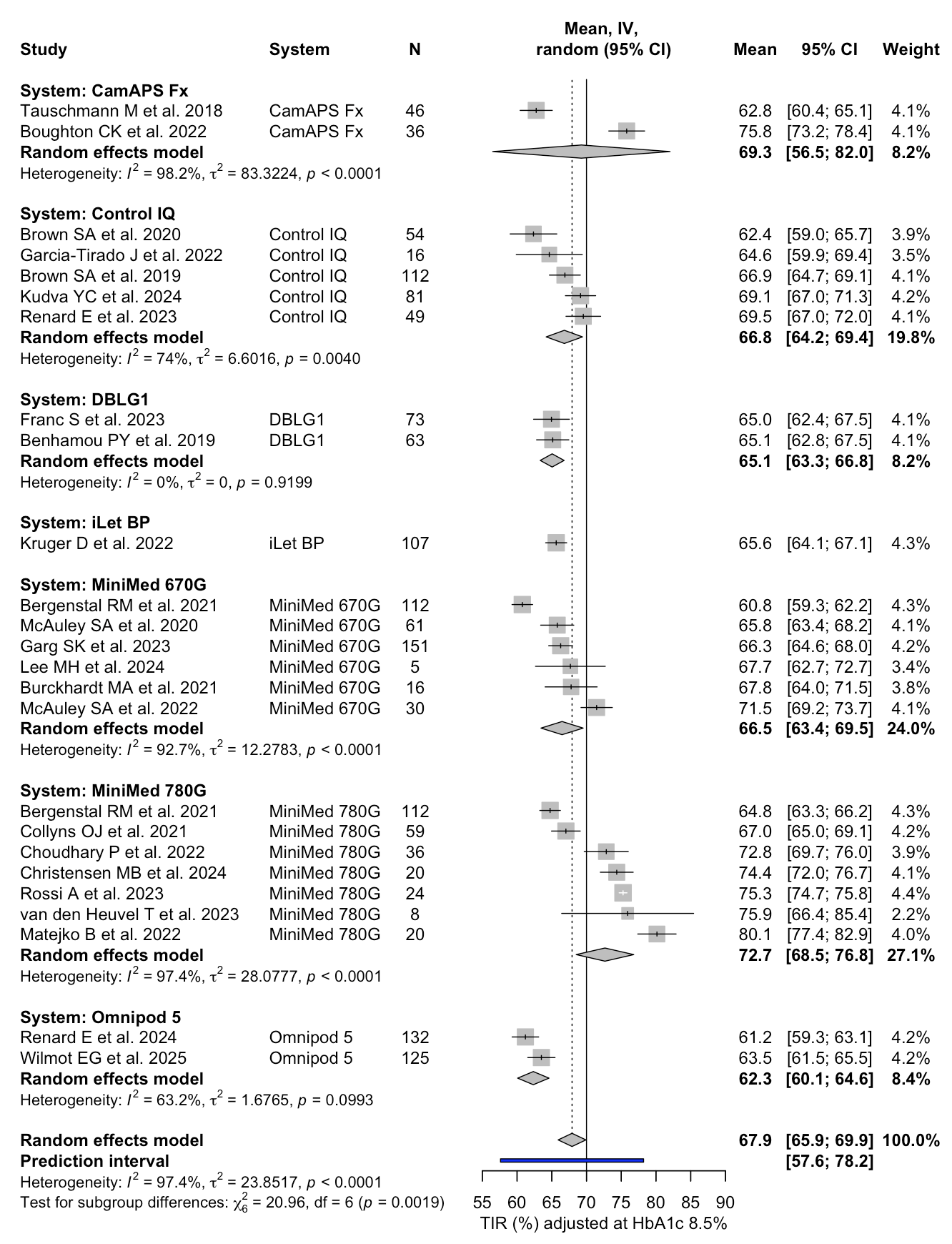


Meta-regression-adjusted forest plots for Time In Range values standardized to baseline HbA1c levels of 7.5%, 8%, and 8.5%, with subgroup analyses by hybrid closed-loop system, based on study populations with a mean age ≥18 years. Each plot displays the pooled mean TIR with 95% confidence intervals for each study, along with the overall random-effects estimate and corresponding prediction interval. Measures of heterogeneity and subgroup differences are reported at the bottom of each panel. The dashed vertical line represents the overall effect estimate, while the solid vertical line indicates the reference TIR threshold of 70%, as suggested by international consensus and clinical guidelines.

# Supplement 7. Sensitivity analysis of Time in Range excluding trials with fewer than 10 participants

## 7.1 Funnel plots

### 7.1.1 Overall analysis


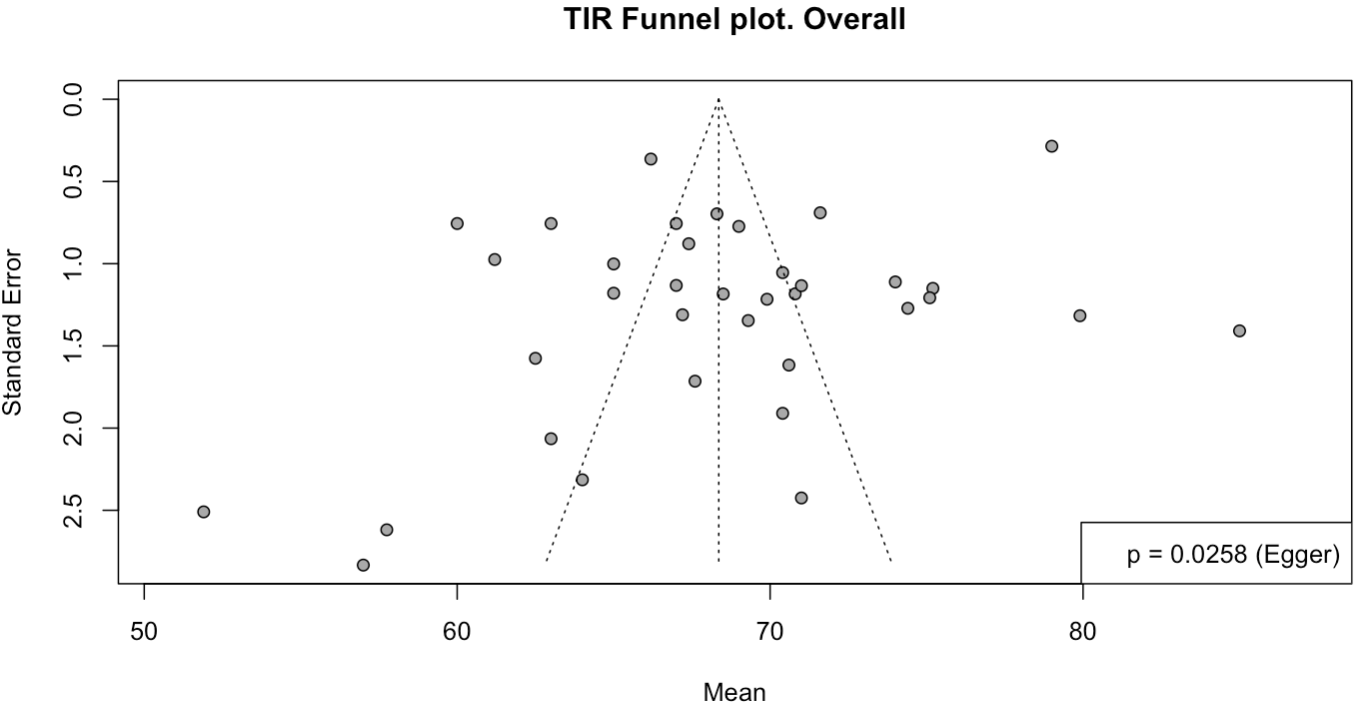


### 7.1.2 Studies with mean age ≥18 years


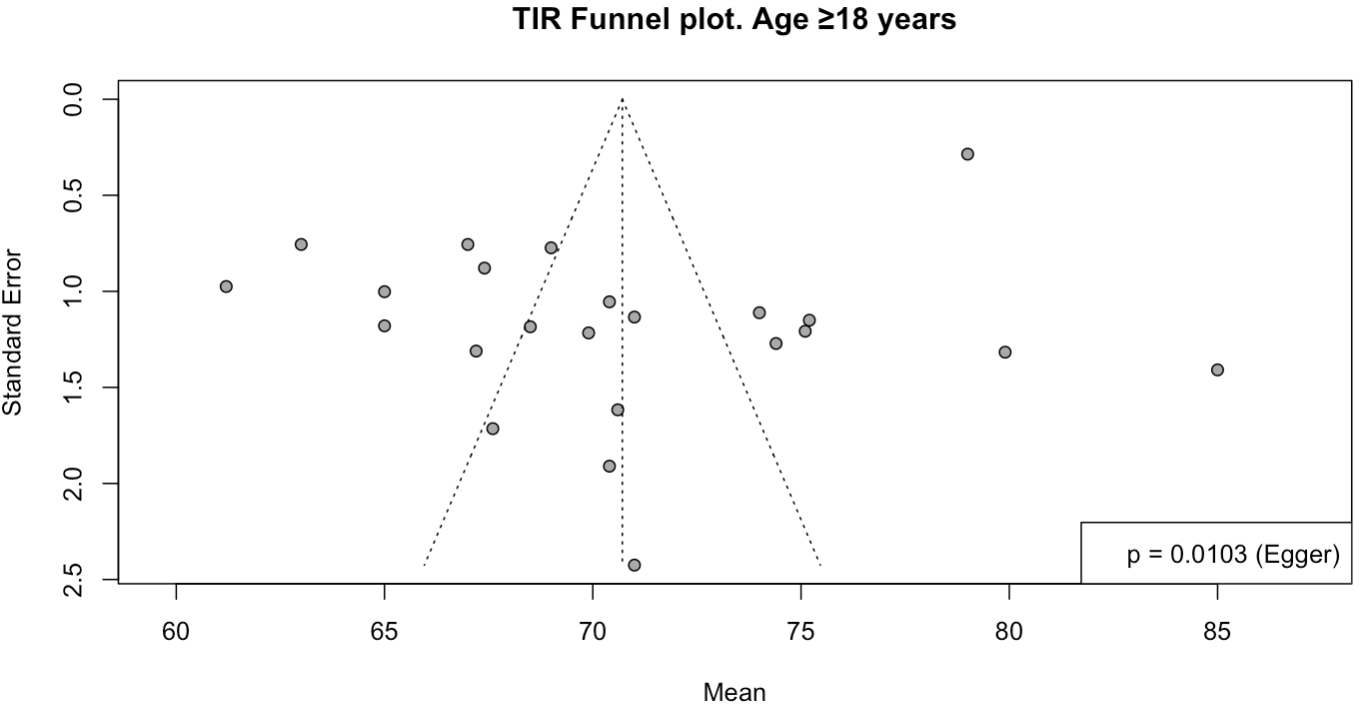


## 7.2 Forest plots

### 7.2.1 TIR stratified by age
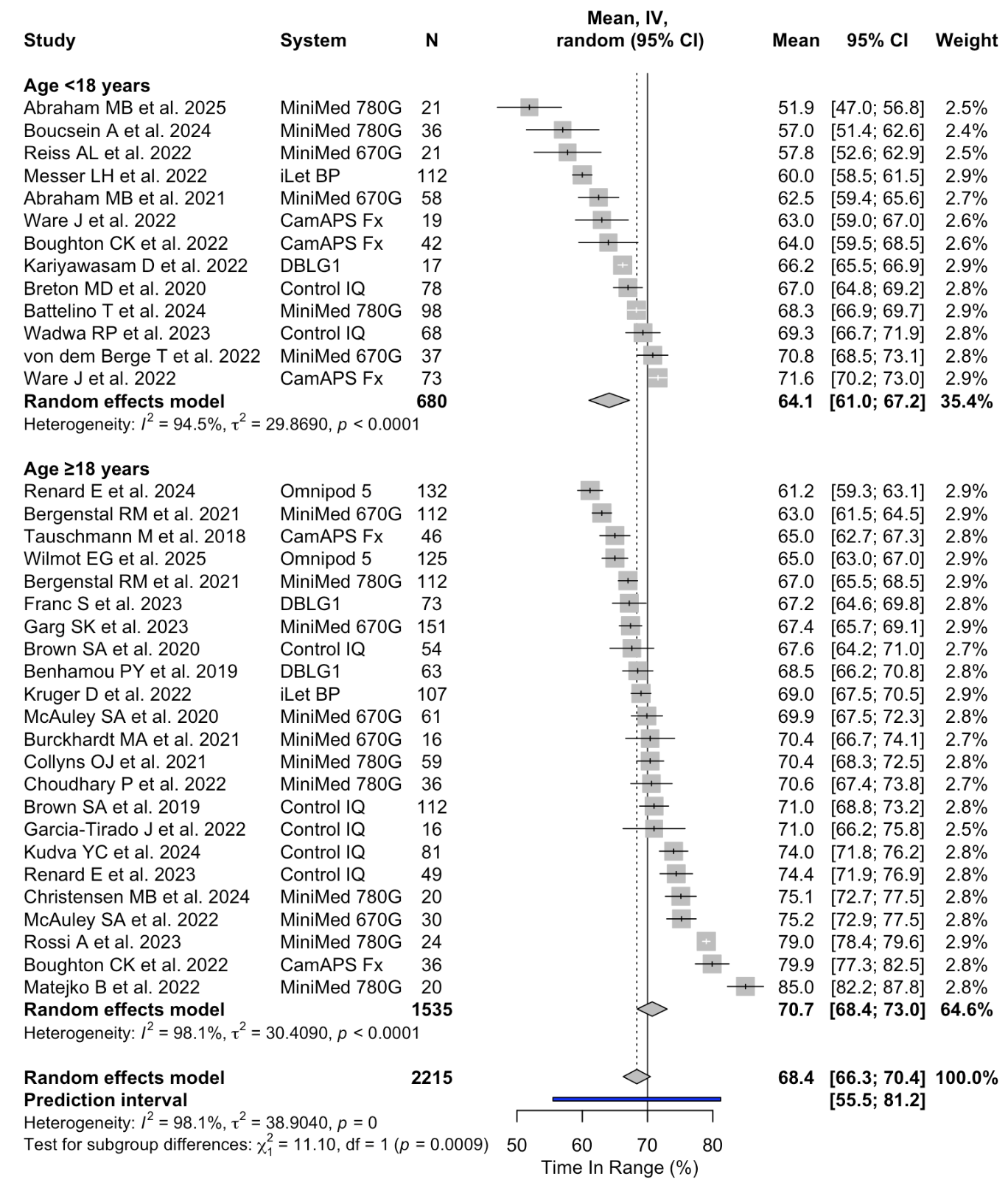


### 7.2.2 TIR stratified by baseline HbA1c
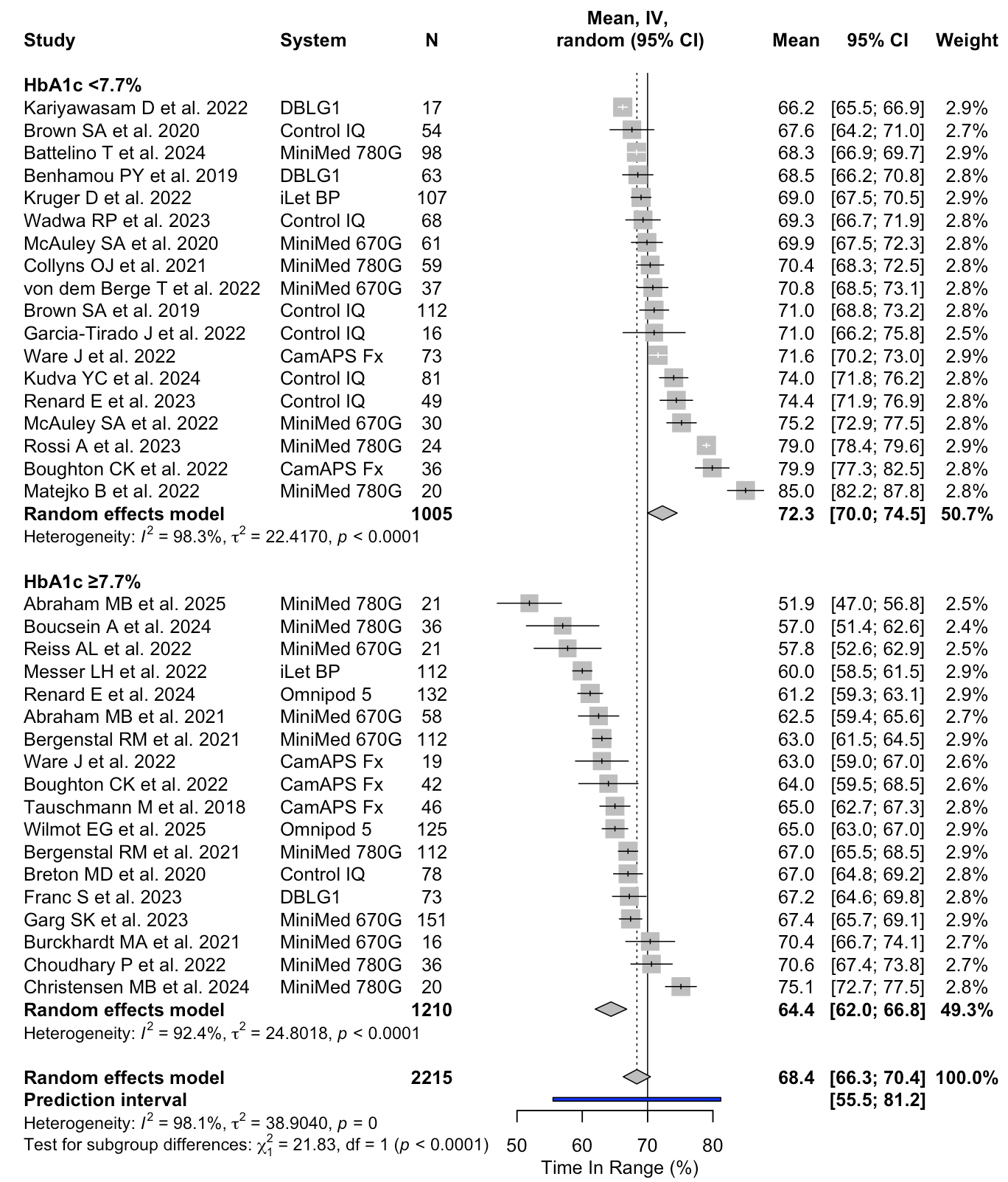


###

### 7.2.3 TIR studies with mean age ≥18 years

#

#
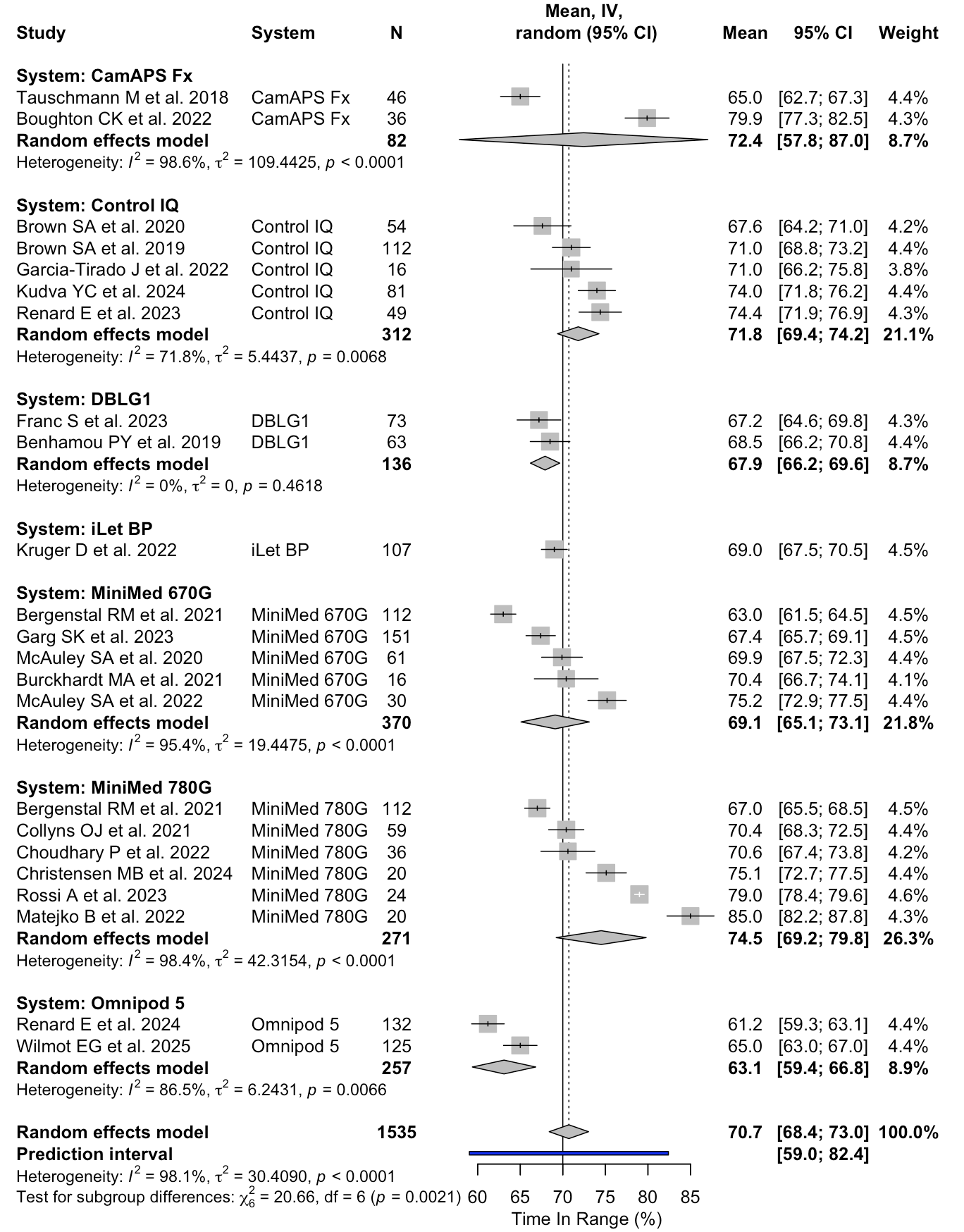


Each forest plot displays the pooled mean estimate with corresponding 95% confidence intervals for each study, based on random-effects meta-analyses. The overall pooled estimate and its prediction interval are reported at the bottom of each plot. Measures of heterogeneity and subgroup differences are also provided when applicable. The dashed vertical line represents the overall pooled effect estimate, while the solid vertical line corresponds to reference thresholds derived from international consensus statements or clinical guidelines.

# References

1. [Tauschmann M, Thabit H, Bally L, et al. Closed-loop insulin delivery in suboptimally controlled type 1 diabetes: a multicentre, 12-week randomised trial. *Lancet*. 2018;392(10155):1321-1329.](http://paperpile.com/b/4MUUKd/IbVu)

2. [Benhamou PY, Franc S, Reznik Y, et al. Closed-loop insulin delivery in adults with type 1 diabetes in real-life conditions: a 12-week multicentre, open-label randomised controlled crossover trial. *Lancet Digit Health*. 2019;1(1):e17-e25.](http://paperpile.com/b/4MUUKd/1701)

3. [Brown SA, Kovatchev BP, Raghinaru D, et al. Six-month randomized, multicenter trial of closed-loop control in type 1 diabetes. *N Engl J Med*. 2019;381(18):1707-1717.](http://paperpile.com/b/4MUUKd/nHol)

4. [Breton MD, Kanapka LG, Beck RW, et al. A randomized trial of closed-loop control in children with type 1 diabetes. *N Engl J Med*. 2020;383(9):836-845.](http://paperpile.com/b/4MUUKd/KCYJ)

5. [Brown SA, Beck RW, Raghinaru D, et al. Glycemic outcomes of use of CLC versus PLGS in type 1 diabetes: A randomized controlled trial. *Diabetes Care*. 2020;43(8):1822-1828.](http://paperpile.com/b/4MUUKd/01Bv)

6. [McAuley SA, Lee MH, Paldus B, et al. Six months of hybrid closed-loop versus manual insulin delivery with fingerprick blood glucose monitoring in adults with type 1 diabetes: A randomized, controlled trial. *Diabetes Care*. 2020;43(12):3024-3033.](http://paperpile.com/b/4MUUKd/H50G)

7. [Abraham MB, de Bock M, Smith GJ, et al. Effect of a hybrid closed-loop system on glycemic and psychosocial outcomes in children and adolescents with type 1 diabetes: A randomized clinical trial: A randomized clinical trial. *JAMA Pediatr*. 2021;175(12):1227-1235.](http://paperpile.com/b/4MUUKd/WJtC)

8. [Bergenstal RM, Nimri R, Beck RW, et al. A comparison of two hybrid closed-loop systems in adolescents and young adults with type 1 diabetes (FLAIR): a multicentre, randomised, crossover trial. *Lancet*. 2021;397(10270):208-219.](http://paperpile.com/b/4MUUKd/16UO)

9. [Burckhardt MA, Abraham MB, Dart J, et al. Impact of hybrid closed loop therapy on hypoglycemia awareness in individuals with type 1 diabetes and impaired hypoglycemia awareness. *Diabetes Technol Ther*. 2021;23(7):482-490.](http://paperpile.com/b/4MUUKd/HaBa)

10. [Collyns OJ, Meier RA, Betts ZL, et al. Improved glycemic outcomes with Medtronic MiniMed Advanced Hybrid Closed-Loop delivery: Results from a randomized crossover trial comparing automated insulin delivery with predictive low glucose suspend in people with type 1 diabetes. *Diabetes Care*. 2021;44(4):969-975.](http://paperpile.com/b/4MUUKd/9oEX)

11. [Boughton CK, Hartnell S, Thabit H, et al. Hybrid closed-loop glucose control compared with sensor augmented pump therapy in older adults with type 1 diabetes: an open-label multicentre, multinational, randomised, crossover study. *Lancet Healthy Longev*. 2022;3(3):e135-e142.](http://paperpile.com/b/4MUUKd/6rAA)

12. [Boughton CK, Allen JM, Ware J, et al. Closed-loop therapy and preservation of C-peptide secretion in type 1 diabetes. *N Engl J Med*. 2022;387(10):882-893.](http://paperpile.com/b/4MUUKd/esoy)

13. [Choudhary P, Kolassa R, Keuthage W, et al. Advanced hybrid closed loop therapy versus conventional treatment in adults with type 1 diabetes (ADAPT): a randomised controlled study. *Lancet Diabetes Endocrinol*. 2022;10(10):720-731.](http://paperpile.com/b/4MUUKd/ItQZ)

14. [Garcia-Tirado J, Farhy L, Nass R, et al. Automated insulin delivery with SGLT2i combination therapy in type 1 diabetes. *Diabetes Technol Ther*. 2022;24(7):461-470.](http://paperpile.com/b/4MUUKd/HTIo)

15. [Kariyawasam D, Morin C, Casteels K, et al. Hybrid closed-loop insulin delivery versus sensor-augmented pump therapy in children aged 6-12 years: a randomised, controlled, cross-over, non-inferiority trial. *Lancet Digit Health*. 2022;4(3):e158-e168.](http://paperpile.com/b/4MUUKd/0Sz4)

16. [Kruger D, Kass A, Lonier J, et al. A multicenter randomized trial evaluating the insulin-only configuration of the bionic pancreas in adults with type 1 diabetes. *Diabetes Technol Ther*. 2022;24(10):697-711.](http://paperpile.com/b/4MUUKd/ti4U)

17. [Matejko B, Juza A, Kieć-Wilk B, et al. Transitioning of people with type 1 diabetes from multiple daily injections and self-monitoring of blood glucose directly to MiniMed 780G advanced hybrid closed-loop system: A two-center, randomized, controlled study. *Diabetes Care*. 2022;45(11):2628-2635.](http://paperpile.com/b/4MUUKd/Vyli)

18. [McAuley SA, Trawley S, Vogrin S, et al. Closed-loop insulin delivery versus sensor-augmented pump therapy in older adults with type 1 diabetes (ORACL): A randomized, crossover trial. *Diabetes Care*. 2022;45(2):381-390.](http://paperpile.com/b/4MUUKd/O3w5)

19. [Messer LH, Buckingham BA, Cogen F, et al. Positive impact of the bionic pancreas on diabetes control in youth 6-17 years old with type 1 diabetes: A multicenter randomized trial. *Diabetes Technol Ther*. 2022;24(10):712-725.](http://paperpile.com/b/4MUUKd/alSQ)

20. [Reiss AL, Jo B, Arbelaez AM, et al. A Pilot randomized trial to examine effects of a hybrid closed-loop insulin delivery system on neurodevelopmental and cognitive outcomes in adolescents with type 1 diabetes. *Nat Commun*. 2022;13(1):4940.](http://paperpile.com/b/4MUUKd/LVFB)

21. [von dem Berge T, Remus K, Biester S, et al. In-home use of a hybrid closed loop achieves time-in-range targets in preschoolers and school children: Results from a randomized, controlled, crossover trial. *Diabetes Obes Metab*. 2022;24(7):1319-1327.](http://paperpile.com/b/4MUUKd/RSc6)

22. [Ware J, Allen JM, Boughton CK, et al. Randomized trial of closed-loop control in very young children with type 1 diabetes. *N Engl J Med*. 2022;386(3):209-219.](http://paperpile.com/b/4MUUKd/tDbn)

23. [Ware J, Boughton CK, Allen JM, et al. Cambridge hybrid closed-loop algorithm in children and adolescents with type 1 diabetes: a multicentre 6-month randomised controlled trial. *Lancet Digit Health*. 2022;4(4):e245-e255.](http://paperpile.com/b/4MUUKd/XpwF)

24. [ClinicalTrials.gov. Accessed June 22, 2025.](http://paperpile.com/b/4MUUKd/iodz) <https://clinicaltrials.gov/study/NCT04190277?term=SP8&intr=Diabeloop&rank=1>

25. [Garg SK, Grunberger G, Weinstock R, et al. Improved glycemia with hybrid closed-loop versus continuous subcutaneous insulin infusion therapy: Results from a randomized controlled trial. *Diabetes Technol Ther*. 2023;25(1):1-12.](http://paperpile.com/b/4MUUKd/CdWg)

26. [Renard E, Joubert M, Villard O, et al. Safety and efficacy of sustained automated insulin delivery compared with sensor and pump therapy in adults with type 1 diabetes at high risk for hypoglycemia: A randomized controlled trial. *Diabetes Care*. 2023;46(12):2180-2187.](http://paperpile.com/b/4MUUKd/pPvl)

27. [ClinicalTrials.gov. Accessed June 22, 2025.](http://paperpile.com/b/4MUUKd/KRR6) <https://clinicaltrials.gov/study/NCT05477030>

28. [Media Centre. Accessed June 22, 2025.](http://paperpile.com/b/4MUUKd/RBNr) <https://www.easd.org/media-centre/#!resources/automated-insulin-delivery-tightens-glucose-control-and-stabilises-circulating-endothelial-progenitor-cells-in-a-6-months-randomised-study-29c1b3fb-77f0-4a68-9e29-12495c7b19e1>

29. [van den Heuvel T, Kolassa R, Keuthage W, et al. Advanced hybrid closed loop in Adult Population with type 1 diabetes: A substudy from the ADAPT randomized controlled trial in users of real-time continuous glucose monitoring. *J Diabetes Sci Technol*. 2024;18(5):1132-1138.](http://paperpile.com/b/4MUUKd/I8nv)

30. [Wadwa RP, Reed ZW, Buckingham BA, et al. Trial of hybrid closed-loop control in young children with type 1 diabetes. *N Engl J Med*. 2023;388(11):991-1001.](http://paperpile.com/b/4MUUKd/vIUa)

31. [ClinicalTrials.gov. Accessed June 22, 2025.](http://paperpile.com/b/4MUUKd/5kmu) <https://clinicaltrials.gov/study/NCT05574062?tab=history>

32. [Battelino T, Kuusela S, Shetty A, et al. Efficacy and safety of automated insulin delivery in children aged 2–6 years (LENNY): an open-label, multicentre, randomised, crossover trial. *Lancet Diabetes Endocrinol*. 2025;0(0). doi:](http://paperpile.com/b/4MUUKd/mYBK)[10.1016/s2213-8587(25)00091-9](http://dx.doi.org/10.1016/s2213-8587(25)00091-9)

33. [Boucsein A, Zhou Y, Michaels V, et al. Automated insulin delivery for young people with type 1 diabetes and elevated A1c. *NEJM Evid*. 2024;3(10):EVIDoa2400185.](http://paperpile.com/b/4MUUKd/b3xy)

34. [Christensen MB, Ranjan AG, Rytter K, McCarthy OM, Schmidt S, Nørgaard K. Automated insulin delivery in adults with type 1 diabetes and suboptimal HbA1c during prior use of insulin pump and continuous glucose monitoring: A randomized controlled trial. *J Diabetes Sci Technol*. Published online April 11, 2024:19322968241242399.](http://paperpile.com/b/4MUUKd/RipL)

35. [Kudva YC, Henderson RJ, Kanapka LG, et al. Automated insulin delivery in older adults with type 1 diabetes. *NEJM Evid*. 2025;4(1):EVIDoa2400200.](http://paperpile.com/b/4MUUKd/saQx)

36. [Lee MH, Gooley J, Obeyesekere V, et al. Hybrid closed loop in adults with type 1 diabetes and severely impaired hypoglycemia awareness. *J Diabetes Sci Technol*. Published online April 13, 2024:19322968241245627.](http://paperpile.com/b/4MUUKd/6T8G)

37. [Renard E, Weinstock RS, Aleppo G, et al. Efficacy and safety of a tubeless AID system compared with pump therapy with CGM in the treatment of type 1 diabetes in adults with suboptimal glycemia: A randomized, parallel-group clinical trial. *Diabetes Care*. 2024;47(12):2248-2257.](http://paperpile.com/b/4MUUKd/JQd1)

38. [Abraham MB, Smith GJ, Dart J, et al. Glycemic and psychosocial outcomes of advanced hybrid closed-loop therapy in youth with high HbA1c: A randomized clinical trial. *Diabetes Care*. 2025;48(1):67-75.](http://paperpile.com/b/4MUUKd/OTg0)

39. [ClinicalTrials.gov. Accessed June 22, 2025.](http://paperpile.com/b/4MUUKd/C0FB) <https://clinicaltrials.gov/study/NCT05923827?term=NCT05923827&rank=1>
